# Network-driven discovery of repurposable drugs targeting hallmarks of aging

**DOI:** 10.1038/s43587-026-01161-8

**Published:** 2026-06-26

**Authors:** Bnaya Gross, Joseph Ehlert, Vadim N. Gladyshev, Joseph Loscalzo, Albert-László Barabási

**Affiliations:** 1 Network Science Institute, Northeastern University, Boston, MA USA; 2 Department of Physics, Northeastern University, Boston, MA USA; 3 Department of Medicine, Brigham and Women’s Hospital, Harvard Medical School, Boston, MA USA; 4 Department of Network and Data Science, Central European University, Vienna, Austria

**Keywords:** Computational models, Statistical methods, Computer modelling

## Abstract

Despite the thousands of genes implicated in age-related phenotypes, effective interventions for aging remain elusive, due to the multifactorial nature of longevity and the interconnectedness of molecular components involved. Here we introduce a network medicine framework to map 2,358 longevity-associated genes onto the human interactome to identify drug-repurposing candidates capable of modulating specific hallmarks of aging. We find that genes associated with each hallmark form a connected subgraph, or hallmark module, allowing us to measure the network proximity of 6,442 compounds to each hallmark. We then introduce a transcription-based metric, pAGE, which evaluates whether drug-induced expression shifts reinforce or counteract known age-related expression changes within each hallmark module. By integrating network proximity and pAGE, we identify drug-repurposing candidates targeting specific hallmarks and provide a falsifiable framework to leverage genomic discoveries for accelerating drug repurposing in longevity. Our findings are interpretable, revealing molecular mechanisms through which drugs modulate hallmarks.

## Abstract (web summary)

The authors introduce a network medicine framework showing that the hallmarks of aging form interconnected molecular modules in the human interactome. This new approach can help to identify existing drugs that might influence aging-associated transcriptional changes.

## Main

Over the past decade, comprehensive genetic surveys^1–5^ and systematic animal experiments^6,7^ have implicated thousands of human genes in age-related phenotypes, with particular focus on the key biological processes underlying age-related functional decline, known as hallmarks of aging^8–10^. While these efforts offer unprecedented opportunities to dissect the molecular basis of longevity^11–14^, we continue to lack treatments and interventions capable of modulating specific aging processes. This shortfall potentially stems from the multifactorial nature of aging and the functional and mechanistic interconnectedness of the molecular and genetic processes implicated in longevity, limiting the impact of any single intervention.

The multifactorial nature of longevity is often formalized through the ‘hallmarks of aging’, which demarcate multiple distinct age-related mechanisms, ranging from genomic instability to cellular senescence^8–10^. Although each hallmark is intended to represent a distinct biological dimension, they are not fully distinct, and extensive cross-talk and synergy exist among them. Yet, current therapeutic interventions in clinical trials typically target only one or at most a few facets of aging. A comprehensive strategy for promoting longevity will likely require multiple interventions, each addressing different mechanisms (or hallmarks) of aging. Developing novel compounds to achieve this is a lengthy endeavor requiring a decade or more to reach clinical practice. An attractive alternative is to repurpose from the pool of over 6,000 clinically approved or experimental drugs, because some of these agents might effectively target specific aging processes^15–17^. Indeed, most of these compounds have already undergone toxicity screening and possess well-characterized targets (and have known side effects), allowing for more rapid clinical development. The key challenge is to identify the compounds that can influence longevity—and specifically, the hallmark they target and the relevant molecular mechanism.

To address this bottleneck in aging research, here we introduce a network medicine framework^18–21^ that allows us to integrate data on thousands of aging-associated genes, along with their network relationships, and the targets of all approved and experimental drugs, aiming to identify potential interventions that could affect longevity. Specifically, we begin with a library of 2,358 previously identified longevity-associated genes, which we map onto the human interactome. Strikingly, we find that specific hallmark-associated genes aggregate into a connected subgraph, forming distinct and statistically significant hallmark modules. The discovery of these modules is our first discovery, enabling us to apply established network medicine approaches for drug repurposing^22–24^ and to evaluate the proximity of 6,442 clinically approved or experimental compounds to each hallmark module, thereby identifying candidates capable of perturbing specific aging phenotypes.

Our second key advance is the introduction of a novel transcription-based metric, Pro-Age (pAGE), that allows us to assess whether the expression changes induced by a drug reinforce or counteract known age-related expression changes, allowing us to distinguish beneficial interventions from those that may accelerate aging. This advance was missing in prior repurposing efforts^22–24^, which only predicted the drug’s impact on a given phenotype but not the directionality and the magnitude of its action. Ultimately, we find multiple drugs that successfully reverse the expression changes observed during aging in specific hallmarks, representing promising repurposing candidates. Our predictions are interpretable, revealing the precise molecular mechanisms by which each drug-repurposing candidate modulates the specific hallmark of aging, thereby providing experimentally falsifiable hypotheses. Together, our study offers a principled, integrative route to leverage the vast body of aging-related knowledge to identify drugs that can address the multifactorial nature of aging.

## The genetic roots and interconnectivity of the hallmarks of aging

**Fig. 1:**
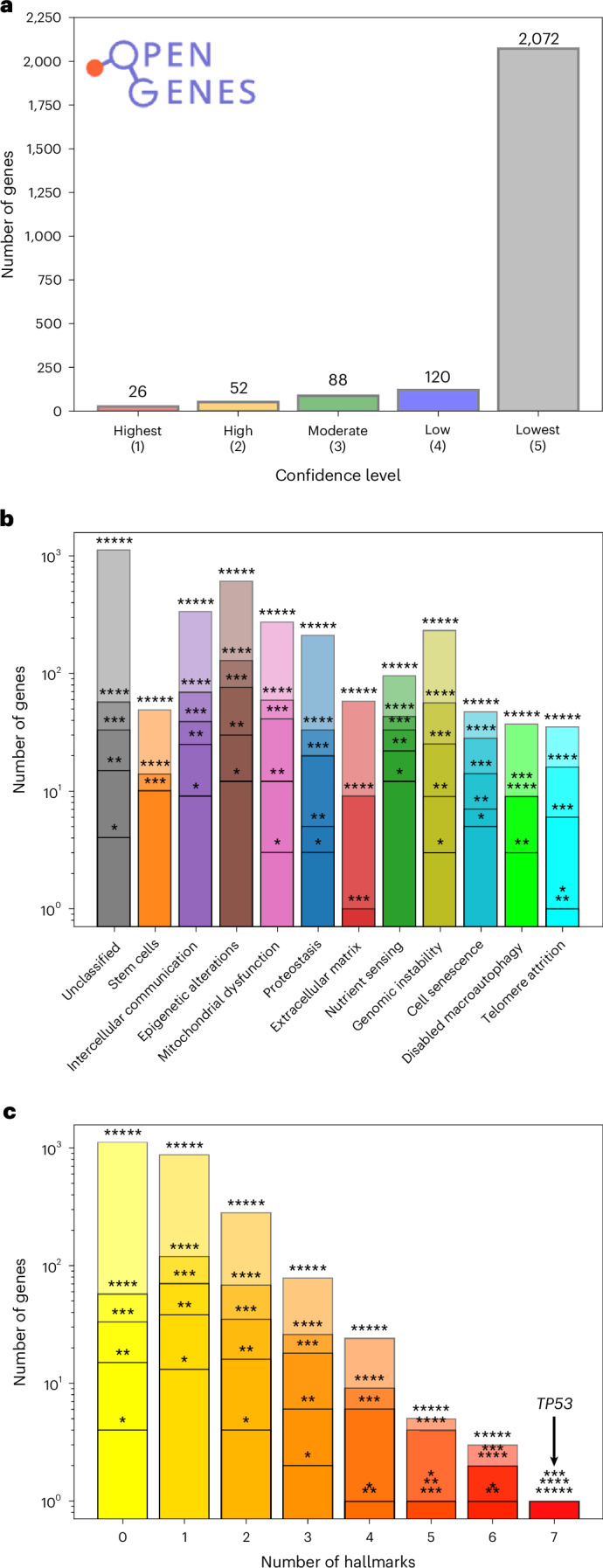
Aging-associated genes. **a**, The OpenGenes database contains 2,358 aging-associated genes, each gene being assigned a confidence level ranging from 1 (highest) to 5 (lowest) based on the existing evidence of its association with lifespan and longevity (see Supplementary Section I for the classification protocol). While only 26 genes have the highest confidence level, indicating that changes in their activity extend mammalian lifespan, most genes have low confidence and show a weak association with aging. **b**, The number of genes associated with each of the hallmarks of aging; 1,250 genes are associated by the OpenGenes database with one or more hallmarks of aging based on their biological role, while the remaining 1,108 genes are unclassified. Asterisks indicate the number of genes with a specific confidence level (1–5) associated with each hallmark. Note that the ‘exhaustion of stem cells’ and ‘changes in the extracellular matrix structure’ hallmarks are not associated with any of the genes in levels 1 and 2, while the ‘disabled macroautophagy’ hallmark is not associated with any of the genes in level 1. **c**, The number of genes associated with multiple hallmarks. Reflecting the interconnectedness between the hallmarks, some of the genes are associated with multiple hallmarks; 1,108 genes are not linked to any hallmark, 860 genes are linked to a single hallmark, and 390 genes are shared by multiple hallmarks. The *TP53* gene is associated with the most (7) hallmarks, reflecting its critical roles in various essential cellular processes such as DNA repair and apoptosis.

**Extended Data Fig. 1:**
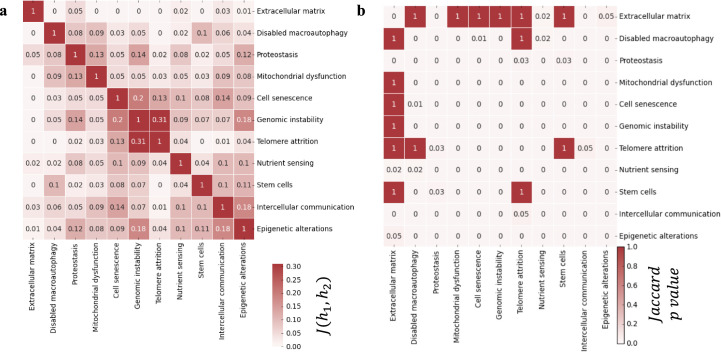
Jaccard index of the hallmarks of aging. **a**, The Jaccard index measures the interconnectivity between pairs of hallmarks of aging. The hallmarks of Genomic instability, cell senescence, and Telomere attrition show the highest Jaccard value (center) while the Changes in the extracellular matrix structure hallmark show almost no overlap with the other hallmarks. **b**, The p-value of the Jaccard index are statistically significant (p-value < 0.05) between overlapping hallmarks.

We began by querying the OpenGenes database^25^, which manually curates gene-level annotations that link 2,358 genes to longevity, age-associated diseases or pathways implicated in aging, also offering a confidence level for each association (Fig. 1a and Supplementary Section I), ranging from 1 (highest) to 5 (lowest). From this resource, we identify the genes that are explicitly linked to at least one of the 11 hallmarks of aging^8–10^ (Fig. 1b). Among the 2,358 longevity-associated genes, 1,250 can be associated with specific hallmarks of aging: 860 are exclusive to a single hallmark, and 390 span multiple hallmarks (Fig. 1c). The remaining 1,108 genes, while linked to aging, could not be associated with a specific hallmark based on the current knowledge of their biological function. We can, however, rely on the topology of the human interactome to link these genes to specific hallmarks (Supplementary Section III). The 390 multi-hallmark genes support our hypothesis that, at the molecular level, the hallmarks of aging are not independent entities. Further support is offered by pairwise comparisons using the Jaccard index^26^, revealing statistically significant gene overlap among 47 of the 55 hallmark pairs (Extended Data Fig. 1 and Supplementary Section IV).

Although the OpenGenes database offers evidence extracted from observational and experimental studies that link each gene to its assigned hallmark of aging, we wished to confirm whether the collective set of 1,250 hallmark-associated genes is broadly relevant to longevity. To this end, we performed additional validation (Supplementary Section II and Methods), finding that the set of 1,250 genes shows significant enrichment in (i) age-related KEGG pathways^27^ (Supplementary Fig. 11), (ii) genes implicated in aging by six large-scale aging studies^3–5,28–30^ (Supplementary Table 1), (iii) five aging-related diseases (Supplementary Table 2), (iv) eight distinct cancer types whose incidence increasing significantly with age (Supplementary Fig. 4) and (v) genes involved in DNA repair or progeroid syndromes^31–33^ (Supplementary Fig. 5). We checked that none of these validation datasets were included in the development of the OpenGenes database (Supplementary Section II). Hence, these enrichments lend additional support to the aging relevance of this gene set, serving as the foundation for our subsequent work.

## The hallmark modules of aging

**Fig. 2:**
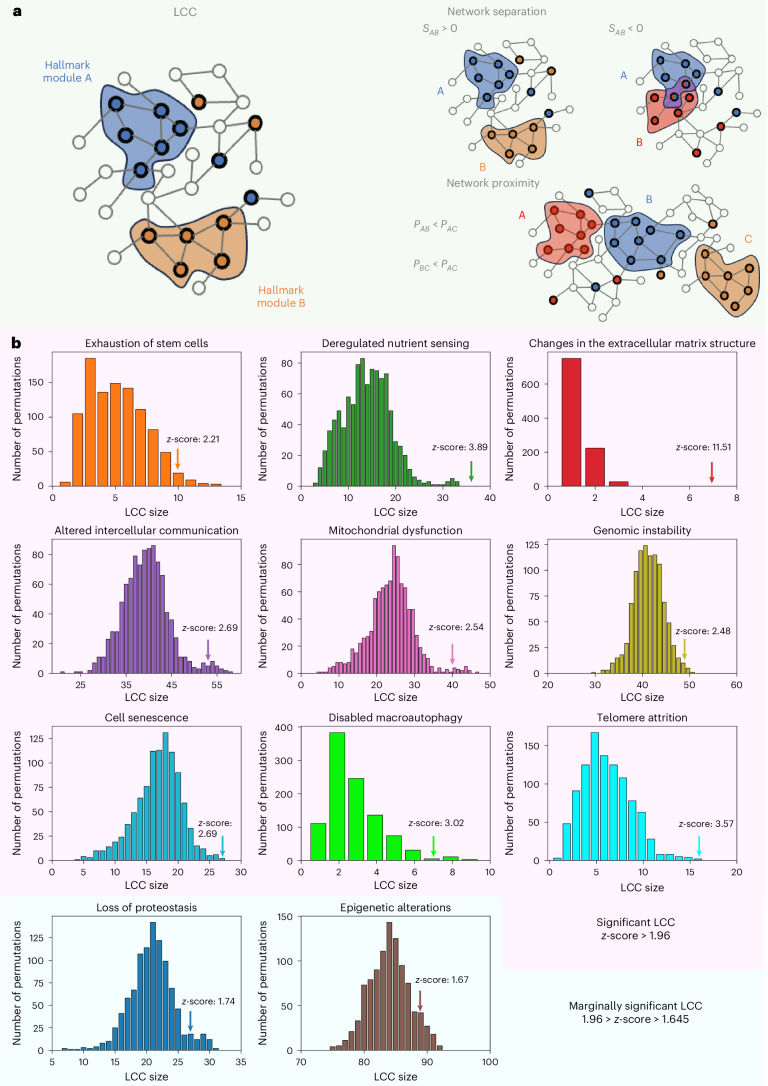
Network characteristics of the hallmarks of aging. **a**, Genes associated with similar biological mechanisms often form connected components^18^. The LCC characterizes the module of the gene set. The network structure allows us to analyze the network separation and proximity between different gene sets. Negative separation indicates overlapping modules while positive separation denotes distinct, topologically nonoverlapping modules. Network proximity estimates the network-based distance between modules utilizing the shortest paths between pairs of genes in different modules. The proximity allows for estimating modules in close neighborhoods compared with distant ones. **b**, The LCC size and significance of each hallmark of aging compared with the distribution of LCCs formed by a randomized control group (Methods). The LCC formed by the genes of each hallmark defines the ‘hallmark module’, characterizing the network representation of the hallmark. The genes of each hallmark of aging form a statistically significant LCC (defined as *z*-score > 1.96) compared with the control group. The only two exceptions are the ‘loss of proteostasis’ hallmark (*z*-score = 1.74) and the ‘epigenetic alterations’ hallmark (*z*-score = 1.67), which display marginal significance (defined as *z*-score > 1.645).

To capture the network-level organization of aging, we mapped the 1,250 hallmark-associated genes onto the human interactome—a comprehensive catalog of 524,156 experimentally validated binding interactions among 18,223 proteins (Methods). For many diseases and phenotypes, the genes associated with the disease are known to coalesce in the interactome to form a ‘disease module’, formally defined as the largest connected component (LCC) formed by the disease genes^18^. While disease modules were validated for multiple isolated traits^34–37^, aging comprises multiple hallmarks that may each behave as distinct, yet interrelated, phenotypes. It is thus unclear whether the different hallmark genes form independent modules, and if these modules reside in the same network neighborhood (Fig. 2a).

**Fig. 3:**
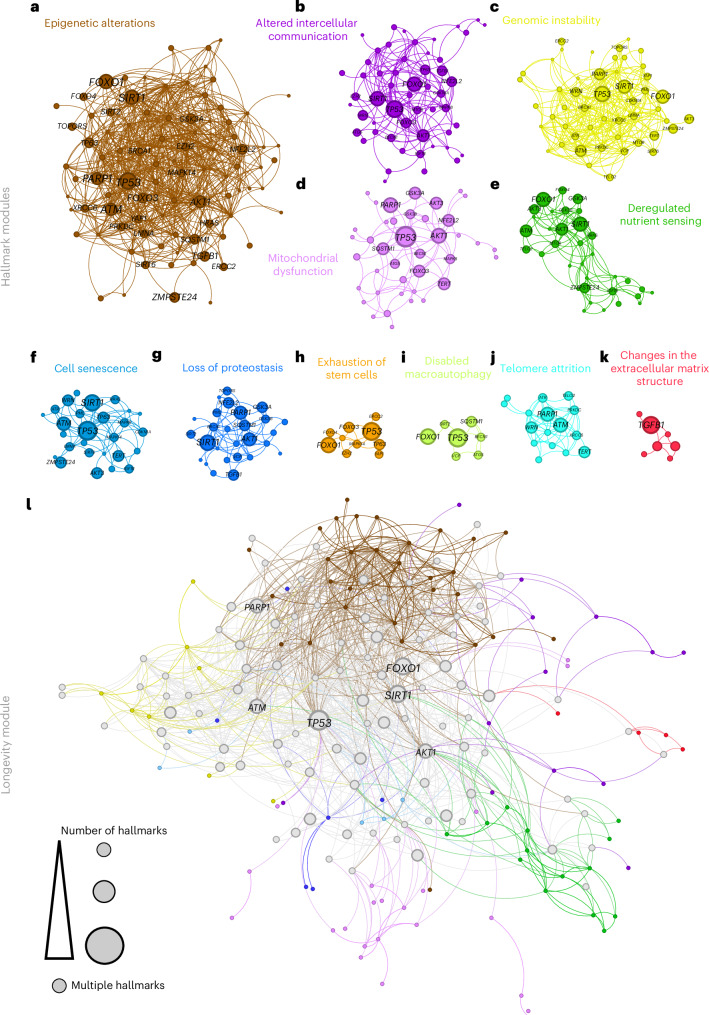
The hallmark modules and the longevity module. **a**–**k**, Genes associated with each of the hallmarks of aging are not randomly distributed in the human interactome but agglomerate in a specific network neighborhood, forming a statistically significant LCC. These LCCs are the ‘hallmark modules’—subgraphs of the human interactome representing the biological origin of the hallmarks of aging. Each hallmark module is shown separately with a distinct color. Genes associated with more than a single hallmark are shown with a label. **a**, Epigenetic alterations (89 genes). **b**, Altered intercellular communication (53 genes). **c**, Genomic instability (49 genes). **d**, Mitochondrial dysfunction (40 genes). **e**, Deregulated nutrient sensing (34 genes). **f**, Cell senescence (27 genes). **g**, Loss of proteostasis (27 genes). **h**, Exhaustion of stem cells (ten genes). **i**, Disabled macroautophagy (seven genes). **j**, Telomere attrition (16 genes). **k**, Changes in the extracellular matrix structure (seven genes). **l**, The 11 hallmark modules were found to be in the same network neighborhood and when agglomerated together form the ‘longevity module’ shown here. Genes associated with a single hallmark module are colored accordingly, while genes associated with multiple hallmarks are colored in black with the node’s edges colored based on their hallmark associations. The size of each node reflects its number of hallmark associations. The labels of genes associated with five or more hallmarks are shown.

To answer these questions, we examined each hallmark separately, finding that in 9 of the 11 hallmarks, associated genes cluster into a statistically significant LCC (*z*-score > 1.96; Fig. 2b). The remaining two hallmarks—‘loss of proteostasis’ (*z*-score = 1.74) and ‘epigenetic alterations’ (*z*-score = 1.67)—also show a marginal significance, indicating that their gene sets are characterized by nonrandom connectivity. In other words, the hallmark-associated genes reside in narrowly defined network neighborhoods, each representing a distinct and identifiable ‘hallmark module’ within the global interactome (Fig. 3a–k). We note that the structure of the hallmark modules may be partially shaped by pathway-driven aging research and by biases in protein–protein interaction (PPI) networks toward well-studied genes. To address this, we performed multiple robustness validations using an unbiased high-throughput PPI, the STRING PPI and an edge-permutation significance test, consistently recovering the statistical significance of the hallmark modules (Supplementary Section XII). These findings establish that hallmark genes form well-defined and statistically significant network modules.

**Extended Data Fig. 2:**
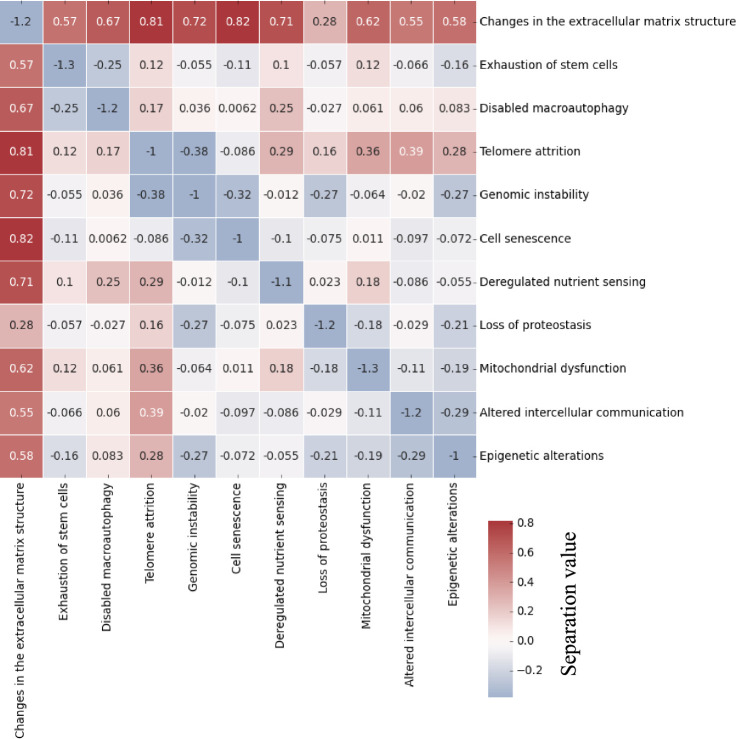
Separation of the hallmarks of aging. The network-based separation measurement between pairs of hallmarks of aging. While the separation of the Changes in the extracellular matrix structure hallmark is positive with the other hallmarks (S > 0) many other pairs of the hallmarks are overlapping (S < 0), indicating that the hallmark modules reside in the same network neighborhood, together forming the longevity module.

**Extended Data Fig. 3:**
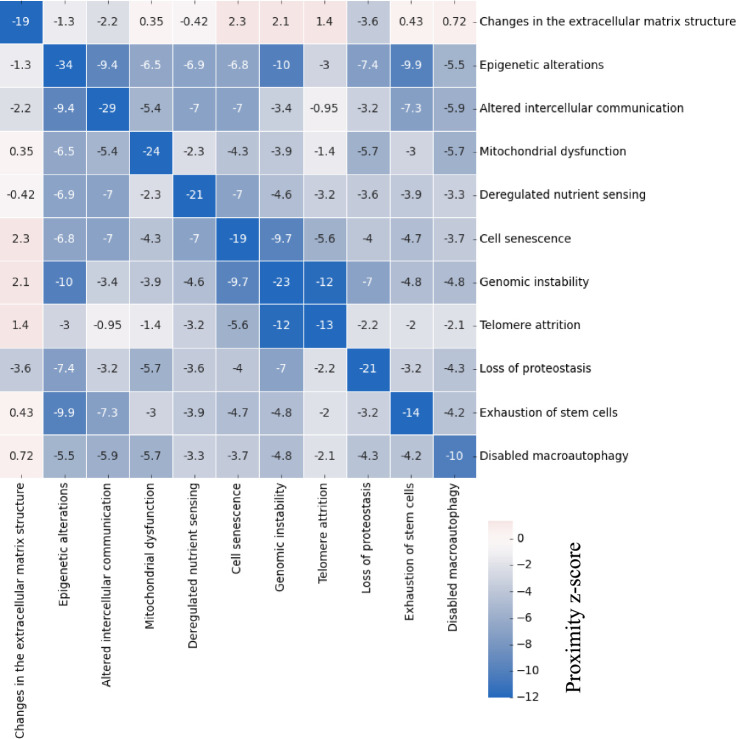
Proximity of the hallmarks of aging. The z-score of the network-based proximity measurement of most hallmarks show statistically significant proximity (z-score < −1.96) except for the Changes in the extracellular matrix structure hallmark, indicating that the hallmark modules reside in the same network neighborhood, together forming the longevity module.

We next examined whether these hallmark modules overlap, aiming to reveal functional relationships among them. To do so, we used two complementary measures: separation^18^ and proximity^22^ (Fig. 2a and Extended Data Figs. 2 and 3; see also Methods and Supplementary Section V), finding that the hallmarks of aging are located in the same neighborhood of the interactome, together forming a broader ‘longevity module’ (Fig. 3l and Supplementary Section VI). The overlap between the individual hallmark modules reflects the existence of shared molecular mechanisms underlying related hallmarks. Next, we leverage the existence of the individual hallmark modules to identify drug-repurposing candidates that target specific hallmarks.

## Network-based identification of hallmark-specific drug-repurposing candidates

The existence of the hallmark modules offers the opportunity to apply network-based drug-repurposing methods, originally designed for single-disease modules, to the more complex and multifactorial context of aging^22–24^. To this end, we compiled 6,442 approved or clinically tested compounds from DrugBank^38^. Our approach rests on the premise that drugs whose targets lie in the network proximity to a disease (or hallmark) module are poised to perturb that disease (or hallmark) with potential therapeutic outcome, a hypothesis that has been experimentally supported across multiple diseases—from asthma to heart disease, and has been experimentally validated for 6,710 drugs, successfully predicting their potential role in treating coronavirus disease 2019 (COVID-19) infection^24,34,35,39^. To develop such a framework, we begin by measuring each drug’s network proximity to every hallmark module for five sets of hallmark genes, stratified by confidence level (Fig. 1a). For each hallmark, we then ranked drugs by the significance of their proximity (*z*-score < −1.96). For example, we find 27 compounds that displayed statistically significant proximity to the ‘cell senescence’ hallmark at every confidence level (Fig. 7). The top-ranked candidate in this list, pimasertib, is a MEK1/MEK2 inhibitor known to induce apoptosis and senescence^40^, aligning with its predicted effect. Another high-ranking compound, selisistat, a SIRT1 inhibitor, also promotes senescence^41^. These examples demonstrate that proximity successfully detects compounds previously implicated in senescence. Yet they also show that proximity alone does not imply a beneficial (anti-senescence) effect.

**Fig. 4:**
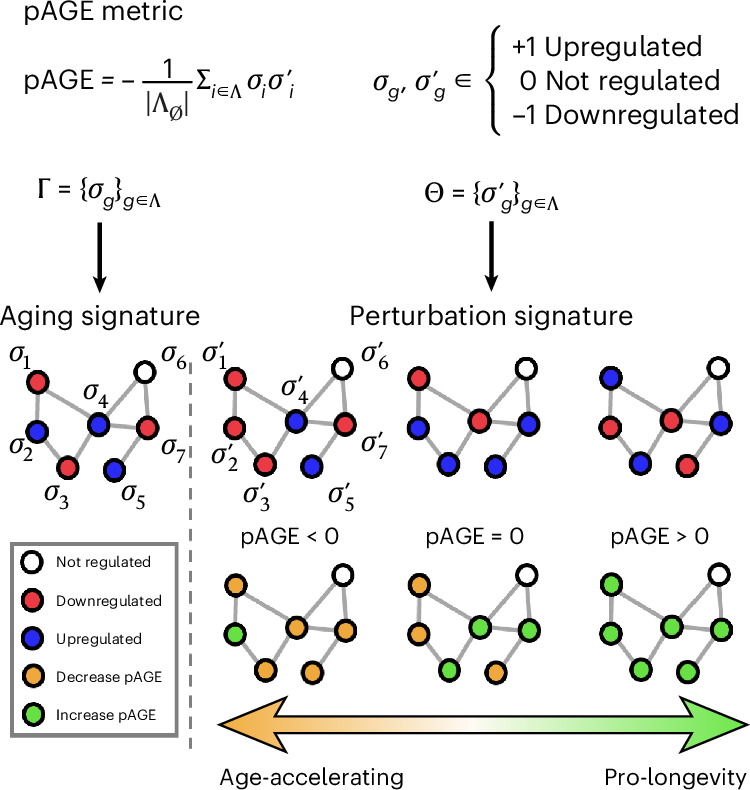
The pAGE metric for quantifying age-accelerating and pro-longevity perturbations. The schematic illustrates how the pAGE value is computed by comparing an aging signature ($\Gamma$) with a perturbation signature ($\Theta$) across hallmark genes. Upregulated, downregulated and not regulated nodes are encoded and summed to yield the pAGE value (equation (3)), where pAGE < $0$ indicates an age-accelerating effect and pAGE > 0 indicates a pro-longevity effect. The color key shows nodes that do not change (white), are downregulated (red), are upregulated (blue), decrease pAGE (orange) or increase pAGE (green).

The network-based approach relies on undirected protein interactions, which can successfully establish a compound’s ability to perturb a module, but carries no information on whether the perturbation is beneficial or detrimental. To address this limitation, here we introduce a metric called pAGE (Fig. 4 and Methods), which quantifies whether drug-induced changes in gene expression reinforce or counter documented age-related expression shifts. Specifically, if a drug upregulates a gene that is known to be upregulated with age, one might anticipate a potentially adverse effect on longevity, whereas downregulating the same gene may be advantageous (Fig. 4).

In summary, proximity predicts a drug’s potential impact on a hallmark module, while pAGE predicts the directionality of that effect: positive pAGE values indicate shifts toward younger expression patterns, whereas negative values suggest potential age-accelerating effects (Fig. 4). Based on this rationale, we propose SHARP (systematic hallmark-based aging repurposing pipeline), which first uses network proximity to identify compounds targeting hallmark-related subgraphs, and then uses pAGE to determine whether the induced transcriptional changes are pro-longevity or age-accelerating.

Note, however, that not all age-related expression changes are detrimental. For example, several stress-response pathways become activated with age yet may still confer adaptive benefits through hormesis^42,43^. Nevertheless, as we show next, SHARP successfully distinguishes lifespan-extending from non-extending compounds, indicating that such adaptive responses have only a limited confounding effect on predictive performance.

## Validation of the SHARP repurposing pipeline

To validate SHARP and assess its limitations, we examined its ability to identify compounds supported by experimental and clinical evidence for longevity. Specifically, we evaluated drugs shown to extend lifespan in mice^44^ and compounds currently under clinical investigation for longevity^45^. To estimate the false-positive rate, we also analyzed compounds tested in mice that failed to extend lifespan^44^. Because low-confidence genes (levels 3–5) introduce substantial noise into the pAGE metric (Supplementary Fig. 20), validation analyses were restricted to high-confidence genes (levels 1–2). SHARP classified a drug as a repurposing candidate if it displayed significant proximity together with positive pAGE for at least one hallmark.

### Validation of lifespan-extending drugs in mice from the intervention testing program

**Fig. 5:**
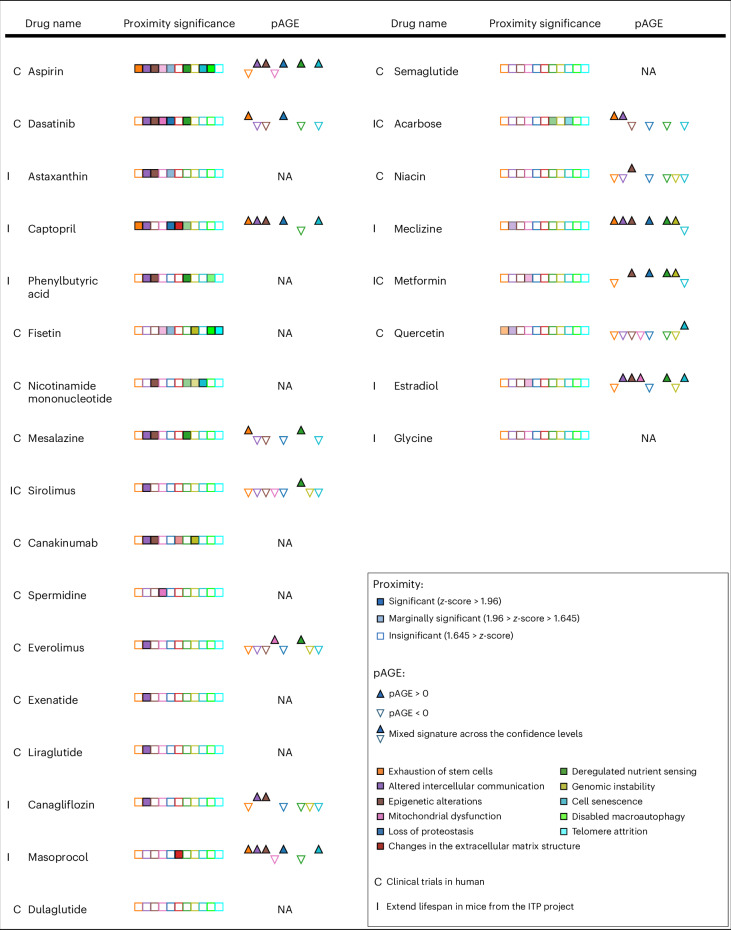
Drug repurposing of drugs currently under clinical trials for antiaging and from the ITP project. Twenty-five drugs currently under clinical trials for antiaging medicine for humans (C)^45^ or found to extend lifespan in mice from the ITP project (I)^44^. Among them, 16 showed statistically significant proximity (*z*-score < −1.96) for at least one hallmark. An additional five show marginal significance (*z*-score < −1.645). All drugs show positive pAGE for at least one hallmark. Statistically significant proximity is shown with full color and marginal significance proximity with transparent color. Nonsignificant proximity is shown in white. Arrowheads indicate the pAGE directionality (positive, up; negative, down). Proximity is measured across all confidence levels, and the most significant result is shown. pAGE is shown for level 2 or, if no high-confidence hallmark genes are present, for level 3. NA, not applicable.

We first examined 11 drugs that the intervention testing program (ITP)^44^ found to prolong lifespan in mice (Fig. 5 and Supplementary Table 3). Of the 11 that extend lifespan, 6 displayed significant proximity (*z*-score < −1.96), and 4 had marginal proximity (*z*-score < −1.645) to at least one hallmark. We measure the pAGE parameter for 8 of the 11 ITP-confirmed lifespan-extending drugs^44^ with Connectivity Map (CMap) data, finding that all eight have positive pAGE for at least one hallmark (Fig. 5 and Supplementary Table 3). Since all eight also have statistically significant or marginally significant proximity to at least one hallmark, SHARP offers 100% sensitivity (95% confidence interval: 63%, 100%) for the ITP-tested drugs (see Supplementary Sections XIII and XVIII for an analysis of potential circularity in the validation process, and Supplementary Section XVII for a baseline ablation analysis evaluating the predictive power of proximity and pAGE separately).

We also tested the 17 ITP drugs that failed to extend lifespan (Supplementary Table 11). Of these, 12 displayed significant proximity (*z*-score < −1.96), and two had marginal proximity (*z*-score < −1.645) to at least one hallmark. We measure the pAGE parameter for 7 of the 17 with CMap data, finding that only three have positive pAGE and significant or marginally significant proximity for at least one hallmark (Supplementary Table 11), resulting in a 42.8% (95% confidence interval: 9.9%, 81.5%) false-positive rate for the ITP-tested drugs.

We also find that lifespan-extending ITP drugs exhibit a higher number of hallmarks with positive pAGE values relative to negative ones ($\langle {{\rm{pAG}}{\rm{E}}}^{+}\rangle /\langle {\rm{pAG}}\rm{E}^{-}\rangle =1.24$), whereas ITP drugs that failed to extend lifespan show the opposite pattern ($\langle {{\rm{pAG}}{\rm{E}}}^{+}\rangle /\langle {{\rm{pAG}}{\rm{E}}}^{-}\rangle =0.52$), as shown in Supplementary Fig. 20. These results suggest that while some negative pAGE values may reflect compensatory or protective responses, the dominant signal among lifespan-extending compounds remains positive. Indeed, even drugs that extend lifespan may show negative pAGE values for specific hallmarks. This is expected, as it is unlikely that any intervention will simultaneously reverse all hallmarks of aging. For example, masoprocol (NDGA) extends lifespan in mice, yet it exhibits a negative pAGE value for the mitochondrial dysfunction hallmark (Fig. 5). This directionality is biologically plausible, as masoprocol has been shown to induce a loss of mitochondrial membrane potential in FL5.12 murine hematopoietic cells^46^.

### Validation using drugs under clinical trials for human longevity

We next tested our drug-repurposing pipeline against a curated list of 17 compounds currently undergoing clinical trials for healthy longevity^45^, a list that included well-known candidates such as metformin and sirolimus (rapamycin; Fig. 5 and Supplementary Table 4). Three drugs (sirolimus, acarbose and metformin) overlap with the 11 compounds that extend lifespan in mice from the ITP program described above. Of these 17, we find that 11 display statistically significant proximity to at least one hallmark (*z*-score < −1.96). For instance, aspirin is predicted to influence six hallmarks, and dasatinib is predicted to affect five, whereas sirolimus (rapamycin) affects only one hallmark, specifically the ‘intercellular communication’ hallmark module. The six drugs undergoing clinical trials not captured by our pipeline had targets located relatively far from hallmark modules (proximity > 1.6; Supplementary Fig. 14). Even so, 3 of the 6 compounds, acarbose, metformin and quercetin, exhibited marginally significant proximity (*z*-score < −1.645), illustrating that partial alignment with a hallmark can still be detected for these compounds. We measure the pAGE parameter for 9 of the 17 drugs in clinical trials for aging or longevity for which CMap data are available (Methods), finding that 8 of the nine drugs displayed positive pAGE and statistically or marginally significant for at least one hallmark (Fig. 5 and Supplementary Table 4), resulting in 88.9% (95% confidence interval: 51.7%, 99.7%) sensitivity for the drugs undergoing clinical trials.

### Validation against independent experimental results

Finally, we evaluated SHARP using 10 compounds recently tested for lifespan and healthspan effects in mice in a parallel independent study^47^. This study prioritized compounds using criteria distinct from those of the current work, and its experimental findings became available only after our predictive analysis had concluded (Supplementary Section XIV). Among the eight compounds with available CMap data, vorinostat, selumetinib, LY-294002, KU-0063794 and AZD-8055 significantly improved lifespan or healthspan, whereas valdecoxib, GDC-0941 and ascorbyl-palmitate did not. The successful compounds showed statistically significant (or marginal) proximity to at least one hallmark (Supplementary Table 12), and all compounds with CMap data displayed positive pAGE for at least one hallmark, yielding 100% sensitivity (95% confidence interval: 47.8%, 100%).

Taken together, SHARP demonstrates 100% sensitivity for mouse lifespan-extending compounds and 88.9% sensitivity for clinically tested compounds. These results reinforce the predictive strength of our approach and provide confidence in the novel drug-repurposing predictions presented below. We note that larger sample sizes in future studies are expected to yield more precise estimates and reduce the confidence intervals.

## Identifying hallmark-targeted drug-repurposing candidates

**Fig. 6:**
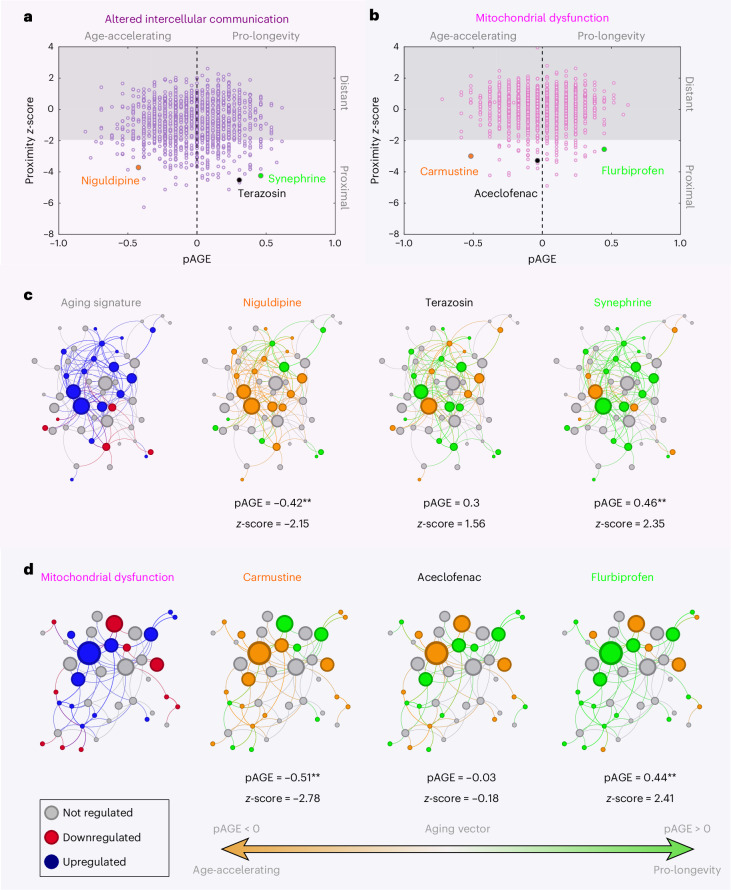
Perturbation pAGE signature. **a**,**b**, The network proximity identifies drugs with close targets to a specific hallmark module (*z*-score < −1.96), indicating a strong impact, while the pAGE values predict the direction of the impact to be beneficial (pAGE > 0) or deficient (pAGE < 0) for aging. The data points are all 1,346 drugs that appear in both the DrugBank and CMap databases and their respective proximity *z*-score and pAGE values for the ‘altered intercellular communication’ (**a**) and ‘mitochondrial dysfunction’ (**b**) hallmarks confidence level 4. The gray area indicates a lack of significance for proximity (*z*-score > −1.96). **c**,**d**, Comparing the aging signature to the drug signature allows for calculating the pAGE value (equation (3)) of drugs to ‘altered intercellular communication’ (**c**) and ‘mitochondrial dysfunction’ (**d**) hallmarks, predicting drugs that are beneficial (pAGE > 0, green) or deficient (pAGE < 0, orange) for aging. The statistical significance of the pAGE values is calculated relative to a null distribution generated from a control group of random drug signatures (Supplementary Section VIII) with two asterisks indicating statistically significant pAGE values (|*z*-score | > 1.96).

Encouraged by the positive validation above, we next apply SHARP to identify drug-repurposing opportunities for each hallmark. Specifically, we identify all approved and experimental compounds with significant proximity to specific aging modules, as identified by our network analysis, and positive pAGE value, if there is available CMap data for the compound (Fig. 6). This process allows us to identify ‘pro-longevity compounds’ that can successfully perturb a hallmark module, inducing pro-longevity expression changes. We also identify ‘age-accelerating compounds’ that can also perturb a hallmark, but the induced expression changes are more consistent with pro-aging effects. While the CMap database provides expression data for only a subset of the compounds listed in DrugBank (1,347 of 6,442), SHARP can operate with any source of drug perturbation profiles, including gene expression data available from independent studies for drugs not covered by CMap. Accordingly, the scope of the present analysis is constrained by the availability of CMap data but can be expanded using additional perturbational databases.

**Fig. 7:**
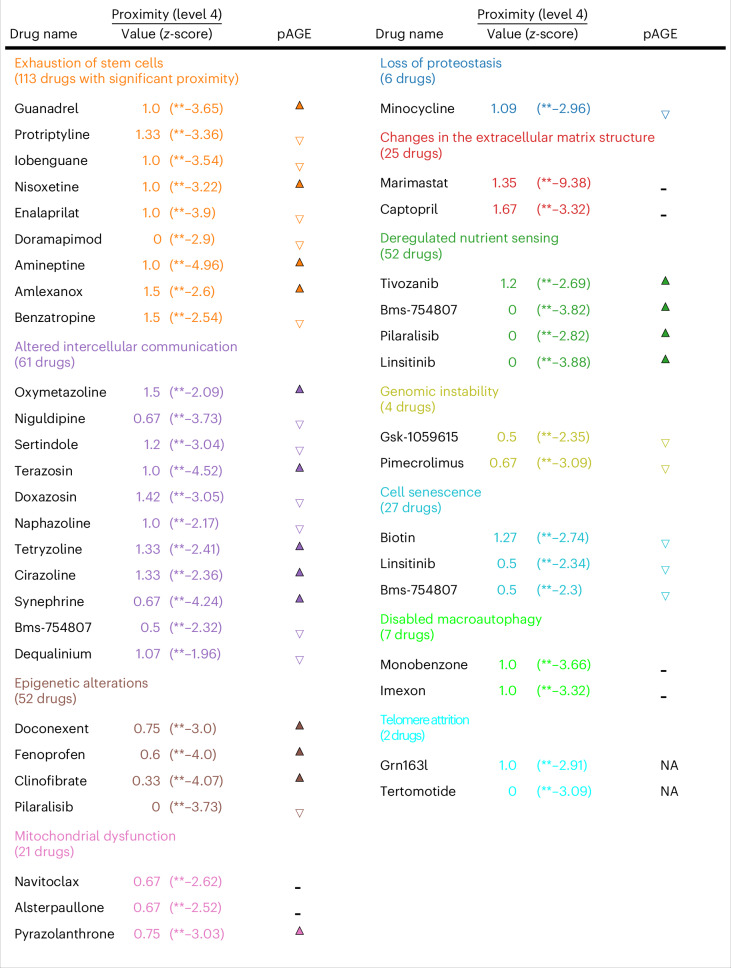
Drug repurposing for hallmark-targeted drugs. For each hallmark, top candidates (partial list) with statistically significant proximity to each of the hallmarks of aging are shown. The proximity value and *z*-score are shown for level 4, and pAGE directionality is based on level 2 or, if no high-confidence hallmark genes are present, for level 3. Positive pAGE values are shown as up and negative values as down. Two asterisks indicate statistically significant proximity (*z*-score < −1.96), calculated relative to a null distribution generated from random degree-matched drug targets and hallmark genes (Methods).

The network-based repurposing pipeline has allowed us to identify a total of 370 drugs (Fig. 7), each showing statistically significant proximity to one or more hallmarks of aging, hence potentially capable of modulating longevity. Of these, 60 drugs have CMap expression profiles, allowing us to calculate their pAGE parameter. Among these 60 drugs, 21 display positive pAGE, indicating that they represent pro-longevity drugs, and another 23 display negative pAGE, representing potential age-accelerating compounds. The remaining 16 drugs show inconsistent pAGE values for confidence levels 1 and 2; thus, we need further data to evaluate their impact on longevity. Below, we summarize the identified candidate drugs in the context of each hallmark.

### Exhaustion of stem cells

We identified 113 drugs with significant proximity (*z*-score < −1.96) to this hallmark at confidence levels 3–5, 19 of which have CMap data. Nine of 19 exhibit positive pAGE for level 3 (guanadrel, guanethidine, olopatadine, epirubicin, nisoxetine, clorgiline, amineptine, acemetacin and amlexanox), and 10 are predicted to be age-accelerating compounds (protriptyline, iobenguane, rescinnamine, ketorolac, enalaprilat, doramapimod, ph-797804, navitoclax, neomycin and benzatropine).

### Altered intercellular communication

Sixty-one drugs are significantly proximal to this hallmark across all five confidence levels, of which 25 have CMap data. Five exhibit positive pAGE for levels 1 and 2 (oxymetazoline, terazosin, tetryzoline, cirazoline and synephrine), and 11 are age-accelerating compounds (niguldipine, phentolamine, sertindole, prazosin, doxazosin, naphazoline, methoxamine, alfuzosin, bms-754807, thioproperazine and dequalinium).

### Epigenetic alterations

Of 52 drugs with significant proximity across all five confidence levels, only 5 have CMap data. Of these, doconexent, fenoprofen and clinofibrate have positive pAGE for levels 1 and 2, while pilaralisib is predicted to be an age-accelerating compound.

### Mitochondrial dysfunction

Twenty-one drugs exhibit significant proximity across all five confidence levels, but none have CMap data. If we also consider drugs that show proximity for four of five confidence levels, we find three drugs with CMap data (navitoclax, alsterpaullone and pyrazolanthrone), which have non-negative pAGE for levels 1 and 2.

### Loss of proteostasis

Six drugs have significant proximity at all five confidence levels, but we do not have CMap data for any of them. Considering those display proximity at four of five confidence levels, we find minocycline with negative pAGE.

### Changes in the extracellular matrix structure

As no genes from the OpenGenes database have confidence levels 1 or 2 for this hallmark, we examined levels 3–5, identifying 25 significantly proximal drugs. Among these, marimastat and captopril have CMap data and non-negative pAGE for level 3.

### Deregulated nutrient sensing

Fifty-two drugs reach significance (*z*-score < −1.96) across all five confidence levels, four of which have CMap data and positive pAGE for levels 1 and 2: tivozanib, bms-754807, pilaralisib and linsitinib.

### Genomic instability

Four drugs achieve significant proximity at all five confidence levels, yet none have CMap data. Adding those significant at four levels yields three drugs with CMap data (gsk-1059615, paricalcitol and pimecrolimus), of which pimecrolimus is an age-accelerating compound exhibiting negative pAGE for levels 1 and 2.

### Cell senescence

Twenty-seven drugs are significant at all five levels; three of them—biotin, linsitinib and bms-754807—have CMap data, and show non-positive pAGE values for levels 1 and 2.

### Disabled macroautophagy

With no confidence level-1 genes available, we analyzed levels 2–5, identifying seven drugs that reach significance for all four. Two, monobenzone and imexon, have CMap data with pAGE = 0 for level 2.

### Telomere attrition

Two drugs show significance across all five levels, although neither has CMap data.

To summarize, we find 370 drugs that exhibit significant proximity to at least one hallmark of aging. Of the 370 drugs, 60 have CMap data, enabling us to compute their pAGE; of these, 21 have a positive pAGE across all five confidence levels, making them prime candidates for experimental testing in animal models. We also find an additional 23 compounds with negative pAGE, indicative of potential age-accelerating effects. Literature evidence supporting our predictions is provided in Supplementary Section XV and Supplementary Table 13. The variability of pAGE across different cell lines and its impact on our final candidate selection is discussed in Supplementary Section IX.

We wish to emphasize that 310 of our drug-repurposing predictions currently lack CMap data. One can rely, therefore, on expression profiling to determine their pAGE value and assess their directionality. Extrapolating from our previous data, we anticipate that 35% (or about 108 drugs) of these candidates may benefit longevity (Methods). Interestingly, 83 of the 370 candidates identified by our pipeline are ‘network drugs’, representing drugs that do not target aging genes directly (Supplementary Section XVI). These candidates would be missed by approaches that do not consider the full network topology and the respective hallmark modules, consistent with a previous study^24^, which found that 76 of 77 drugs experimentally effective against COVID-19 could not be explained by direct interactions with viral target proteins.

## Proximity and pAGE predict therapeutic effects

**Fig. 8:**
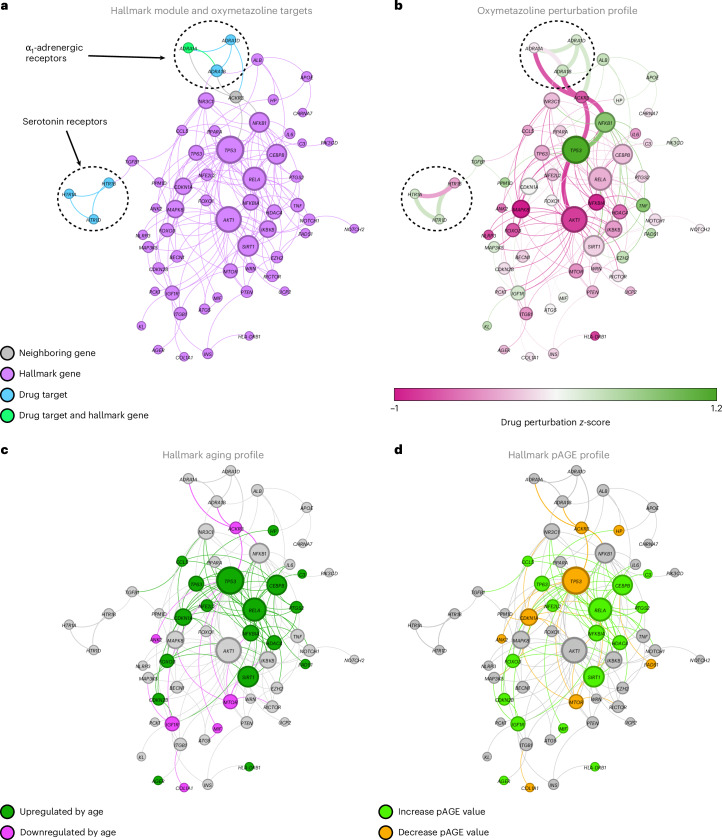
Proximity and pAGE predict mechanism of action. **a**, Oxymetazoline is one of the candidates for drug repurposing for the ‘altered intercellular communication’ hallmark (shown in purple). Oxymetazoline targets the proteins ADRA1A, ADRA1B and ADRA1D as part of the α_1_-adrenergic receptor protein group, and the proteins HTR1A, HTR1B and HTR1D as part of the serotonin receptor protein group. The α_1_-adrenergic receptor protein group directly connects to the module by the *ALB* and *NR3C1* genes, while the serotonin receptor protein group directly connects to the module by the *TGFB1* gene. The gene *ADRA1A* is both a drug target and a hallmark gene (green), while *ADRA1B* and *HTR1B* (blue) are nearest neighbors of the module, leading to a statistically significant proximity of 1.5 (Methods). **b**, Perturbing the MCF7 cell line with oxymetazoline, the drug signature upregulates (green) and downregulates (red) genes in the module. The color bar shows the *z*-score of the perturbation signature for each gene. The perturbation follows a detour path starting from the α_1_-adrenergic receptor protein group, and does not follow the shortest path to the module through the immediate target neighbors *ALB* and *NR3C1*. Instead, the target’s neighbor *ACKR3* (not a hallmark gene) is perturbed and transmits the information to the module. **c**, The aging signature marks genes that are upregulated (green) and downregulated (red) with age. **d**, The pAGE value is measured according to equation (3). By comparing the aging signature and the drug signature, genes with opposite signs (green) increase the pAGE value, while genes with similar signs (orange) decrease it, resulting in a statistically significant pAGE = 0.46.

The integrated network-based pipeline, augmented by the pAGE metric, is not only capable of identifying promising drug-repurposing candidates, but can also yield falsifiable predictions regarding a drug’s mechanism of action. We demonstrate this using oxymetazoline (see other candidates in Supplementary Section XI), a repurposing candidate predicted to impact the Altered Intercellular Communication hallmark (Fig. 7). Oxymetazoline is an α_1_- and α_2_-adrenergic agonist used clinically for nasal congestion, ocular allergic reactions and rosacea^48^. It targets the proteins ADRA1A, ADRA1B and ADRA1D, members of the α_1_-adrenergic receptor group, and HTR1A, HTR1B and HTR1D, members of the serotonin receptor group (Fig. 8a). Among these, ADRA1A is itself a hallmark gene (confidence level 1), and constitutively active ADRA1A has been linked to lifespan extension in mice^49^. Perturbing ADRA1A may therefore influence insulin signaling, AMPK–TOR pathways and chronic inflammation^50^.

To understand how oxymetazoline perturbs the hallmark module, we examined the propagation of its transcriptional perturbation signature through the interactome. The α_1_-adrenergic receptor group connects to the module through ALB and NR3C1, while the serotonin receptor group connects through TGFB1. However, these direct neighbors show relatively weak perturbation signatures, suggesting that the perturbation instead propagates through ACKR3, a non-hallmark protein with one of the strongest perturbation scores among the targets’ neighbors (Fig. 8b). ACKR3 interacts with hallmark proteins including NFKB1, TP53 and AKT1, each displaying significant perturbation signatures. Although expression of the α_1_-adrenergic receptor genes themselves does not significantly change with age (Fig. 8c), targeting them induces downstream transcriptional changes across the hallmark module, resulting in a significant pAGE = 0.46 (*z*-score = 2.35; Supplementary Section VIII). Specifically, oxymetazoline counteracts aging-associated expression changes in genes involved in sterile inflammation, including *CCL5*, *NFE2L2*, *AGER*, *RELA*, *CEBPB*, *C3*, *MIF*, *NFKBIA*, *HDAC4*, *PTGS2* and *HLA-DRB1*, as well as genes involved in impaired intercellular communication, including *CCL5*, *TP63*, *NFE2L2*, *FOXO3*, *CDKN2B*, *IGF1R* and *SIRT1* (Fig. 8b–d), thereby increasing the pAGE value (Fig. 8d).

In summary, integrating the hallmark network structure (Fig. 8a), drug perturbation profile (Fig. 8b) and aging-associated expression changes (Fig. 8c) reveals the molecular mechanisms through which a repurposable drug may modulate a hallmark module. The predicted mechanisms can then be validated in cell-based assays^34,35^ and animal models. While oxymetazoline serves here as an illustrative example, integrating pAGE with hallmark network structure (Fig. 3) enables similarly detailed mechanistic predictions for all candidates in Fig. 7 (Supplementary Section XI).

## Discussion

An important paradigm in aging research is the distinction between the ‘why’ of aging, represented by causal factors^51–53^ and the ‘how’ of aging, as encapsulated by ‘aging hallmarks’^54^. The longevity module introduced here reveals that from a network perspective, both the causal factors and the ‘hallmarks’ of aging are located in the same network neighborhood. This implies that therapeutic interventions designed to target either the drivers of aging or its hallmark processes must ultimately focus on the same well-localized neighborhood of the subcellular network, defined by the longevity module (Fig. 3). Consequently, from a network perspective, the traditional distinction between targeting ‘theories’ or ‘hallmarks’ may be less critical, given the realization that both involve the same network neighborhood.

Ultimately, our findings underscore the potential of leveraging the extensive hallmark-associated genetic evidence to identify drug-repurposing candidates for healthy longevity. Although the evidence presented here is primarily computational, it is supported by genetics and expression-based evidence, offering a principled basis for subsequent in vitro and in vivo validations, culminating in animal studies and eventually clinical trials.

We also introduce a key methodological advance, the pAGE metric, that helps us gauge whether a drug reinforces or counteracts aging-related transcriptional changes. Consequently, our pipeline uncovers both compounds that can act as pro-longevity compounds, as well as compounds that likely serve as ‘age-accelerating’ agents. These are also valuable for validating the genetic nexus of aging, illuminating further molecular targets, and identifying previously unknown side effects of existing drugs, helping clinicians to avoid unintended adverse effects on lifespan.

Because aging is multifactorial, effective interventions will likely require combination therapies targeting multiple hallmarks simultaneously. Notably, our approach identifies compounds that perturb several hallmarks, providing a framework for multi-target strategies (Supplementary Tables 5 and 6). Some candidates, such as bms-754807 (Fig. 7), affect multiple hallmark modules, consistent with our independent experimental study showing that lifespan-extending drugs in mice tend to target multiple hallmarks (Supplementary Table 12). Future work should therefore explore rational drug combinations using network-based models that account for drug–drug interactions, synergy and toxicity^55^.

Our work builds upon previous aging drug-repurposing approaches^56–58^ by integrating network topology with perturbational gene expression signatures. Prior approaches relied either on overlap between drug targets and aging genes^56^ or on CMap expression signatures alone^57,58^, lacking a network-based framework capable of identifying mechanism of action. In contrast, our approach captures broader network effects, including ‘network drugs’ that perturb hallmark modules without directly targeting hallmark genes, accounting for 83 of the 370 candidates identified by our pipeline (Supplementary Section XVI).

Our approach has several limitations. Aging processes vary substantially across tissues and cell types^59^, motivating integration of tissue-specific and single-cell data to refine hallmark definitions and tissue-level pAGE estimates. Tissue-dependent interactomes could be generated using GTEx data^60^, enabling evaluation of drug perturbations in biologically relevant cellular contexts^59^. In this study, we primarily relied on MCF7 perturbation profiles because of their broad CMap coverage. However, analyses in the noncancerous WI38 and IMR90 cell lines showed that most pAGE signatures exhibit only minimal variation (Supplementary Section IX).

Regarding our second key advance, the pAGE parameter, we note that it does not yet account for dosage, nonlinear responses or the possibility that a single compound may confer beneficial effects in one tissue and detrimental effects in another. Future work could help improve its predictive value by implementing these features.

Capturing the progression of hallmark modules at different chronological ages in humans or model organisms could also reveal critical windows when interventions are most effective, guiding stage-specific therapeutics for healthy aging. Finally, combining patient stratification (for example, genetic background, lifestyle factors) with network-derived hallmark signatures might help tailor interventions to individual aging trajectories, moving the field toward personalized antiaging strategies and opening the doors for Precision Geroscience.

## Methods

### Ethical regulations statement

No approval or ethical regulations were required for this study.

### The human interactome

The human interactome was assembled from 26 databases encompassing six different types of PPIs: (i) binary PPIs systematically tested by high-throughput yeast two-hybrid (Y2H) experiments (HI-Union^61^, HINT^62^, HIPPIE^63^); (ii) kinase–substrate interactions from both high-throughput and literature-curated low-throughput studies (KinomeNetworkX^64^, Human Protein Resource Database (HPRD)^65^, PhosphoSitePlus^66^); (iii) literature-curated PPIs identified by affinity purification–mass spectrometry (AP–MS) and low-throughput experiments (InWeb^67^, BioGRID^68^, PINA^69^, MINT^70^, IntAct^70^, InnateDB^71^, APID^72^, DIP^73^, LitBM17^61^); (iv) structure-derived PPIs supported by three-dimensional protein structures or cofractionation evidence (Instruct^74^, Interactome3D^75^, INSIDER^76^, CoFrac^77^); (v) signaling and regulatory interactions curated from literature and high-throughput studies (SignaLink2.0^78^, ENCODE^79^, RAIN^80^, RISE^81^); and (vi) protein complexes identified through systematic AP–MS experiments or computational inference (BioPlex2.0^82^, QUBIC^83^, lncRNome^84^, NPInter^85^). The genes were mapped to their Entrez ID based on the National Center for Biotechnology Information (NCBI) database as well as their official gene symbols. The resulting interactome includes 524,156 PPIs connecting 18,223 unique proteins. For comparison, the STRING PPI network^86^ contains 18,506 proteins and 728,844 interactions with a similar degree distribution (Supplementary Fig. 19). Robustness validation using the restricted unbiased PPI and the STRING PPI network is provided in Supplementary Section XII.

### LCC statistical significance

A set of genes *A* with a degree distribution ${P}_{A}(k)\,$ forms the LCC ${\rm{LCC}}(A)\in A\,$ in the interactome. The degree distribution of the interactome ${P}_{G}(k)$ is sampled using log-binning with a bin size of 100 nodes. The statistical significance of LCC(*A*) is measured by random sampling 1,000 gene sets in the interactome with size ${|A|}\,$ and degree distribution ${P}_{A}(k)$, and measuring the expected distribution of the LCC sizes, resulting in a *z*-score for the LCC(*A*) statistical significance.

### Network-based separation

The network-based separation *S*(*A,B*) between pairs of genes set *A* and *B* is calculated using the separation measurement introduced in ref. ^18^ as shown in equation (1):

1 $$S\left(A,B\right)=\left\langle {d}_{A,B}\right\rangle -\frac{\left\langle {d}_{A,A}\right\rangle +\left\langle {d}_{B,B}\right\rangle \,}{2}$$

where 〈*d*_*A,B*_〉 is the average shortest path between proteins in different gene sets and 〈*d*_*A,A*_〉 and 〈*d*_*B,B*_〉 are the average shortest path between proteins within the same gene set.

### Network-based proximity

The network-based proximity *P*(*S,T*) between pairs of genes set *S* and *T* (pair of hallmarks of aging, or a hallmark of aging a drug targets) is calculated using the proximity measurement introduced in ref. ^22^ as shown in equation (2):

2 $$P\left(S,T\right)=\,\frac{1}{|\left|T\right||}\mathop{\sum }\limits_{t\in T}\mathop{\min }\limits_{s\in S}d(s,t)$$

where *d*(*S,T*) is the shortest path length between nodes *s* and *t* in the network. The statistical significance of the proximity is obtained by comparing *P*(*S*,*T*) to the distribution of proximity values of 1,000 random selections of two sets of genes with size and degree distribution similar to *S* and *T*.

### Analysis of gene expression and perturbation parameters

The perturbation signatures of genes in the hallmark modules are retrieved from the CMap database (https://clue.io/) for the MCF7 cell line after treatment with all drugs in the CMap database. These signatures reflect the perturbation of the gene expression profile in the hallmark modules caused by the treatment with that particular drug relative to a reference population, which comprises all other treatments in the same experimental plate^87^. For drugs having more than one experimental instance (such as time of exposure, cell line and dose), we first select the highest dose to ensure a sufficiently strong perturbational signal, and then choose the instance with the highest distil_cc_q75 value (75th quantile of pairwise Spearman correlations in landmark genes; https://clue.io/connectopedia/glossary/).

### pAGE metric

The network medicine framework relies on undirected protein interactions, which capture a drug’s ability to perturb disease module, but lack information about the direction of the induced change, or whether a drug-induced perturbation is beneficial or detrimental for the studied phenotype. To overcome this limitation, we introduce the pAGE metric, which quantifies whether a drug’s impact on gene expression reinforces or counteracts documented age-related shifts. The metric is defined in three steps: (i) We define the longevity vector $\Gamma ={\left\{{\sigma }_{g}\right\}}_{g\in \Lambda }$, which encodes the age-induced expression change of 2,025 genes, where *σ*_*g*_ = +1 for 995 genes that are upregulated with age according to the OpenGenes database and *σ*_*g*_ = −1 for the 1,030 genes that are downregulated with age. (ii) We define a drug’s perturbation signature $\Theta ={\left\{{\sigma }_{g}^{{\prime} }\right\}}_{g\in \Lambda }$, for a set of genes $\Lambda$, capturing each gene’s changes in expression following exposure to the drug according to the CMap^87^. Specifically, ${\sigma }_{g}^{{\prime} }\in \left\{-\mathrm{1,0},+1\right\}$ in $\Theta$ indicates whether drug exposure decreases (${\sigma }_{{g}^{{\prime} }}=\,-1$), leaves unchanged (${\sigma }_{{g}^{{\prime} }}=\,0$) or increases (${\sigma }_{{g}^{{\prime} }}=\,+1$) expression of gene $g$. (iii) Finally, to assess the alignment between a drug’s perturbation profile $\Gamma$ and the longevity vector $\Theta$, we introduce the pAGE measure, defined as in equation (3):

3 $${\rm{pAGE}}=\,-\frac{1}{|{\Lambda }_{\varnothing }|}{\sum }_{i\in \Lambda }{\sigma }_{i}{\sigma }_{i}^{{\prime} },$$

where ${\Lambda }_{\varnothing }=\left\{{i|}{\sigma }_{i}{\sigma }_{i}^{{\prime} }\ne 0\right\}$ is a normalization factor that constrains pAGE to vary between −1 and +1. Whenever a drug’s induced expression changes match an age-related directional shift (${\sigma }_{i}$ and ${\sigma }_{i}^{\prime}$ share the same sign), the term ${\sigma }_{i}{\sigma }_{i}^{\prime}$ is positive, pushing pAGE toward negative values. Conversely, if the drug induces downregulation of a gene that is typically upregulated with age, it turns ${\sigma }_{i}{\sigma }_{i}^{\prime}$ negative, increasing pAGE. Thus, pAGE > 0 indicates that a drug attenuates age-related expression changes (pro-longevity compound), whereas pAGE < 0 implies that it may exacerbate them (age-accelerating compound). The statistical significance of the pAGE value is evaluated by comparing it to a control group of random drug signatures (Supplementary Section VIII).

### Aging-associated genes and gene expression signatures

The aging genes associated with each hallmark of aging were obtained from the OpenGenes database (https://open-genes.com/download/). Details of the curation process are provided in ref. ^25^, on the OpenGenes website (https://open-genes.com/about/) and in Supplementary Section I. To construct the longevity vector and compute pAGE (equation (3)), we used age-associated gene expression signatures retrieved from OpenGenes, which manually curates data from studies on the genetics of aging and longevity. These studies include: (i) gene expression changes (mRNA or protein levels increasing or decreasing with age), (ii) protein activity changes (alterations in functional activity, activation or inhibition, enzymatic activity or binding affinity) and (iii) protein modifications and localization (posttranslational modifications, changes in subcellular localization or secretion levels). Our analysis incorporated studies across all tissues and cell types, requiring the data to be derived from human samples and that the reported signatures be statistically significant (*P*value < 0.01). Tissue-specific analyses can be performed by filtering the interactome to include only proteins expressed in the tissue of interest^88^.

### Gene–disease associations

By surveying over 120 databases with gene–disease associations, we selected those that (i) were not compiled from other data sources, and (ii) provided at least one kind of evidence type classified as strong (functional evidence using an experimental essay), weak (genome-wide association study (GWAS) evidence but no experimental validation), inferred (relying on bioinformatics or single-nucleotide polymorphisms from imputation in GWAS) or not compatible ((I)ncRNA, miRNA and other transcripts with or without experimental validation). For each database, we kept the disease name, HGNC-converted gene names (HUGO Gene Nomenclature Committee) and evidence level. Finally, we combined the following data sources: GWAS from ClinGen, ClinVar, CTD, Disease Enhancer, DisGeNET, GWAS Catalog, HMDD45, lncBook, LncRNA disease, LOVD, Monarch, OMIM, Orphanet, PheGenI and PsyGeNet. All types of association were used in this study.

### Statistics and reproducibility

No statistical method was used to predetermine sample size. No data were excluded from the analyses. The statistical method used for each mesurment is described in the relevant section in Methods and in Supplementary Information.

### Reporting summary

Further information on research design is available in the Nature Portfolio Reporting Summary linked to this article.

## Supplementary information

Supplementary Information Supplementary Sections I–XVIII, Figs. 1–22 and Tables 1–14.

Reporting Summary

## Data Availability

All data supporting the findings of this study are available on GitHub (https://github.com/BnayaGross/Longevity-module/) and within the paper and its Supplementary Information.

## Code availability

The code used to produce the results and figures in the study is published on GitHub (https://github.com/BnayaGross/Longevity-module/). An open-source Python package is available at https://github.com/BnayaGross/sharp-aging/.
