# Supplementary material for: Network-driven discovery of repurposable drugs targeting hallmarks of aging
Source: Nat Aging. 2026 Jun 26;6(7):1516–31. doi: 10.1038/s43587-026-01161-8 (PMC13375550; doi:10.1038/s43587-026-01161-8)
Supplement: Supplementary file 1 — Supplementary Sections I–XVIII, Figs. 1–22 and Tables 1–14. [file 43587_2026_1161_MOESM1_ESM.pdf]

nature aging

Supplementary information https://doi.org/10.1038/s43587-026-01161-8

Network-driven discovery of repurposable drugs targeting hallmarks of aging

In the format provided by the authors and unedited

Contents

SI.I The OpenGenes database confidence level system 2

SI.II Validation of the aging genes 3

SI.III Classifying unclassified genes using network topology 5

SI.IV Network properties of aging genes 7

SI.V Separation and proximity of the hallmark modules 7

SI.VI The core of the longevity module 8

SI.VII Proximity predicts perturbation impact 9

SI.VIII $pAGE$ statistical significance 10

SI.IX $pAGE$ variability across cell lines 14

SI.X Drug predictions with marginal significance 14

SI.XI Mechanism of action of drug candidates 16

SI.XII Robustness validation with an unbiased PPI, STRING interactome, and edge permutation 18

SI.XIII Circularity Check of ITP drug validation 19

SI.XIV Experimental Setup for Mouse Lifespan and Healthspan Assays 20

SI.XV Literature Evidence for the Pipeline's Final Candidates 22

SI.XVI Network drugs 24

SI.XVII Baseline ablation analysis 25

SI.XVIIILeave-out analysis 25

SI.I The OpenGenes database confidence level system

The OpenGenes database [1] relies on manually curated data sourced from diverse experiments, capturing relationships between individual genes and aging. Curators then reviewed the experimental evidence, including lifespan interventions, gene knockouts, overexpression studies, and human population associations. Each entry records detailed metadata such as organism, experimental design, lifespan outcomes, and tissue specificity. Importantly, it does not rely on pathway knowledge. OpenGenes uses a strict confidence-level system to classify aging-related genes, evaluating 11 distinct aging criteria to assign each gene to one of five confidence groups:

Group A:
- Changes in gene activity extend mammalian lifespan

Group B:
- Changes in gene activity extend non-mammalian lifespan

Group C:
- Association of genetic variants and gene expression levels with longevity

Group D:
- Association of the gene with accelerated aging in humans
- Changes in gene activity reduce mammalian lifespan
- Changes in gene activity reduce non-mammalian lifespan
- Changes in gene activity protect against age-related impairment
- Age-related changes in gene expression, methylation or protein activity in humans

Group E:
- Age-related changes in gene expression, methylation or protein activity in non-mammals
- Changes in gene activity enhance age-related deterioration

- Regulation of genes associated with aging

Based on the above groups the confidence level of a gene is determined:

Highest (26 genes): $A + C$: A gene meets selection criteria in both groups A and C

High (52 genes): $A - C$: A gene meets selection criteria in group A but not group C

Moderate (88 genes): B: A gene meets selection criteria in group B

Low (120 genes): $C, D \geq 2$: A gene meets at least two selection criteria in groups C or D

Lowest (2072 genes): $C, D = 1$ or E: A gene meets no more than a single selection criterion in groups C or D or a selection criteria in group E

SI.II Validation of the aging genes

The OpenGenes database provides substantial individual evidence for each aging gene. To further strengthen confidence in the gene pool provided by OpenGenes, we conducted a series of validation tests. We first measured the enrichment of the aging genes in six independent, aging-related high-throughput studies: three GWAS studies [2, 3, 4], the GTEx study [5], the transcriptional landscape (TL) of age in human peripheral blood gene expression meta-analysis [6], and the Aging Atlas database [7] (see Table S1). We checked that these studies are not included among the 2,701 unique DOIs in OpenGenes and thus provide external and orthogonal validation from large-scale, independent datasets, supporting the age-relevance of the OpenGenes gene set. We hypothesized that genes identified in these studies would significantly overlap with multiple hallmark genes. Consistent with this hypothesis, each GWAS study shows statistically significant overlap (p-value < 0.05) with at least five different hallmarks. The GTEx study shows enrichment only for

the Mitochondrial Dysfunction and Loss of Proteostasis hallmarks. The TL study shows enrichment with all hallmarks except Changes in Extracellular Matrix Structure and Telomere Attrition. Finally, the Aging Atlas database shows enrichment across all hallmarks.

As a second validation, we measured the overlap of the aging gene list with the genes associated with 5 aging-related diseases. We find that genes associated with Stroke and Coronary artery disease show enrichment with 4 different hallmarks, genes involved in Diabetes mellitus type 2 show enrichment with 5 different hallmarks, Alzheimer's disease genes with 3 hallmarks, and Pulmonary disease chronic obstructive genes with 2 hallmarks (Table. S2). Finally, the onset rate of many cancers increases with age. We therefore measured the enrichment of the aging genes with 8 different types of cancer: breast neoplasms, carcinoma non-small cell lung, lung neoplasms, prostatic neoplasms, ovarian neoplasms, skin neoplasms, stomach neoplasms, and colonic neoplasms. As shown in Fig. S4, the hallmarks Epigenetic alterations and Mitochondrial dysfunction are enriched with all 8 types of cancer genes while the other hallmarks are enriched with some of the cancer types. Finally, we performed KEGG pathway analysis [8], finding that genes associated with the hallmarks of aging are enriched in longevity-related pathways, type 2 diabetes mellitus pathways, Cellular senescence pathways, and multiple cancer pathways (Fig. S11).

Furthermore, we collected 143 genes associated with DNA repair [9] and measured their enrichment with the genes of the hallmarks of aging. As expected, the Genomic instability hallmark shows the highest enrichment (see Methods), however, an additional 5 out of the 11 hallmarks of aging are also enriched (p-value < 0.01, Fig. S5), showing the common genetic origin of both DNA repair mechanism and the hallmarks of aging.

DNA damage is the main source of Progeroid syndromes [10, 11] also known as premature

aging syndrome [12] or accelerated aging [13]. While Progeroid syndromes are defined by their genetic origin, there is a strong overlap between their phenotype and the hallmark of aging [14]. Hence, we hypothesize that the genes associated with Progeroid syndroms should show a strong overlap with the pool of aging genes. To test this hypothesis we collected 40 genes associated with 20 different Progeroid syndromes [14], finding that the genes associated with the DNA repair hallmark are significantly enriched with the Progeroid syndromes genes (p-value $= 3.52 \times 10^{-52}$), and also finding significant overlap with 5 out of the 11 hallmarks of aging (Fig. S5).

SI.III Classifying unclassified genes using network topology

Among the 2,358 aging genes, 1,108 can not be associated with a specific hallmark of aging according to our current knowledge of their biological function. While further study of their function may unveil such associations, we can use the topology of the human interactome to estimate these associations. Once the unclassified genes are associated with a specific hallmarks of aging, they can be used in the drug-repurposing pipeline.

Random walk with random resetting.– Random Walk with Restart (RWR) is a network propagation algorithm commonly used to identify gene associations [15]. Other algorithms in this class include information diffusion and electrical-resistance-based methods [16]. Here, we used each set of hallmark genes as a seed for the random walk. For each hallmark, we calculated the probability of visiting every unclassified gene. For each unclassified gene, we then compared the visit probabilities it received from each hallmark and normalized these probabilities by the number of genes within that hallmark, yielding

a set of scores that quantify the association of each unclassified gene with each hallmark. Each unclassified gene was subsequently assigned to the hallmark with the highest score (i.e., the one that most frequently visited it). The resulting hallmark assignments for the unclassified genes are shown in Fig. S3**(a)**.

The ROBUST algorithm.– Over the past decade, more advanced algorithms have been developed to identify gene associations based on network topology. Examples include DOMINO [17], MuST [18], and DIAMOnD [19]. More recently, the ROBUST algorithm was introduced [20], which outperforms DOMINO, MuST, and DIAMOnD by explicitly correcting for study bias in protein–protein interaction networks and using a bias-aware Steiner tree approach. Unlike methods that rely on local connectivity or heuristic clustering, ROBUST considers the global network structure and allows parameter tuning to balance seed coverage and module compactness, making it robust and biologically meaningful for small seed sets or partially annotated gene lists. We applied the ROBUST algorithm to identify hallmark associations for previously unclassified aging genes. For each hallmark, known hallmark genes were used as seeds in ROBUST to expand the module and incorporate isolated aging genes. The newly expanded modules now include 51 of the unclassified genes, and the number of newly classified genes per hallmark is shown in Fig. S3**(b)**. In contrast to the RWR algorithm, which eventually assigns all unclassified aging genes to different hallmarks, ROBUST associates only 51 genes, but with higher confidence in their biological relevance to the respective hallmark.

SI.IV Network properties of aging genes

Betweeness and median degree

Proteins interacting with many other proteins were shown to be more likely to play important roles in physiological processes [21]. We find that the genes implicated in aging also fit this pattern, having a 7 times higher median degree (Fig. S6**a**) and an order of magnitude higher median betweenness (Fig. S6**b**) compared to genes not involved in aging.

Jaccard index

The pairwise comparisons using the Jaccard index [22] reveal gene overlap among 47 of the 55 hallmark pairs (Fig. S7). The strongest overlap is between Genomic instability and Telomere attrition (16 overlapping genes, Jaccard Index = 0.31), emphasizing the central role of genomic stability in maintaining telomere function [23]. Likewise, Cell senescence displays a high Jaccard index with both Genomic instability (13 overlapping genes, Jaccard Index = 0.2), and Telomere attrition (5 overlapping genes, Jaccard Index = 0.13), in line with their established role in driving senescence [24].

SI.V Separation and proximity of the hallmark modules

The separation metric, S_{AB}, quantifies the extent of network overlap between modules A and B, such that $S_{AB} < 0$ denotes overlapping modules and $S_{AB} > 0$ indicates topologically distinct modules (Fig. 2**a**). Pairwise calculations of S_{AB} revealed that ten of the eleven modules overlap ($S_{AB} < 0$), and that only one hallmark, Changes in the extracellular matrix structure, displays a positive separation value, hence begin separated from the others (Fig. S9).

The proximity measure, P_{AB}, measures the shortest paths among genes in modules A and B. Remarkably, all hallmark pairs showed statistically significant proximity (z-score < -1.96), including the previously separated hallmark of Changes in the extracellular matrix structure. In other words, while it does not overlap with other modules in network space, this hallmark lies in the immediate neighborhood of two other hallmark modules (Fig. S8).

SI.VI The core of the longevity module

While the overlap of many pairs of hallmarks indicates a common genetic origin, it is yet unclear if it is a result of a genetic core shared by all the hallmarks of aging or if each pair of hallmarks is overlapped separately. For example, three hallmark modules can overlap without having a common genetic origin where each pair overlapping in a separated network neighborhood or the overlap of all three hallmarks be in the same area (Fig. S10). To counter this problem we noticed that if each pair of modules overlaps separately, the *intersections* of gene sets of each pair will not overlap while if the overlap of the modules is in the same area, the gene sets intersections are expected to overlap (Fig. S10). Therefore, we measured the separation value of the intersections of each pair of hallmarks (Fig. S10) and found that most of them overlap. This indicates that not only are the hallmarks of aging located in the same network neighborhood and form the longevity module, but the longevity module itself has a core where all the hallmarks of aging overlap. We hypothesize that the genes in the core of the longevity module have a more central role in aging. An obvious example is TP53 associated with 7 hallmarks, its role in aging being rooted in DNA damage response [25], a process central to many aging processes. The gene FOXO1, involved in 6 hallmarks, is also known to play multiple cellular roles

including insulin signaling in metabolic pathways, and is also highly associated with aging [26]. SIRT1 (6 hallmarks) has a strong relation to multiple aging-related functions [27, 28]. AKT1 (5 hallmarks) is associated with osteosarcopenia and impact on mice lifespan [29]. The enrichment of the longevity module with cancer and DNA repair genes (Fig. S4 and Fig. S5) results in cancer and DNA repair-associated genes such as the ATM gene [30, 31] (5 hallmarks) as an interconnected gene in the module. The PARP1 gene (5 hallmarks) plays a main role in neurodegenerative diseases [32] and longevity [33].

SI.VII Proximity predicts perturbation impact

We retrieved the perturbation signature of each drug in the MCF7 cell line from the Connectivity Map (CMap) database [34], allowing us to test if and to what degree the drug perturbs each hallmark module. We use two parameters to characterize the perturbation impact of a drug (see Fig. S1**b** and methods).

(i) *The perturbation magnitude* $\mathcal{M} \in [0, 1]$ measures the maximal perturbation score of genes in a specific hallmark, normalized by the maximal perturbation score in the whole network. We find that $\mathcal{M}$ increases with the perturbation dose (p-value = 1.91×10^{-10}, dose of $0.1\mu M$ compared to $10\mu M$ for the MCF7 cell line) but it is unaffected by the perturbation time (p-value = 0.44, perturbation time of $6h$ compared to $48h$ for the MCF7 cell line), see Fig. S12.

(ii) *Perturbation globality* $\mathcal{G} \in [0, 1]$, is the fraction of statistically significant (|z-score| $>$ 1.96) perturbed genes in the module. While $\mathcal{M}$ and $\mathcal{G}$ are correlated (Pearson correlation coefficient 0.65, Fig. S13) they offer different insights into the perturbation signature (Fig. S1**c,e**). For example, perospirone strongly perturbs a few genes in a module, hence we observe a high magnitude $\mathcal{M} \rightarrow 1$ along with a low globality $\mathcal{G} \rightarrow 0$. In contrast,

lestaurtinib perturbs most of the genes in the module just above the significance cutoff, resulting in high globality $\mathcal{G} \to 1$ but intermediate magnitude $\mathcal{M} \ll 1$ (Fig. S1**c**,**e**).

We can measure the perturbation parameters $(\mathcal{M}, \mathcal{G})$ for 9 out of the 17 drugs under clinical trials for humans that were tested in the CMap database. dasatinib, sirolimus, and mesalazine have significant magnitude ($\mathcal{M} > 0.2$) for all the hallmarks of aging while others (e.g. aspirin, acarbose, metformin) significantly modulate four of them, a characteristic which can be affected by the dose level (Fig. S12). Despite the correlation between $\mathcal{M}$ and $\mathcal{G}$, high globality is observed only for dasatinib and mesalazine and only for the Telomere attrition hallmark, indicating that longevity drugs tend to have a local effect on few genes and not the entire modules.

SI.VIII $pAGE$ statistical significance

The control group for calculating the statistical significance of $pAGE$ is a distribution of randomized drug signatures. Considering a random sequence of N variables $X_i \in \{-1, +1\}$. Each variable is characterized by mean $\mathbb{E}[X_i] = 0$ and variance $Var(X_i) = \mathbb{E}[X_i^2] - \mathbb{E}[X_i]^2 = 1$. The sum of the variables $S = \sum_{i=0}^{N} X_i$ is characterized by $\mathbb{E}[S] = \sum_{i=0}^{N} \mathbb{E}[X_i] = 0$ and $Var(S) = \sum_{i=0}^{N} Var(X_i) = N$. Following Eq. 3, $pAGE = S/N$. Hence, $\mathbb{E}[pAGE] = \mathbb{E}[S]/N = 0$ and $Var(pAGE) = Var(S)/N^2 = 1/N$. According to the Central Limit Theorem, the distribution of $pAGE$ will be approximately normally distributed for large N. the standard deviation $\sigma = 1/\sqrt{N}$ will be used to calculate the z-score of a $pAGE$ value with the threshold for significance will be |z-score| $= 1.96$ and |z-score| $= 1.645$ for marginal significance.

$pAGE$ statistical significance for clinical and ITP drugs

To assess the relevance of $pAGE$, we measure for 9 of the 17 drugs in clinical trials for aging or longevity for which CMap data are available. We find that all nine displayed positive $pAGE$ for at least three hallmarks (see Table S4). 6 drugs show positive and statistically (or marginally) significant $pAGE$ for at least one hallmark.

We also evaluated eight of the 11 ITP-confirmed lifespan-extending drugs [35] with CMap data. We find that all eight have positive $pAGE$ for at least three hallmarks (Table S3). 7 drugs show positive and statistically (or marginally) significant $pAGE$ for at least one hallmark. Our ability to systematically and confidently identify the direction of impact of a drug on a specific hallmark module, distinguishing potential therapeutic interventions from adverse effects, represents our second key result.

$pAGE$ statistical significance for drug-repurposing candidates

Considering the statistical significance for $pAGE$ we summarize below the results for the drug-repurposing candidates listed in the main manuscript.

Exhaustion of stem cells: We identified 113 drugs significantly proximal (z-score < -1.96) to this hallmark at confidence levels 3–5, 19 of which have CMap data. Four of these- guanadrel, nisoxetine, amineptine, and amlexanox exhibit positive $pAGE$ across all tested levels, of which guanadrel has statistically significant $pAGE$ for one of the levels. Our pipeline also identified 5 age-accelerating compounds, protriptyline, iobenguane, enalaprilat, doramapimod, and benzatropine, of which iobenguane has statistically significant $pAGE$ for one of the levels.

Altered intercellular communication: Sixty-one drugs are significantly proximal to this hallmark across all five confidence levels, of which 25 have CMap data. Seven exhibit positive

$pAGE$ across the board, including oxymetazoline, metaraminol, terazosin, tamsulosin, tetryzoline, cirazoline, and synephrine. Two of which-oxymetazoline and synephrine has statistically significant $pAGE$ for at least one of the levels. Our pipeline also identified 7 age-accelerating compounds, niguldipine sertindole, doxazosin, naphazoline, linsitinib, bms-754807, and dequalinium, of which all but linsitinib and dequalinium have statistically significant $pAGE$ for one of the levels.

Epigenetic alterations: Of 52 drugs with significant proximity across all five confidence levels, five have CMap data. Of these, only clinofibrate maintains positive $pAGE$ across all levels but none is statistically significant. Our pipeline also identified 1 age-accelerating compound - pilaralisib but without statistically significant $pAGE$ for any of the levels.

Mitochondrial dysfunction: Twenty-one drugs exhibit significant proximity across all five confidence levels, but none have CMap data. Considering also those hitting four out of five levels adds three drugs with Cmap data - navitoclax, alsterpaullone, and pyrazolanthrone. Among them, pyrazolanthrone maintains positive $pAGE$ across all levels with one level statistically significant.

Loss of proteostasis: Six drugs have significant proximity at all five confidence levels but we do not have CMap data for any of them. Considering those hitting four out of five levels add only minocycline with Cmap data, However, its $pAGE$ values are inconsistent in the different levels.

Changes in the extracellular matrix structure: As no genes from the OpenGenes database have confidence levels 1 or 2 for this hallmark, we examined levels 3–5, identifying 25 significantly proximal drugs. Among these, two drugs have CMap data. marimastat (an investigational cancer therapy) displays positive but not statistically significant $pAGE$. cap-

topril (an antihypertensive) displays negative but not statistically significant $pAGE$.

Deregulated nutrient sensing: Fifty-two drugs reach significance (z-score < -1.96) across all five confidence levels, four of which have CMap data. None show positive $pAGE$ at all five levels. Yet, Bms-754807, pilaralisib, and linsitinib exhibit positive $pAGE$ in four levels.

Genomic instability: Four drugs achieve significant proximity at all five confidence levels, yet none have CMap data. Adding those significant at four levels yields three more drugs with Cmap data-gsk-1059615, paricalcitol, and pimecrolimus have CMap data. Both gsk-1059615 and pimecrolimus exhibit negative $pAGE$ across all levels and are statistically significant only for gsk-1059615,

Cell senescence: Twenty-seven drugs are significant at all five levels; three of them-biotin, linsitinib and bms-754807 have CMap data, but show inconsistent $pAGE$ values.

Disabled macroautophagy: With no confidence level 1 genes available, we analyzed levels 2–5, identifying seven drugs that reach significance for all four. Two, monobenzone (a depigmenting agent) and imexon (an anticancer therapy), have CMap data and only imexon exhibits positive but statistically not significant $pAGE$.

Telomere attrition: Two drugs prove significant across all five levels, though neither has CMap data.

To summarize these results, we find 370 drugs that exhibit significant proximity to at least one hallmark of aging. Of these, only 60 drugs have CMap data, enabling us to compute $pAGE$; of these, 3 have a positive and statistically significant $pAGE$ across all five confidence levels, making them prime candidates for experimental testing in animal models. By contrast, we find 6 compounds with negative and statistically significant $pAGE$,

indicative of potential age-accelerating effects, and potentially serving as empirical tests of our framework should they induce aging phenotypes *in vivo*.

SI.IX $pAGE$ variability across cell lines

Drug signatures vary between cell lines. To characterize this variability and validate the robustness of the $pAGE$ metric, we collected the signatures of drugs of the lung cell lines WI38 and IMR90 (in addition to the MCF7 cell line presented in the main manuscript). Since the available data for these cell lines is limited in the CMap database, we extended our criteria to include drugs with significant proximity in few of the confidence levels (see Tables. S7, S8 and S9 for the MCF7, WI38 and IMR90 cell lines respectively). As shown in Fig. S16, the majority of $pAGE$ signatures show a very low change between cell lines. The cell lines WI38 and IMR90 show even less change, since both are lung cell lines. Note that small modules are sensitive to these changes and strongly fluctuate with ± 1 values.

SI.X Drug predictions with marginal significance

When extending our criteria to include marginal significance ($-1.96 <$ z-score < -1.645) we identify additional 206 drugs. Among these, 27 have Cmap data and 12 with positive $pAGE$ should also be considered as candidates.

Exhaustion of stem cells: The OpenGenes database contains no genes with confidence levels 1 and 2 for this hallmark, limiting measurements to levels 3-5. We find an additional 86 drugs with marginal significance, of which 11 drugs with CMap data. Four of these, lenalidomide, cyclizine, pseudoephedrine, and fostamatinib show positive $pAGE$ across all levels.

Altered intercellular communication: We find an additional 21 drugs with marginal significance, of which 7 drugs with CMap data. Four of these, clonidine dobutamine, isometheptene, and pizotifen show positive $pAGE$ across all levels.

Epigenetic alterations: We find an additional 7 drugs with marginal significance, of which only Aspirin has CMap data and shows positive $pAGE$ across all levels.

Mitochondrial dysfunction: We find an additional 16 drugs with marginal significance, of which only one has CMap data but shows negative $pAGE$.

Loss of proteostasis: We find an additional 5 drugs with marginal significance, of which only one has CMap data but shows negative $pAGE$.

Changes in the extracellular matrix structure: The OpenGenes database contains no genes with confidence levels 1 and 2 for this hallmark, limiting measurements to levels 3-5. We find an additional 8 drugs with marginal significance, none of them has CMap data.

Deregulated nutrient sensing: We find an additional 19 drugs with marginal significance, of which two drugs with CMap data and only staurosporine show positive $pAGE$ across all levels.

Genomic instability: We find an additional 2 drugs with marginal significance, of which only one has CMap data but shows negative $pAGE$.

Cell senescence: We find an additional 26 drugs with marginal significance, of which only one has CMap data but shows negative $pAGE$.

Disabled macroautophagy: No genes with confidence level 1 are available for this hallmark; hence, we use confidence levels 2-5. We find an additional 14 drugs with marginal significance, of which only navitoclax and hexylresorcinol with CMap data, and both show

positive $pAGE$.

Telomere attrition: We find an additional two drugs with marginal significance, none of them has CMap data.

SI.XI Mechanism of action of drug candidates

The integrated network-based pipeline, augmented by the $pAGE$ metric, is not only capable of identifying promising drug-repurposing candidates, but can also yield falsifiable predictions pertaining to the drug's mechanism of action. We demonstrate this on synephrine, a repurposing candidate that according to our pipeline impacts the Altered Intercellular Communication hallmark (Table. 1). synephrine is a sympathomimetic α_1-adrenoceptor agonist tested for Anesthesia therapy and Hypotension [36], that is also used as a dietary supplement for weight loss [37]. It binds to the α_1-adrenergic receptor protein group, targeting the proteins ADRA1A, ADRA1B, and ADRA1D (Fig. S17**a**). Of these, ADRA1A is a hallmark gene (confidence level 1), as mice expressing a constitutively active mutant ADRA1A lived significantly longer [38]. While the potential impact of synephrine on longevity is unknown, perturbing the activity of ADRA1A has the potential to extend lifespan by altering molecular mechanisms related to insulin signaling, the AMPK and TOR pathways, and chronic inflammation [39].

To understand how the perturbation induced by synephrine propagates through the hallmark module, we examined the gene perturbation signature in the vicinity of its targets ADRA1A, ADRA1B and ADRA1D. The ALB, NR3C1 and STAT1 proteins are direct interacting partners of synephrine's target ADRA1B. Yet, the drug-induced perturbation signatures of the hallmark proteins ALB and NR3C1 are weak (z-score $= -0.63$ for ALB and

z-score $= -0.24$ for NR3C1). This suggests that the drug induced perturbation propagates through the STAT1 protein, which has some of the highest perturbation scores of the targets' neighbors (z-score $= 0.97$, Fig. S17**b**). The STAT1 protein interacts with the hallmark proteins MTOR (z-score $= -1.01$), PPARA (z-score $= -0.96$), TGFB1 (z-score $= -1.54$) and FOXO3 (z-score $= -0.89$), each displaying significant perturbation signature. While the expression patterns of the α_1-adrenergic receptor proteins do not change with age (Fig. S17**c**), we predict that targeting them with synephrine leads to a perturbation signature that affects the expression patterns of multiple genes in the Altered intercellular communication hallmark module (Fig. S17**b**) resulting in a statistically significant $pAGE = 0.46$ (z-score $= 2.35$, see Supplementary Section SI.VII). Specifically, the expression pattern of genes CCL5, C3, CEBPB, PTGS2, NFE2L2, RELA, NFKBIA, MIF, AGER, and HLA-DRB1 that are involved in the aging mechanism of sterile inflammation, are perturbed by synephrine in the direction that corrects the aging-induced expression changes (Fig. S17**b,c**), increasing the $pAGE$ value (Fig. S17**d**). Similarly, the expression of CCL5, FADS1, FOXO3, NFE2L2, TP53, TP63, CDKN2B, CDKN1A, and IGF1R genes involved in the aging mechanism of intercellular communication impairment, are also perturbed by synephrine, opposing their aging-induced expression changes and further increasing the $pAGE$ value (Fig. S17**b-d**).

In summary, the integration of the network module (Fig. S17**a**), the drug's perturbation profile (Fig. S17**b**), and age-associated expression changes (Fig. S17**c**) unveils the molecular mechanism by which a repurposable drug is expected to modulate a hallmark module.

SI.XII Robustness validation with an unbiased PPI, STRING interactome, and edge permutation

The human interactome used in this study was assembled from 26 databases encompassing six types of protein–protein interactions (see Methods section in the main text). However, PPI networks are prone to research bias, and manual curation can introduce artificial modularity around well-studied genes. To ensure the robustness of our findings, we performed three validation checks (Table. S10):

(1) Unbiased PPI. We conducted a complementary analysis using a restricted, unbiased interactome derived from six high-throughput, experimentally generated datasets: the high-throughput Y2H screen (HI-Union), AP–MS-based protein complexes (BioPlex2.0), and structure-supported interactions (Instruct, Interactome3D, INSIDER, and CoFrac). In other words, we excluded the biased, investigator-driven small-scale interactions. The restricted interactome is smaller and less dense, comprising 205,051 protein–protein interactions connecting 16,352 unique proteins. We found that the statistical significance of hallmark modules remained largely consistent. Similar to the original interactome, 9 of 11 modules remained statistically significant. The module associated with Disabled macroautophagy became marginally significant, while the Loss of proteostasis module lost significance. This complementary analysis ensures that our conclusions are not driven by curation bias towards well-studied genes.

(2) STRING interactome. We repeated the analysis using the STRING database [40]. The STRING PPI network contains 18,506 proteins and 728,844 interactions. We found that all 11 hallmark modules reached statistical significance, providing strong evidence for the robustness of our findings.

(3) Edge permutation. We determined the statistical significance of the LCCs in the human interactome used in this study using edge permutations, while preserving the original degree distribution. We found that the statistical significance of the hallmark modules under node permutation remained largely consistent, with two exceptions: Loss of proteostasis became significant, and Exhaustion of stem cells lost significance. While in the main text we report significance results based on node permutation, these additional analyses confirm that our findings are largely robust to the choice of permutation scheme.

SI.XIII Circularity Check of ITP drug validation

While the OpenGenes database includes studies reporting interventions that alter gene activity and affect lifespan in model organisms (Supplementary Section SI.I), there is a potential concern of circularity when validating our pipeline with the ITP drugs. OpenGenes contains 5,564 PubMed entries and 2,701 DOIs of aging-related studies, and none of the 20 ITP studies [35] are included. Although other studies linking these drugs to aging may exist in OpenGenes, this does not guarantee that SHARP will identify them. SHARP is a network-based framework that begins with 1,250 hallmark-associated genes and predicts drugs that modulate the corresponding disease modules; a drug is identified only if it shows proximity to the entire hallmark module. Furthermore, although 51 of the 235 targets of ITP and clinically tested drugs are present in OpenGenes, the computation of $pAGE$ does not rely on any of this information. OpenGenes aging signatures are manually curated from studies analyzing natural transcriptional changes during aging, rather than intervention studies. In contrast, the directional information in SHARP comes from CMap drug perturbation profiles, which are entirely independent of OpenGenes. Therefore, validating $pAGE$ using ITP and clinical drugs does not create a circular validation loop,

but instead provides an external test set for the aging signature.

SI.XIV Experimental Setup for Mouse Lifespan and Healthspan Assays

To evaluate SHARP on real-world longevity interventions, we used new experimental data obtained by the Gladyshev lab that tested ten candidate longevity compounds in aged mice [41]. This study was performed independently and in parallel to the current study, and its experimental findings were incorporated during the final stages of the present work, after the predictive work had concluded. The details of the screening of compounds for their association with transcriptomic signatures of longevity and experimental setup are described in [41] and are summarized below.

Screening of compounds for their association with transcriptomic signatures of longevity.– Compounds were prioritized by screening for perturbagens in the CMAP database that induce gene expression changes similar to established longevity-associated signatures. Query inputs included transcriptomic signatures from long-lived mammals and known lifespan-extending interventions in mice, spanning multiple organs. Compounds were ranked using aggregated longevity scores based on normalized enrichment across these signatures, which showed consistent and positively correlated predictions across datasets.

Experimental Animals.– The study used ~25-month-old *C57BL/6JN* mice, corresponding to late-life intervention conditions. Each treatment arm contained approximately ten males and ten females, maintained under standard specific-pathogen-free conditions. Animals were randomly assigned to treatment and control groups.

Compound Administration.– Each treatment group received a diet supplemented with the test compound, while age-matched control mice received the same base diet without supplementation. Dietary administration enabled long-term, low-stress dosing appropriate for late-life intervention studies.

Study Design and Endpoints.– Interventions were initiated at 25 months of age and maintained throughout the observation period. The study assessed two primary endpoints:

- **Lifespan**, measured by survival analysis of treated versus control mice.
- **Healthspan**, assessed by physiological, histological, metabolic, and molecular markers of aging reported in [41], including cardiometabolic function, hematologic parameters, tissue pathology, and organ-specific aging features.

Compounds were considered beneficial if they produced statistically significant improvements in either lifespan or any major healthspan metric, according to the evaluation criteria discussed in [41].

Summary of Experimental Findings.– We evaluated SHARP for the 10 compounds tested for lifespan and healthspan in mice. The experiments show that six of the ten compounds (Vorinostat, Selumetinib, LY-294002, KU-0063794, Celastrol and AZD-8055) extended lifespan or healthspan, while four (Valdecoxib, GDC-0941, NVP-BEZ235 and Ascorbyl-palmitate) did not. We find that the six successful compounds showed statistically significant (or marginal) proximity to at least one hallmark (Table S12). Five of these six had CMap data (Vorinostat, Selumetinib, LY-294002, KU-0063794, and AZD-8055), and all of them showed positive $pAGE$ for at least one hallmark, yielding a sensitivity of 100%. For the four compounds that did not extend lifespan or healthspan, each showed statistically significant proximity to at least one hallmark. Three of them (Valdecoxib, GDC-0941, and

Ascorbyl-palmitate) also had CMap data, and two displayed positive $pAGE$ values for at least one hallmark, corresponding to a false-positive rate of 66.6%.

Targets of Experimentally Tested Drugs.– We next report here the molecular targets of the compounds used in the experimental validation. Lifespan- or healthspan-extending compounds and their targets are: Vorinostat (HDAC1, HDAC2, HDAC3, HDAC4, HDAC5, HDAC6, HDAC7, HDAC8, HDAC9, HDAC10, HDAC11, acuC1); LY-294002 (PIK3CG, PIM1); Selumetinib (MAP2K1, MAP2K2, ABCB1, ABCG2, UGT1A1, UGT1A3, CYP1A2, CYP2C8, CYP2C9, CYP2C19, CYP2E1, CYP3A4, CYP3A5, ALB, ORM1, ORM2); Celastrol (TNF, IL1B); KU-0063794 (AKT1, RPS6, EIF4EBP1, MTOR); and AZD-8055 (MTOR). Compounds that did not extend lifespan and their targets are: Valdecoxib (CA3, CA2, CYP3A4, UGT1A9, PTGS2, CYP2C9); GDC-0941 (PIK3CG); Ascorbyl-palmitate (APEX1, CASP1, PPARD); and NVP-BEZ235 (PIK3CG, MTOR).

SI.XV Literature Evidence for the Pipeline's Final Candidates

The SHARP pipeline provides drug candidates predicted to modulate various aging hallmarks (Table. 2 in the main text). Below, we summarize the literature evidence supporting these predictions, with a concise overview presented in Table S13:

Linsitinib is mechanistically linked to the *Deregulated Nutrient Sensing* hallmark because it inhibits IGF1R and INSR, the core receptors of the insulin/IGF-1 signaling (IIS) pathway that governs cellular responses to nutrient availability. By suppressing IIS, linsitinib functionally mimics nutrient deprivation and downregulates downstream AKT–mTOR signaling, a central axis implicated in this hallmark [42].

BMS-754807 is mechanistically linked to the *Deregulated Nutrient Sensing* hallmark be-

cause it acts as a dual IGF1R/INSR inhibitor, suppressing the insulin/IGF–1 signaling (IIS) pathway that regulates cellular metabolic responses to nutrient availability. By reducing IIS and its downstream AKT–mTOR signaling, BMS–754807 functionally mimics nutrient limitation, consistent with this hallmark [43].

GRN163l is directly related to the *Telomere Attrition* hallmark because it is a potent telomerase inhibitor that binds the RNA template of telomerase (hTR), preventing telomere elongation and thereby accelerating telomere shortening. By impairing telomere maintenance, GRN163L triggers the molecular consequences characteristic of this hallmark, including telomere erosion and activation of telomere-driven DNA damage responses [44].

Tertomotide (GV1001) is related to the *Telomere Attrition* hallmark because it is a telomerase–derived peptide vaccine that targets the TERT component of the telomerase complex. Although it does not inhibit telomerase activity directly, Tertomotide engages the telomerase machinery—the central system responsible for maintaining telomere length—thereby linking it to pathways underlying telomere dynamics and telomere–driven cellular senescence [45].

Marimastat is a broad-spectrum matrix metalloproteinase (MMP) inhibitor that prevents excessive degradation of extracellular matrix components. By blocking pathological MMP activity in cystic cholangiocytes, it halts abnormal ECM remodeling, directly counteracting the hallmark of *Changes in the Extracellular Matrix Structure* [46].

Captopril is an angiotensin-converting enzyme (ACE) inhibitor that normalizes collagen turnover and prevents excessive extracellular matrix accumulation. By regulating ECM homeostasis, it directly counteracts the hallmark of *Changes in the Extracellular Matrix Structure* [47].

Pyrazolanthrone is a JNK inhibitor that can prevent certain forms of mitochondrial dysfunction. By modulating stress-activated signaling, it directly counteracts the hallmark of *Mitochondrial Dysfunction* [48].

Benzatropine is a tropane-based dopamine inhibitor that promotes the differentiation of NPC-derived oligodendroglial progenitors into more mature oligodendrocytes ($O4^{+}$ and MBP^{+}). By supporting progenitor commitment and maturation, it directly counteracts the hallmark of *Exhaustion of Stem Cells* [49].

SI.XVI Network drugs

A large-scale experimental screening in which 6,000 drugs were tested for their impact on COVID-19 infection [50] found that direct interactions between drugs and disease genes fail to predict the impact of most drugs. Specifically, 76 of the 77 drugs that were found to be experimentally effective against COVID-19 infection could not be explained by such direct interactions with the viral target proteins.

The network medicine approach we implemented in this study resolves these limitations by integrating information encoded by the entire network structure. The use of network proximity enables the identification of drugs that do not directly target aging genes yet are still expected to exert therapeutic effects. In our case, such "network drugs" account for 83 of the 370 candidates identified by our pipeline (22.4%) for aging hallmarks. These candidates would be missed by approaches that do not consider the full network topology and the respective hallmark modules. See Fig. S21 for the number of network drugs identified for each hallmark.

SI.XVII Baseline ablation analysis

To further strengthen the methodological validation of SHARP, we performed a baseline ablation analysis (Fig. S22) comparing network proximity alone, $pAGE$ alone, and their integration. For the ITP drugs (confidence level 2), the ablation analysis shows that network proximity alone yields a sensitivity of 90.9% with a false positive rate (FPR) of 82.3%. $pAGE$ alone achieves 100% sensitivity but with a higher FPR of 57.1%; however, it cannot serve as a standalone prioritization criterion because it does not assess whether a drug meaningfully impacts the hallmark module, but only evaluates the direction of the transcriptional response. When combined within SHARP, network proximity and $pAGE$ together achieve 100% sensitivity while reducing the FPR to 42.8%, demonstrating improved specificity through their complementary roles. For the independent experimental study, proximity alone yields 100% sensitivity and 100% FPR, while $pAGE$ alone provides similar results as the SHARP.

SI.XVIII Leave-out analysis

Because ITP drugs are partly selected based on prior evidence linking them to longevity pathways, which may originate from the same literature corpus underlying OpenGenes, we performed a leave-out analysis to further assess the robustness of SHARP's predictions. Specifically, we removed the 51 overlapping target genes from OpenGenes (see Supplementary Section SI.XIII) and re-ran the ITP validation (Table S14). Under this stricter setting, SHARP identified 5 of the 8 lifespan-extending ITP drugs, corresponding to a sensitivity of 62.5%, compared to 100% in the original analysis. The drugs no longer recovered were sirolimus, meclizine, and estradiol. Importantly, the majority of validated drugs

remained correctly identified despite the removal of overlapping targets, indicating that the predictions are not primarily driven by direct gene overlap and alleviating concerns regarding potential circularity.

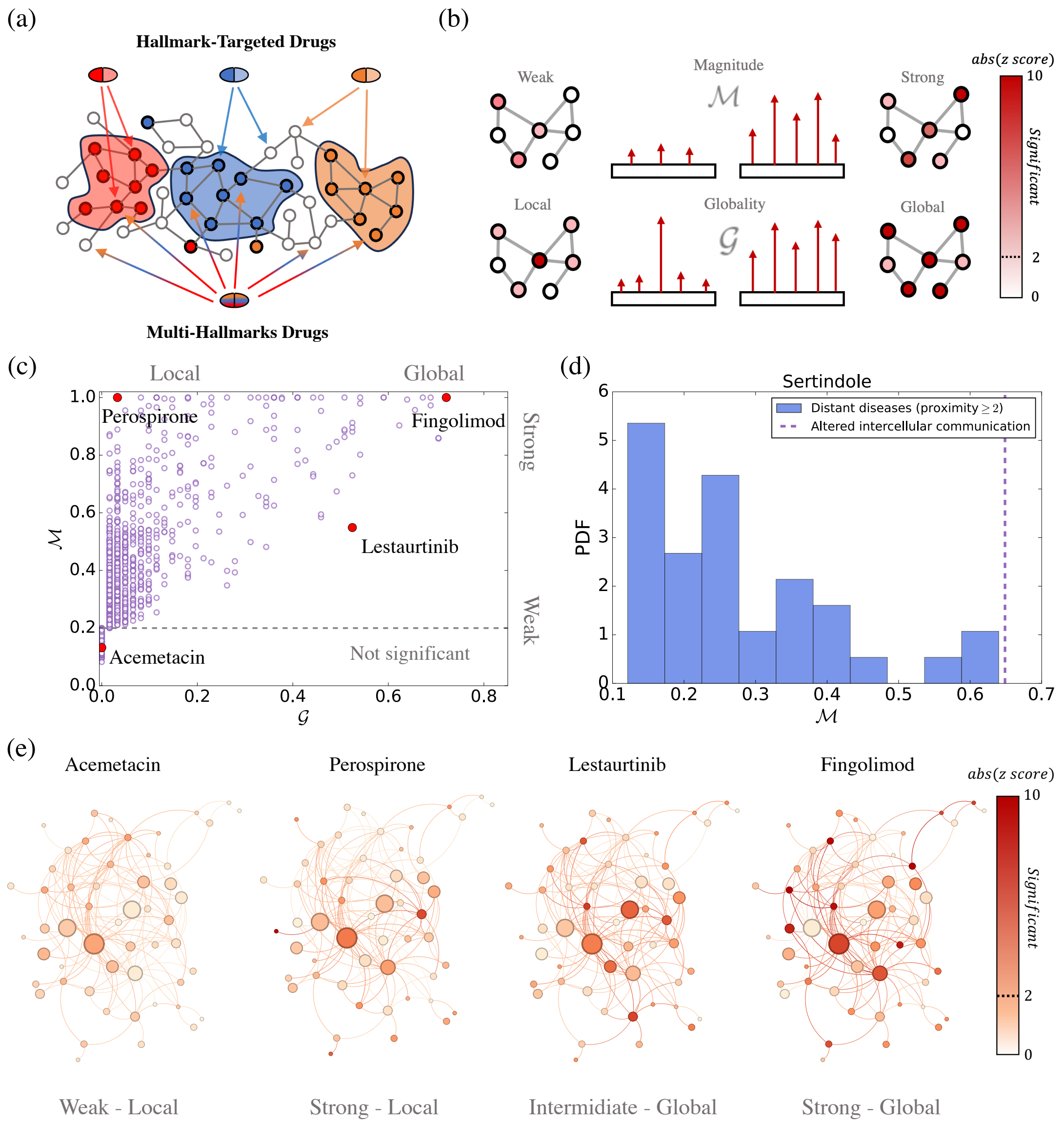

Figure S1: **Perturbation impact of drugs. (a)** Drugs can be repurposed to affect a specific hallmark or multiple hallmarks simultaneously. **(b)** The perturbation impact of a module induced by a drug can be classified by two parameters. The perturbation magnitude ($\mathcal{M}$) characterizes the maximal perturbation score, and perturbation globality ($\mathcal{G}$) characterizes the fraction of the module with a statistically significant perturbation score (|z-score| > 1.96) **(c)** The perturbation parameters ($\mathcal{M}$, $\mathcal{G}$) characterize the impact of a drug on the module functionality. Low values of $\mathcal{M}$ indicate weak impact and high-value strong impact. Low values of $\mathcal{G}$ imply local impact and high-value global impact. The data points are all the drug in the DrugBank and their perturbation parameters ($\mathcal{M}$, $\mathcal{G}$) for the Altered intercellular communication hallmark confidence level 4. **(d)** Network proximity can estimate the impact of a drug on a disease module. Sertindole is one of the candidates for drug-repurposing for the Altered intercellular communication hallmark. When compared to distant disease modules (proximity ≥ 1.96), the perturbation magnitude in the hallmark module is higher. **(e)** Four examples of perturbation impact in Altered intercellular communication hallmark module. Acemetacin shows both weak and local impact. Perospirone shows a strong impact but is local. Lestaurtinib shows intermediate impact and global. Finally, Fingolimod shows a strong and global impact.

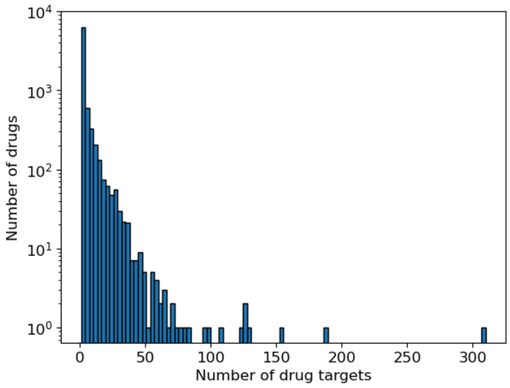

Figure S2: **Drug targets.** Our data of 6442 drugs with at least one target. The distribution of drug targets is characterized by a median of 1 and a mean of 4.024. While most drugs have a low number of targets, some drugs can have a high number of targets.

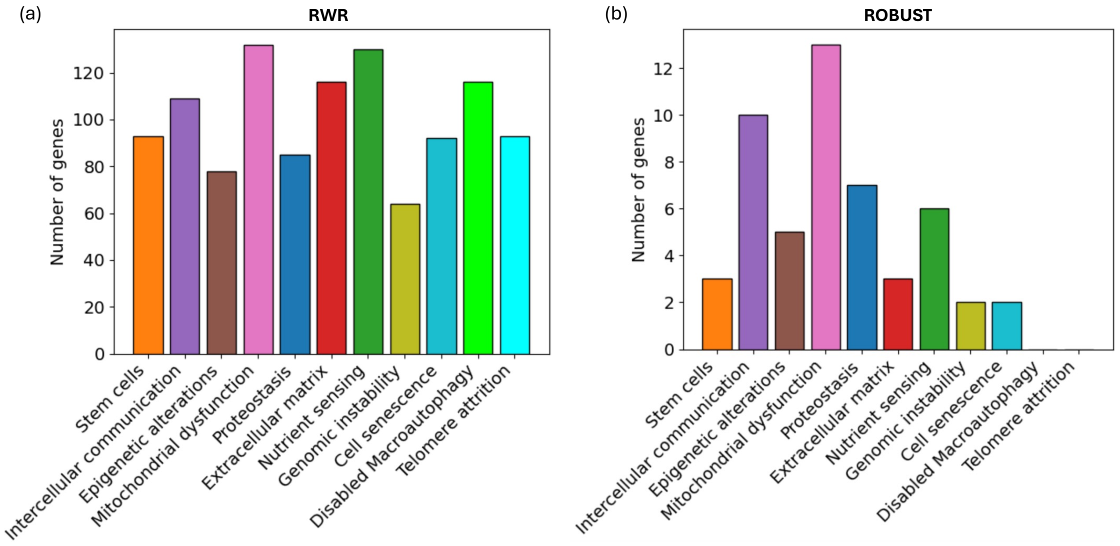

Figure S3: **Classifying unclassified genes using network topology.** The OpenGenes database lists 1,108 aging genes that are not explicitly linked to any aging hallmark. **(a)** To classify them, we used a random walk with a restart probability of 0.2. For each hallmark, we calculated the probability of visiting each unclassified gene and associated each unclassified gene with the hallmark that most frequently visited it. This figure shows the number of unclassified genes that are associated with each hallmark by the random walk method. **(b)** For each hallmark, we used the ROBUST algorithm, starting from hallmark-specific genes as seeds. Then we measure how many unclassified genes were absorbed into the extended hallmark module created by ROBUST. Here we used the ROBUST parameters $\alpha = 0.25$ (diffusion restart), $\beta = 0.9$ (hub-bias correction), $n = 30$ (perturbation runs), $\tau = 0.1$ (robustness threshold), and $\gamma = 1.0$ (locality penalty).

OpenGenes – Hallmarks of Aging	GWAS Timmers *et al* (2019) 10468 aging genes	GWAS Timmers *et al* (2020) 9511 aging genes	GWAS Zenin *et al* (2019) 11525 aging genes	GTEx Jia *et al* (2018) 1573 aging genes	Transcriptional landscape Peters *et al* (2015) 1364 aging genes	Aging Atlas 503 aging genes
Exhaustion of stem cells	0.0026	0.0047	0.01	0.2	0.027	6.89e-15
Altered intercellular communication	2.58e-05	3.79e-06	0.0023	0.13	6.37e-05	1.73e-78
Epigenetic alterations	0.0014	6.75e-05	0.0006	0.68	4.18e-08	1.26e-90
Mitochondrial dysfunction	0.98	0.77	0.88	4.42e-05	0.0054	3.05e-30
Loss of proteostasis	0.55	0.0039	0.04	6.46e-05	0.012	1.35e-29
Changes in the extracellular matrix structure	0.0062	0.13	0.00097	0.32	0.63	4.47e-07
Deregulated nutrient sensing	0.0012	0.0037	0.0045	0.44	0.035	8.25e-48
Genomic instability	0.31	0.54	0.4	0.12	0.0022	4.71e-60
Cell senescence	0.23	0.0014	0.017	0.74	0.00056	4.52e-32
Disabled macroautophagy	0.13	0.008	0.14	0.027	0.018	4.18e-08
Telomere attrition	0.58	0.22	0.026	0.54	0.11	3.37e-26

Table S1: **Enrichment of the OpenGenes database with other aging genomics databases.** The following data sets are considered: GWAS studies [2, 3, 4], GTEx [5], blood gene expression meta-analysis studies [6], and Aging Atlas [7].

OpenGenes – Hallmarks of Aging	Stroke 272 genes	Diabetes mellitus type 2 811 genes	Alzheimer's disease 764 genes	Coronary artery disease 569 genes	Pulmonary disease chronic obstructive 497 genes
Exhaustion of stem cells	0.036	0.0056	0.016	0.19	0.044
Altered intercellular communication	4.48e-06	2.31e-05	0.00064	0.004	1.51e-05
Epigenetic alterations	4.61e-05	0.0037	0.037	0.085	0.25
Mitochondrial dysfunction	0.18	0.0053	0.041	0.27	0.55
Loss of proteostasis	0.092	0.71	0.00048	0.013	0.2
Changes in the extracellular matrix structure	0.0017	0.73	0.033	0.0004	0.0009
Deregulated nutrient sensing	0.38	0.00016	0.0043	0.062	0.7
Genomic instability	0.42	0.18	0.13	0.69	0.56
Cell senescence	0.15	0.056	0.86	0.43	0.72
Disabled macroautophagy	0.42	0.08	0.20	0.69	0.079
Telomere attrition	1.0	1.0	1.0	0.67	1.0

Table S2: **Enrichment of the OpenGenes database with Aging-related diseases.** The aging-related diseases Stroke, Diabetes mellitus type 2, Alzheimer's disease, Coronary artery disease, and Pulmonary disease chronic obstructive are enriched with various hallmarks of aging.

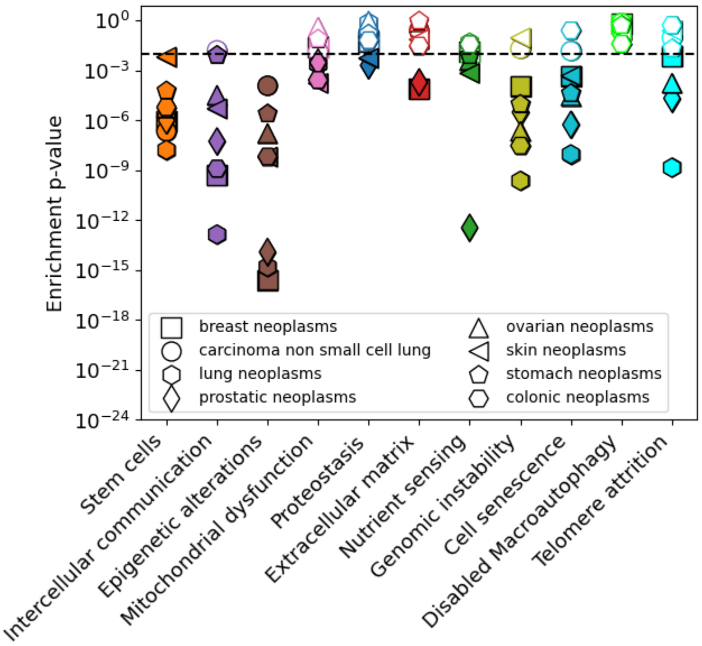

Figure S4: **Enrichment of the hallmarks of aging with cancer.** The rate of cancer diagnostics increases with age for different types of cancer. Therefore, most of the hallmarks of aging are enriched with genes associated with different types of cancer. Breast neoplasms enriched with 8 out of 11 hallmarks, Carcinoma non-small cell lung with 8, Lung neoplasms with 7, Prostatic neoplasms with 9, Ovarian neoplasms with 8, Skin neoplasms with 8, Stomach neoplasms with 5 and Colonic neoplasms with 5 out of 11 different hallmarks.

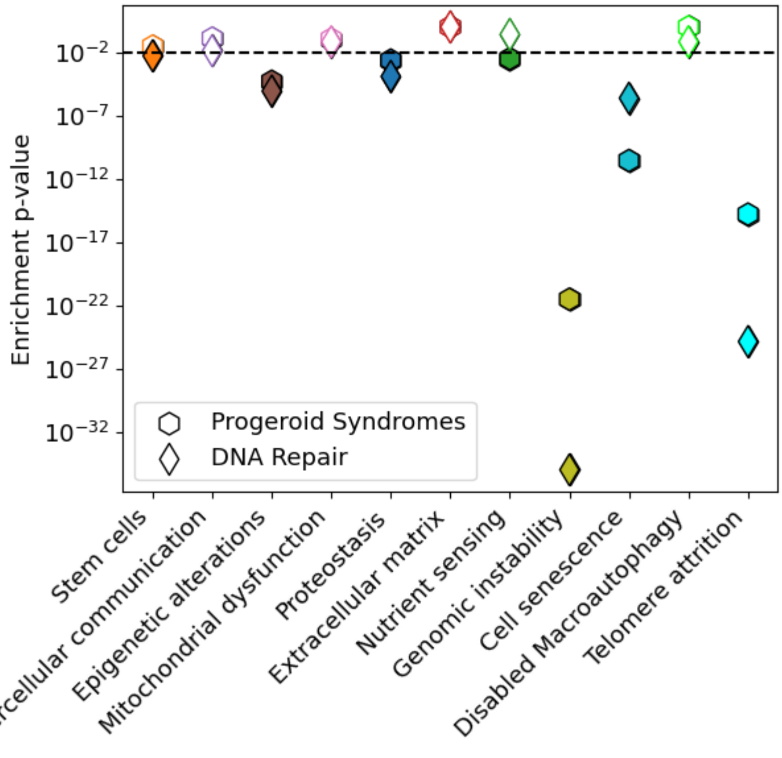

Figure S5: **DNA repair and Progeriod syndromes genes.** Some of the hallmarks of aging are enriched with DNA repair genes and Progeroid syndromes genes. Here we used Aging-associated genes with confidence levels 1-4.

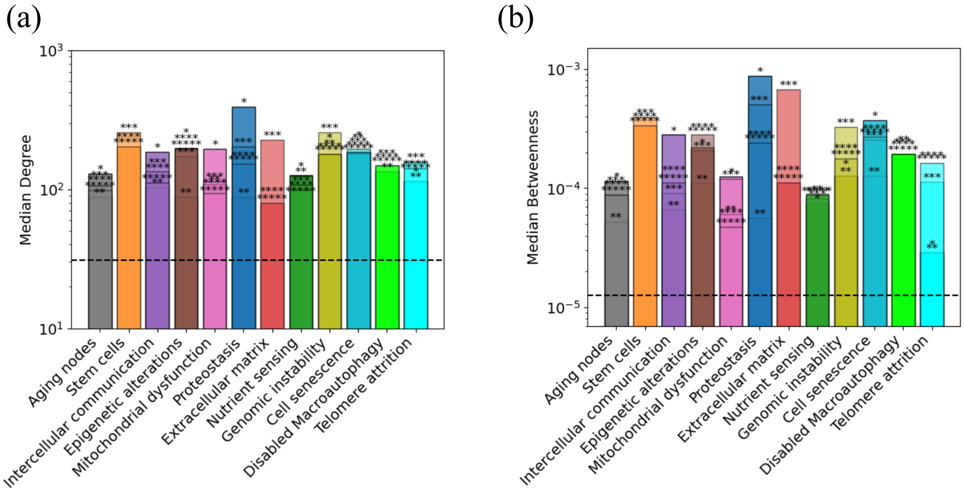

Figure S6: **Betweenness and median degree.** The aging genes show high centrality both in (a) median degree and (b) median betweenness across all the hallmarks of aging compared to the entire network (black dashed line). Stars indicate the confidence level considered (1-5).

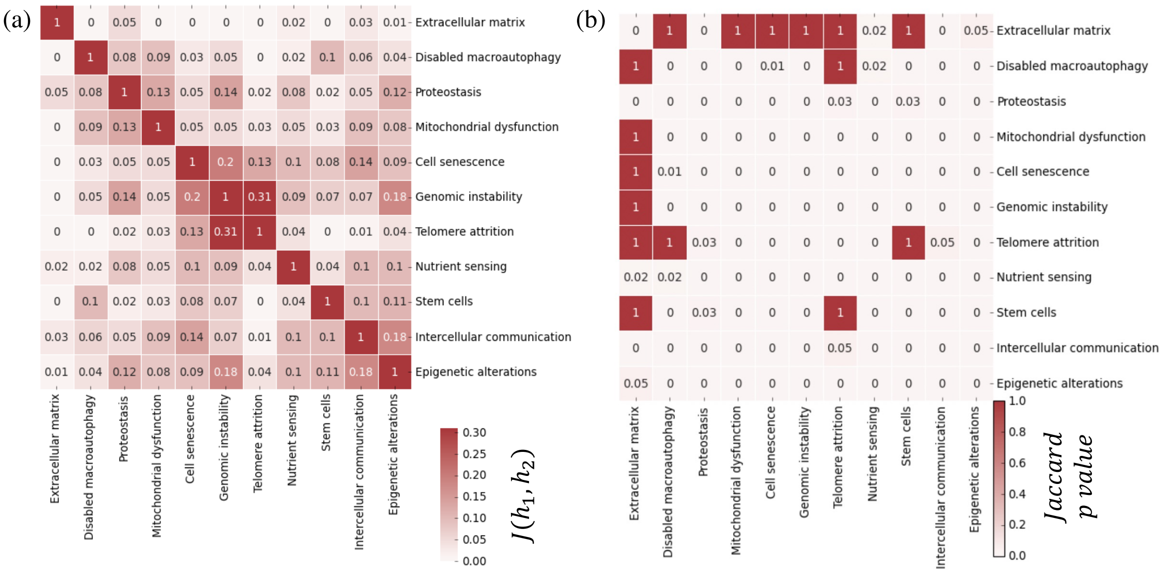

Figure S7: **Jaccard index of the hallmarks of aging. (a)** The Jaccard index measures the interconnectivity between pairs of hallmarks of aging. The hallmarks of Genomic instability, cell senescence, and Telomere attrition show the highest Jaccard value (center) while the Changes in the extracellular matrix structure hallmark show almost no overlap with the other hallmarks. **(b)** All non-zero values of the Jaccard index are statistically significant.

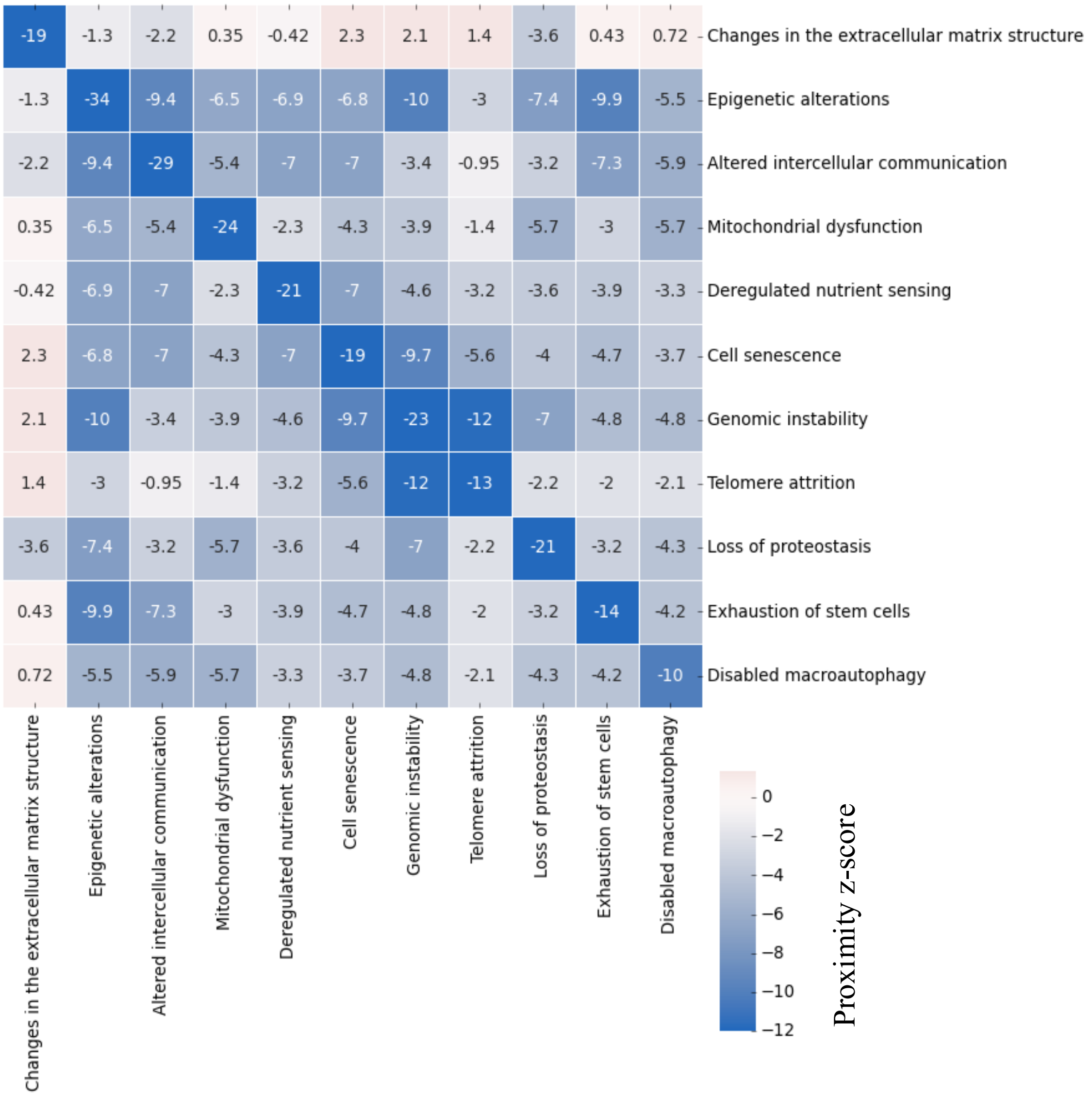

Figure S8: **Proximity of the hallmarks of aging.** The z-score of the network-based proximity measurement draws a similar picture where most hallmarks show statistically significant proximity (z-score < -1.96) except for the Changes in the extracellular matrix structure hallmark.

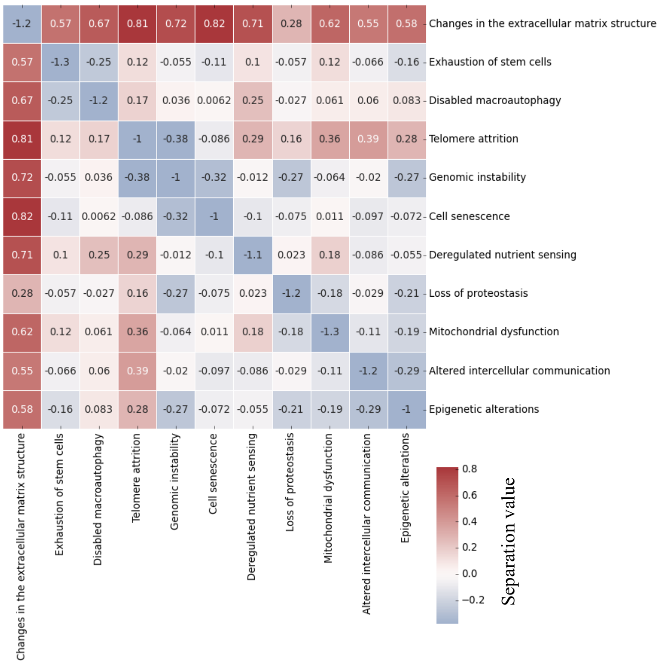

Figure S9: **Separation of the hallmarks of aging.** The network-based separation measurement between pairs of hallmarks of aging. While the separation of the Changes in the extracellular matrix structure hallmark is positive with the other hallmarks ($S > 0$) many other pairs of the hallmarks are overlapping ($S < 0$).

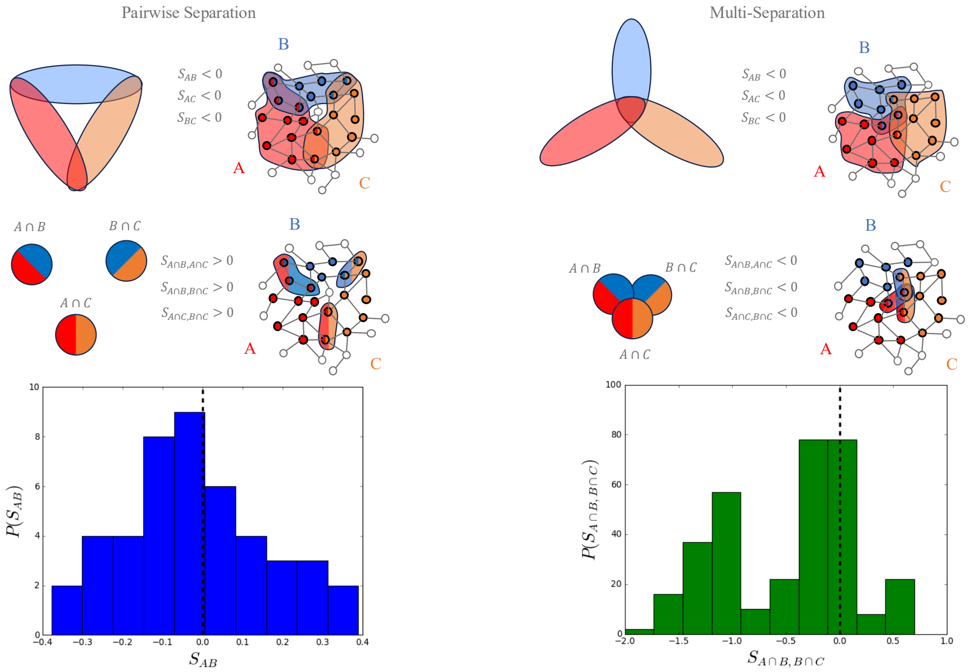

Figure S10: **The core of the longevity module** Multiple hallmarks may overlap pairwise but will not have a common origin to all of them. The separation of the gene sets intersections unveils that the overlap of the modules is at the same network neighborhood, suggesting the longevity module has a core of genetic origin to all hallmarks of aging.

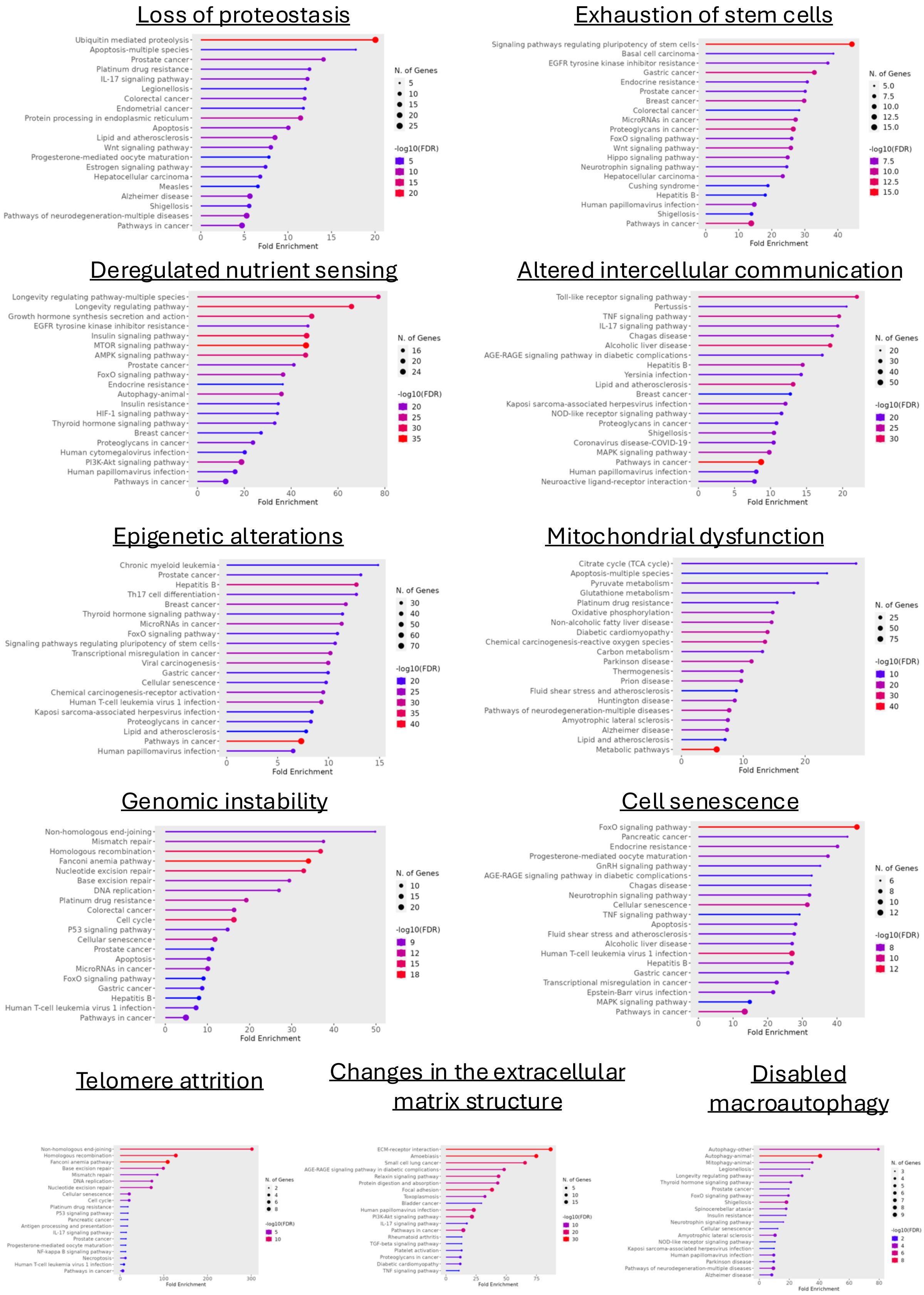

Figure S11: **KEGG pathway analysis of the hallmarks of aging.**

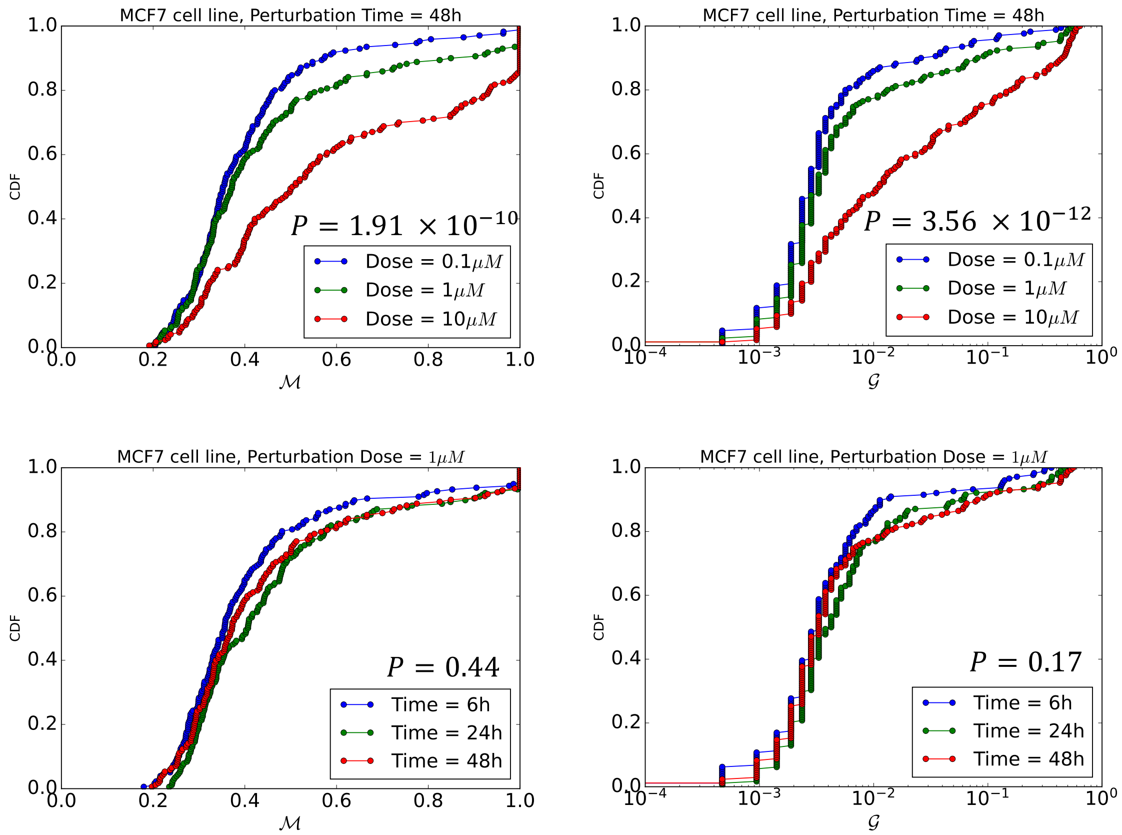

Figure S12: **Impact of perturbation time and dose on the perturbation magnitude and globality in the longevity module for the MCF7 cell line.** While both $\mathcal{M}$ and $\mathcal{G}$ increase with the dose, the perturbation time remains statistically unaffected.

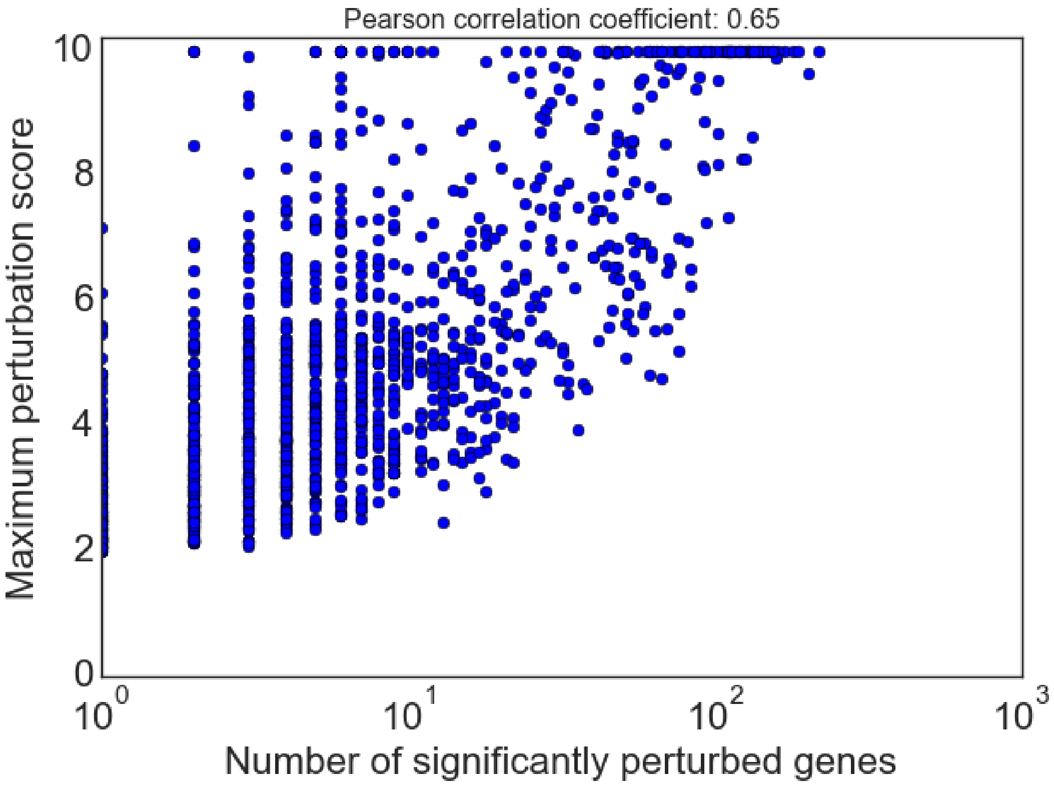

Figure S13: **Correlation of the maximum perturbation score and the number of significantly perturbed genes.** For all the drugs in the DrugBank we measured both the maximum perturbation score and the number of statistically significant perturbed genes in the longevity module. Both measurements are correlated with a Pearson correlation coefficient 0.65.

Score	Drug name	Proximity significance across confidence levels	*pAGE*
10 (1)	astaxanthin	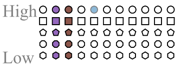	N/A
8 (1)	captopril	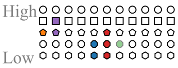	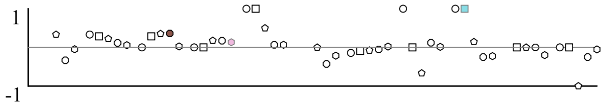
5 (2)	phenylbutyric acid	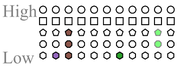	N/A
3 (1)	sirolimus	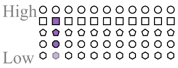	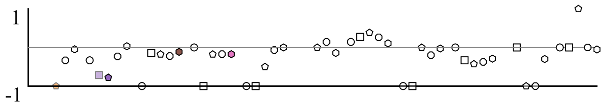
1 (1)	canagliflozin	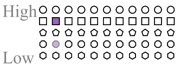	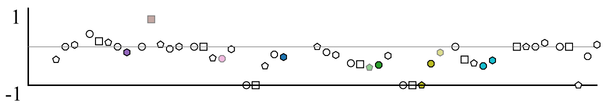
1 (0)	masoprocol	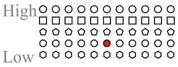	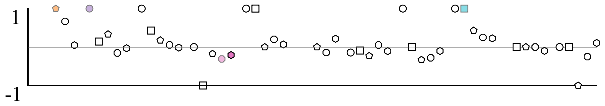
0 (3)	acarbose	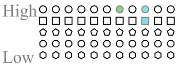	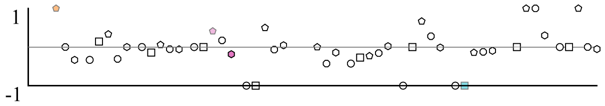
0 (1)	meclizine	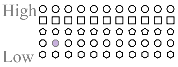	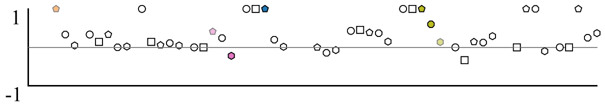
0 (1)	metformin	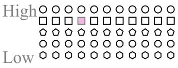	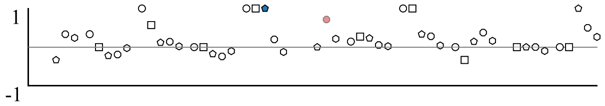
0 (1)	estradiol	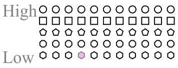	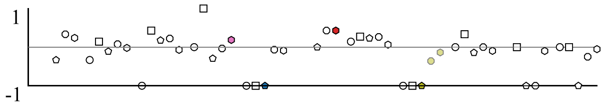
0 (0)	glycine	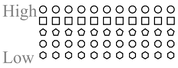	N/A

Table S3: **Drug-repurposing score for drugs that extend life in mice from the ITP database.** Among the 11 drugs that extend lifespan in mice from the ITP database 6 drugs show statistically significant proximity (z-score < -1.96) for at least one hallmark. Additional four show marginal significance (z-score < -1.645). The number of significant (full color) and marginal (transparent color) proximity for different hallmark levels is shown. Non-significant are shown in white. The $pAGE$ value is shown for each level and for each hallmark. Significant $pAGE$ values (|z-score| > 1.96) are shown with full color while non-significant are shaded.

Score	Drug name	Proximity significance across confidence levels	*pAGE*
13 (5)	aspirin		
11 (3)	dasatinib		
4 (3)	fisetin		N/A
3 (4)	nicotinamide mononucleotide		N/A
3 (2)	mesalazine		
3 (1)	sirolimus		
3 (3)	canakinumab		N/A
2 (0)	spermidine		N/A
1 (1)	everolimus		
1 (0)	exenatide		N/A
1 (1)	liraglutide		N/A
0 (0)	dulaglutide		N/A
0 (0)	semaglutide		N/A
0 (3)	acarbose		
0 (0)	niacin		
0 (1)	metformin		
0 (2)	quercetin		

Table S4: **Drug-repurposing score for drugs currently under clinical trials for anti-aging.** Among the 17 drugs currently under clinical trials for anti-aging medicine for humans [51] 11 showed statistically significant proximity (z-score < -1.96) for at least one hallmark. Additional three show marginal significance (z-score < -1.645). Additional four show marginal significance (z-score < -1.645). The number of significant (full color) and marginal (transparent color) proximity for different hallmark levels is shown. Non-significant are shown in white. The $pAGE$ value is shown for each level and for each hallmark. Significant $pAGE$ values (|z-score| > 1.96) are shown with full color while non-significant are shaded.

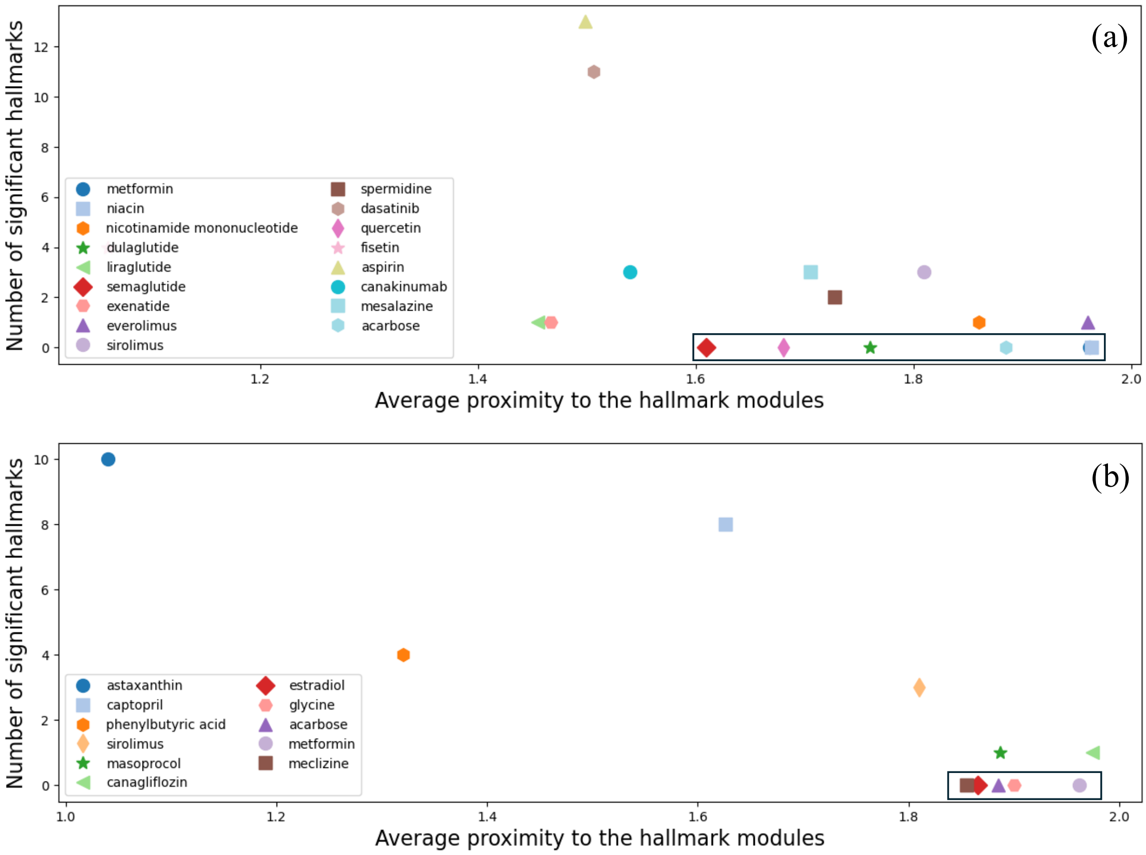

Figure S14: **Missed aging drugs.** **(a)** Among the 17 drugs currently under clinical trials, our pipeline missed 6 with zero significant hallmarks: dulaglutide, metformin, quercetin, niacin, semaglutide, and acarbose. The targets of these drugs are distant from the hallmark modules with an average proximity higher than 1.6. Indeed, other drugs are also distant and still have statistically significant proximity to some hallmarks (e.g. everolimus with an average proximity of 1.96 and 1 significant hallmark), this difference is due to marginal z-score. While metformin shows z-score $= -1.87 > -1.96$ for mitochondrial dysfunction level 2, everolimus shows z-score $= -2.05 < -1.96$ for Altered intercellular communication level 4. Hence, the difference results from statistical error near marginal significance (z-score $= -1.96$) or due to the difference in the degrees of the drug targets. **(b)** Among the 11 drugs that increase lifespan in mice from the ITP database, our pipeline missed 5 with zero significant hallmarks: metformin, glycine, acarbose, estradiol, and meclizine. The targets of these drugs are distant from the hallmark modules with an average proximity higher than 1.8.

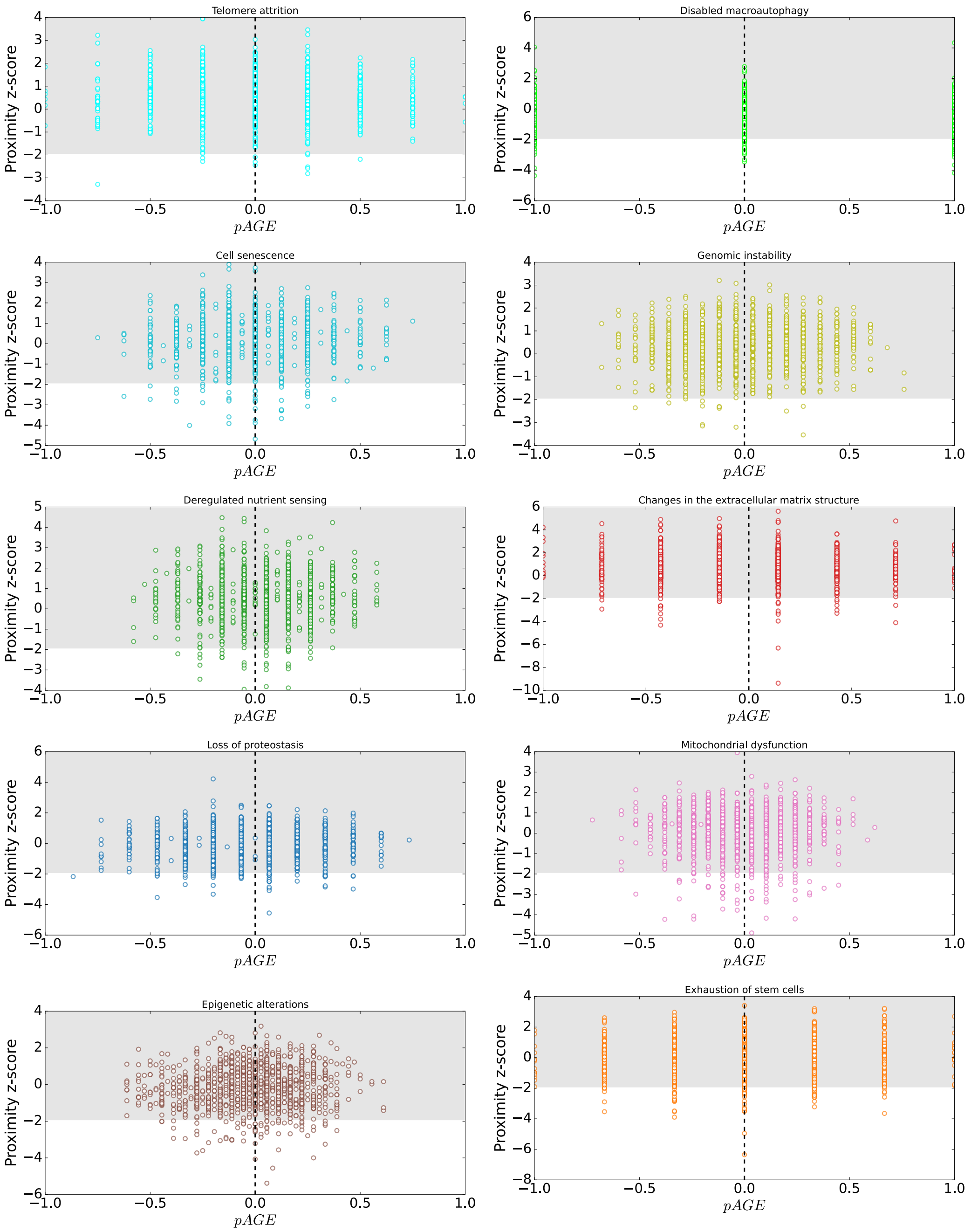

Figure S15: **Proximity and $pAGE$ of drugs from the DrugBank with CMap data in the hallmarks of aging.** Insignificant values of proximity are colored in grey.

Score	Drug name	Proximity significance across confidence levels	Cancer drug
31 (4)	pimasertib	High 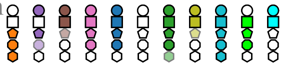 Low	Yes
30 (2)	cambinol	High 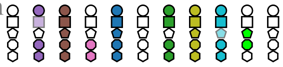 Low	Yes
30 (1)	selisistat	High 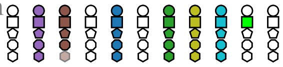 Low	No
26 (0)	perifosine	High 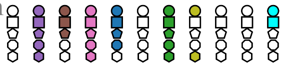 Low	Yes
26 (1)	archexin	High 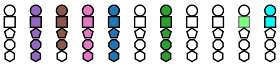 Low	Yes
24 (4)	tertomotide	High 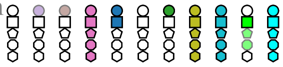 Low	Yes
23 (6)	alanosine	High 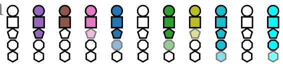 Low	Yes
23 (3)	minocycline	High 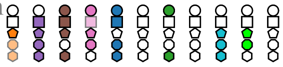 Low	No
23 (5)	xl765	High 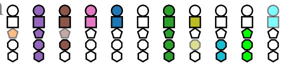 Low	Yes
21 (8)	pam2csk4	High 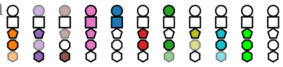 Low	No

Table S5: **Drug-repurposing for Multi-hallmark drugs ranked by the number of hallmarks.** Top candidates are ranked by the number of hallmarks and levels of confidence that are statistically significant with a maximum score of 55 for all 5 confidence levels across all 11 hallmarks. These network drug predictions do not have Cmap data and are predicted only based on the network structure. Significant levels are shown with full color while marginal significant are shaded.

Score	Drug name	Proximity significance across confidence levels	$pAGE$
23 (3)	minocycline		
19 (3)	acemetacin		
18 (2)	pyrazolanthrone		
17 (4)	tivozanib		
17 (1)	tesaglitazar		
17 (6)	fexaramine		
17 (4)	pimecrolimus		
16 (9)	amuvatinib		
16 (2)	neomycin		
16 (0)	amlexanox		
16 (5)	staurosporine		

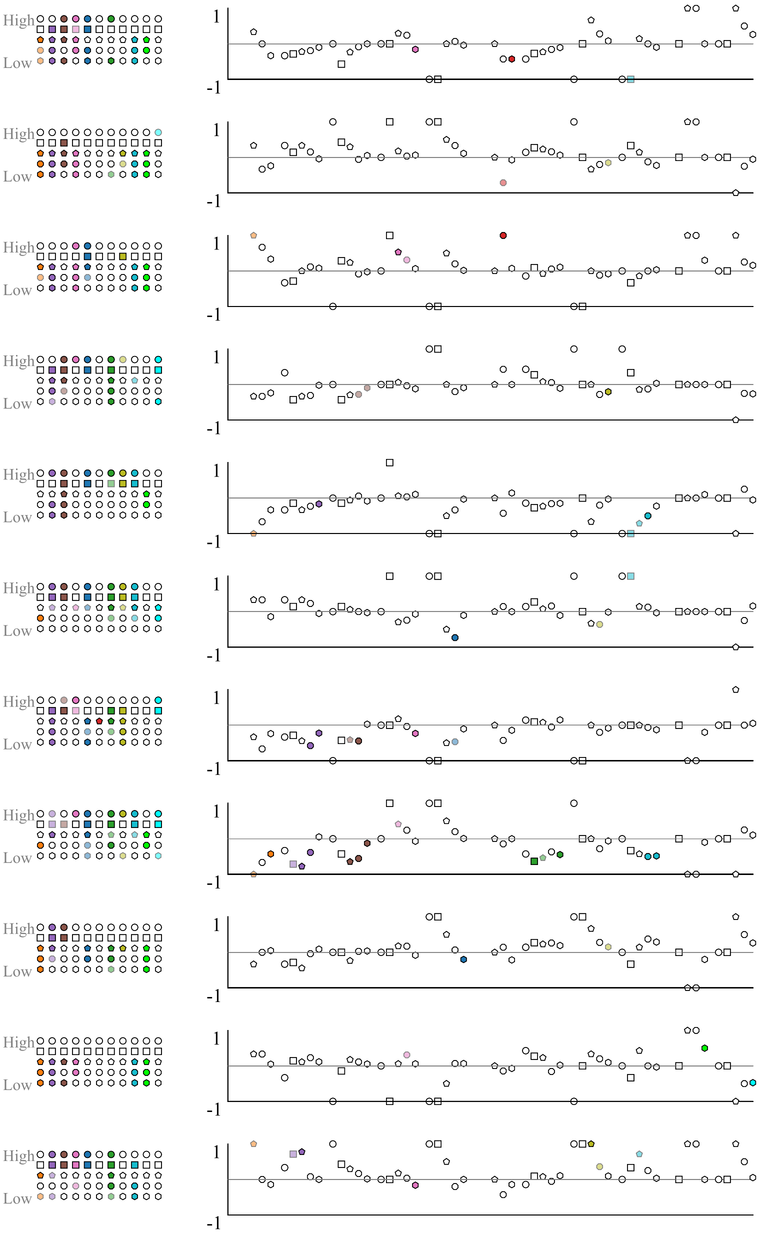

Table S6: **Drug-repurposing for Multi-hallmark drugs ranked by the number of hallmarks with $pAGE$ value.** Top candidates are ranked by the number of hallmarks and levels of confidence that are statistically significant with a maximum score of 55 for all 5 confidence levels across all 11 hallmarks. Significant levels are shown with full color while marginal significant are shaded. The $pAGE$ value is shown with full color for significant values and shaded otherwise.

Drug name	*Proximity (level 4)* Value	(z-score)	*pAGE*
**Exhaustion of stem cells**			
epirubicin	1.0	(**-3.51)	▲▽
navitoclax	1.0	(**-2.28)	▲▽
**Altered intercellular communication**			
alfuzosin	0.71	(**-5.13)	▲▽
ruxolitinib	1.0	(**-2.39)	▲▽
labetalol	1.25	(**-3.39)	▲▽
isotretinoin	0.75	(**-3.06)	▲▽
etodolac	1.0	(**-3.86)	▲▽
gsk-1059615	0.5	(**-2.46)	▲▽
calcitriol	1.2	(**-1.99)	▲▽
**lestaurtinib**	1.0	(**-2.08)	▽
fexofenadine	1.42	(**-2.49)	▲▽
capsaicin	1.27	(**-2.35)	▲▽
**acitretin**	0.88	(**-2.79)	▽
pyrazolanthrone	0.75	(**-2.75)	▲▽
**Epigenetic alterations**			
**mocetinostat**	0.33	(**-3.6)	▽
decitabine	0.95	(**-1.98)	▲▽
ciglitazone	1.0	(-1.39)	▲▽
acitretin	1.0	(-1.07)	▲▽
calcifediol	1.0	(**-2.57)	▲▽
**entinostat**	0.73	(**-2.95)	▽
panobinostat	0.86	(**-2.87)	▲▽
**Mitochondrial dysfunction**			
navitoclax	0.67	(**-2.62)	▲▽
**pyrazolanthrone**	0.75	(**-3.03)	▲
**Loss of proteostasis**			
**isotretinoin**	1.0	(**-2.09)	▽
pyrazolanthrone	1.0	(*-1.78)	▲▽
**Changes in the extracellular matrix structure**			
**alisertib**	1.0	(-1.18)	▲
**Deregulated nutrient sensing**			
wortmannin	0.75	(**-2.95)	▲▽
**Genomic instability**			
**gsk-1059615**	0.5	(**-2.35)	▽
veliparib	0.5	(**-2.37)	▲▽
**Cell senescence**			
farnesol	1.0	(**-2.4)	▲▽
**epirubicin**	1.0	(**-2.74)	▲
pyrazolanthrone	0.5	(**-3.89)	▲▽
**Disabled macroautophagy**			
**carbetocin**	1.0	(**-3.14)	▲
**gsk-1059615**	1.0	(**-1.99)	▽
navitoclax	1.0	(**-2.97)	▲▽
**epirubicin**	1.0	(**-4.36)	▽
**pyrazolanthrone**	1.25	(**-2.29)	▲
**Telomere attrition**			
cct-018159	1.0	(-0.45)	▲▽
veliparib	0.5	(**-2.52)	▲▽
**alvocidib**	1.25	(-1.01)	▲
**plx-4720**	1.0	(-1.21)	▲

Table S7: **List of drugs used for comparing $pAGE$ values between cell lines. Here the $pAGE$ values of the MCF7 cell line is shown.**

Drug name	*Proximity (level 4)* Value	(z-score)	*pAGE*
**Exhaustion of stem cells**			
**epirubicin**	1.0	(**-3.51)	▲
**navitoclax**	1.0	(**-2.28)	▽
**Altered intercellular communication**			
**alfuzosin**	0.71	(**-5.13)	▲
ruxolitinib	1.0	(**-2.39)	▲▽
labetalol	1.25	(**-3.39)	▲▽
isotretinoin	0.75	(**-3.06)	▲▽
**etodolac**	1.0	(**-3.86)	▲
**gsk-1059615**	0.5	(**-2.46)	▽
calcitriol	1.2	(**-1.99)	▲▽
lestaurtinib	1.0	(**-2.08)	▲▽
fexofenadine	1.42	(**-2.49)	▲▽
capsaicin	1.27	(**-2.35)	▲▽
**acitretin**	0.88	(**-2.79)	▽
**pyrazolanthrone**	0.75	(**-2.75)	▽
**Epigenetic alterations**			
mocetinostat	0.33	(**-3.6)	▲▽
**decitabine**	0.95	(**-1.98)	▲
ciglitazone	1.0	(-1.39)	▲▽
**acitretin**	1.0	(-1.07)	▲
calcifediol	1.0	(**-2.57)	▲▽
entinostat	0.73	(**-2.95)	▲▽
**panobinostat**	0.86	(**-2.87)	▲
**Mitochondrial dysfunction**			
**navitoclax**	0.67	(**-2.62)	▽
**pyrazolanthrone**	0.75	(**-3.03)	▲
**Loss of proteostasis**			
isotretinoin	1.0	(**-2.09)	▲▽
pyrazolanthrone	1.0	(*-1.78)	▲▽
**Changes in the extracellular matrix structure**			
alisertib	1.0	(-1.18)	▲▽
**Deregulated nutrient sensing**			
**wortmannin**	0.75	(**-2.95)	▽
**Genomic instability**			
gsk-1059615	0.5	(**-2.35)	▲▽
veliparib	0.5	(**-2.37)	▲▽
**Cell senescence**			
**farnesol**	1.0	(**-2.4)	▽
**epirubicin**	1.0	(**-2.74)	▲
pyrazolanthrone	0.5	(**-3.89)	▲▽
**Disabled macroautophagy**			
**carbetocin**	1.0	(**-3.14)	▲
gsk-1059615	1.0	(**-1.99)	▲▽
**navitoclax**	1.0	(**-2.97)	▽
**epirubicin**	1.0	(**-4.36)	▲
**pyrazolanthrone**	1.25	(**-2.29)	▲
**Telomere attrition**			
cct-018159	1.0	(-0.45)	▲▽
veliparib	0.5	(**-2.52)	▲▽
**alvocidib**	1.25	(-1.01)	▲
plx-4720	1.0	(-1.21)	▲▽

Table S8: **List of drugs used for comparing $pAGE$ values between cell lines. Here the $pAGE$ values of the WI38 cell line is shown.**

Drug name	Proximity (level 4) Value	(z-score)	*pAGE*
**Exhaustion of stem cells**			
**epirubicin**	1.0	(**-3.51)	▲
navitoclax	1.0	(**-2.28)	▲▽
**Altered intercellular communication**			
**alfuzosin**	0.71	(**-5.13)	▲
ruxolitinib	1.0	(**-2.39)	▲▽
**labetalol**	1.25	(**-3.39)	▲
**isotretinoin**	0.75	(**-3.06)	▽
**etodolac**	1.0	(**-3.86)	▲
**gsk-1059615**	0.5	(**-2.46)	▽
calcitriol	1.2	(**-1.99)	▲▽
**lestaurtinib**	1.0	(**-2.08)	▽
**fexofenadine**	1.42	(**-2.49)	▽
capsaicin	1.27	(**-2.35)	▲▽
**acitretin**	0.88	(**-2.79)	▲
pyrazolanthrone	0.75	(**-2.75)	▲▽
**Epigenetic alterations**			
mocetinostat	0.33	(**-3.6)	▲▽
**decitabine**	0.95	(**-1.98)	▲
**ciglitazone**	1.0	(-1.39)	▲
acitretin	1.0	(-1.07)	▲▽
calcifediol	1.0	(**-2.57)	▲▽
entinostat	0.73	(**-2.95)	▲▽
**panobinostat**	0.86	(**-2.87)	▲
**Mitochondrial dysfunction**			
navitoclax	0.67	(**-2.62)	▲▽
**pyrazolanthrone**	0.75	(**-3.03)	▲
**Loss of proteostasis**			
**isotretinoin**	1.0	(**-2.09)	▽
pyrazolanthrone	1.0	(*-1.78)	▲▽
**Changes in the extracellular matrix structure**			
**alisertib**	1.0	(-1.18)	▽
**Deregulated nutrient sensing**			
**wortmannin**	0.75	(**-2.95)	▽
**Genomic instability**			
gsk-1059615	0.5	(**-2.35)	▲▽
veliparib	0.5	(**-2.37)	▲▽
**Cell senescence**			
**farnesol**	1.0	(**-2.4)	▲
epirubicin	1.0	(**-2.74)	▲▽
pyrazolanthrone	0.5	(**-3.89)	▲▽
**Disabled macroautophagy**			
**carbetocin**	1.0	(**-3.14)	▲
**gsk-1059615**	1.0	(**-1.99)	▽
**navitoclax**	1.0	(**-2.97)	▽
**epirubicin**	1.0	(**-4.36)	▲
**pyrazolanthrone**	1.25	(**-2.29)	▲
**Telomere attrition**			
cct-018159	1.0	(-0.45)	▲▽
veliparib	0.5	(**-2.52)	▲▽
**alvocidib**	1.25	(-1.01)	▲
plx-4720	1.0	(-1.21)	▲▽

Table S9: **List of drugs used for comparing $pAGE$ values between cell lines. Here the $pAGE$ values of the IMR90 cell line is shown.**

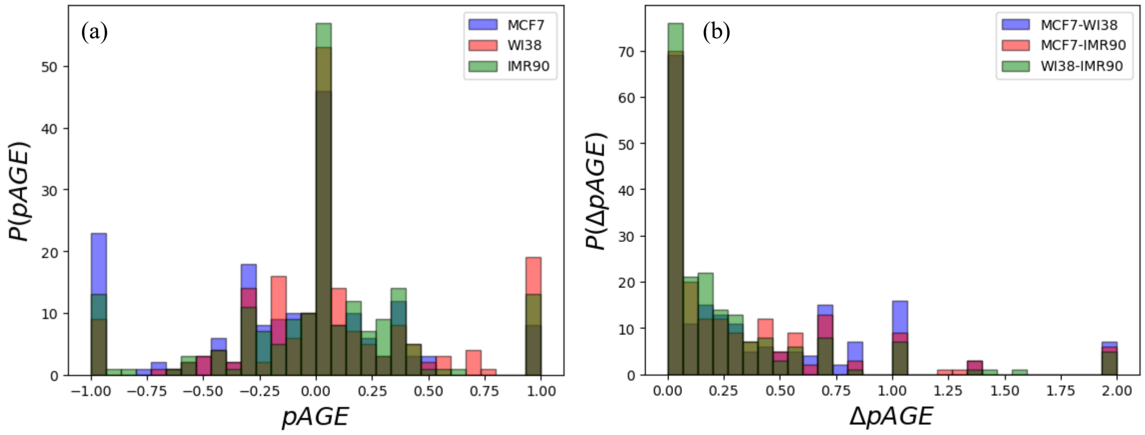

Figure S16: **Comparing the $pAGE$ values between cell lines. (a)** The distribution of the the $pAGE$ values for the cell lines MCF7, WI38, and IMR90. The distributions are similar and outliers are observed for ±1 due to the sensitivity of small modules. **(b)** The distribution of the difference of the $pAGE$ values between the cell lines MCF7, WI38, and IMR90. As shown, the majority of $pAGE$ values show very small differences between cell lines.

Z-score of Hallmark Modules Using Different Interactomes and Methods	The human interactome used in this study (node permutations)	The human interactome used in this study (edge permutations)	Restricted unbiased human interactome (node permutations)	STRING PPI (node permutations)
Exhaustion of stem cells	2.21	1.73	4.59	3.19
Altered intercellular communication	2.69	3.7	10.93	3.67
Epigenetic alterations	1.67	1.66	13.01	3.45
Mitochondrial dysfunction	2.54	3.46	13.02	4.36
Loss of proteostasis	1.74	2.05	0.76	2.00
Changes in the extracellular matrix structure	11.51	11.8	2.77	10.72
Deregulated nutrient sensing	3.89	5.02	18.69	4.25
Genomic instability	2.48	2.44	16.37	2.78
Cell senescence	2.69	3.19	7.78	2.53
Disabled macroautophagy	3.02	2.84	1.76	2.97
Telomere attrition	3.57	5.46	10.4	4.18

Table S10: **Robustness validation with restricted unbiased PPI, the STRING interactome, and edge permutation.**

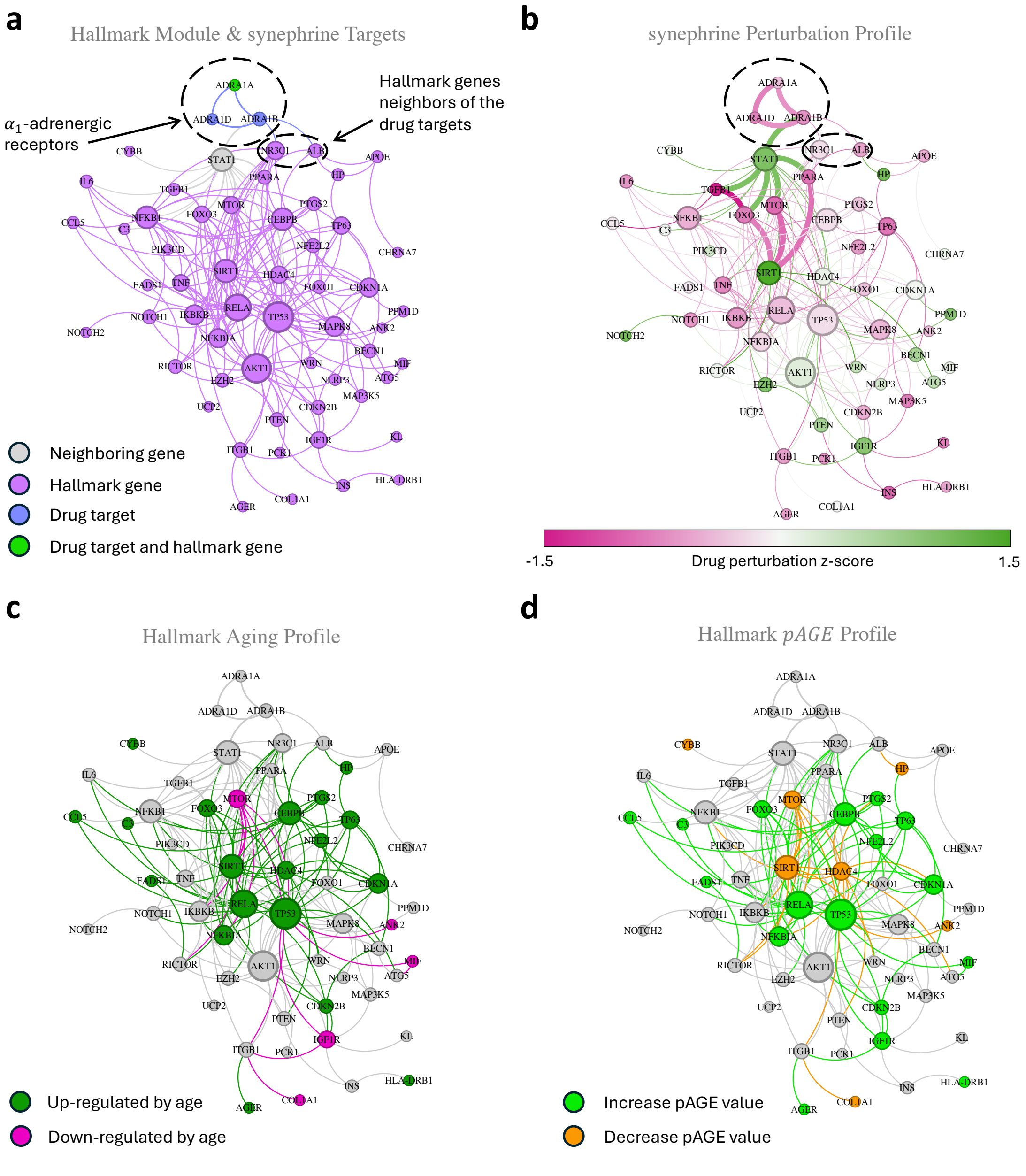

Figure S17: **Proximity and $pAGE$ predicts mechanism of action. a,** synephrine is one of the candidates for drug-repurposing for the Altered intercellular communication hallmark (shown in purple). synephrine targets the proteins ADRA1A, ADRA1B, and ADRA1D as part of the α_1-adrenergic receptor protein group which directly connects to the module by the ALB and NR3C1 genes. The gene ADRA1A is both a drug target and a hallmark gene (green) while ADRA1B and ADRA1D (blue) are nearest neighbors of the module, leading to a statistically significant proximity of 0.66 (see Methods). **b,** Perturbing the MCF7 cell line with synephrine, the drug signature up- (green) and down- (red) regulate genes in the module. The color bar shows the z-score of the perturbation signature for each gene. The perturbation follows a detour path and does not follow the shortest path to the module through the immediate target neighbors ALB and NR3C1. Instead, the target's neighbors STAT1 (not a hallmark gene) is perturbed and transmit the information to the module. **c,** The aging signature marks genes that are up-regulated (green) and down-regulated (red) with age. **d,** The $pAGE$ value is measured according to Eq. 3. By comparing the aging signature and the drug signature, genes with opposite signs (green) increase the $pAGE$ value while genes with similar signs (orange) decrease it, resulting in a statistically significant $pAGE = 0.46$.

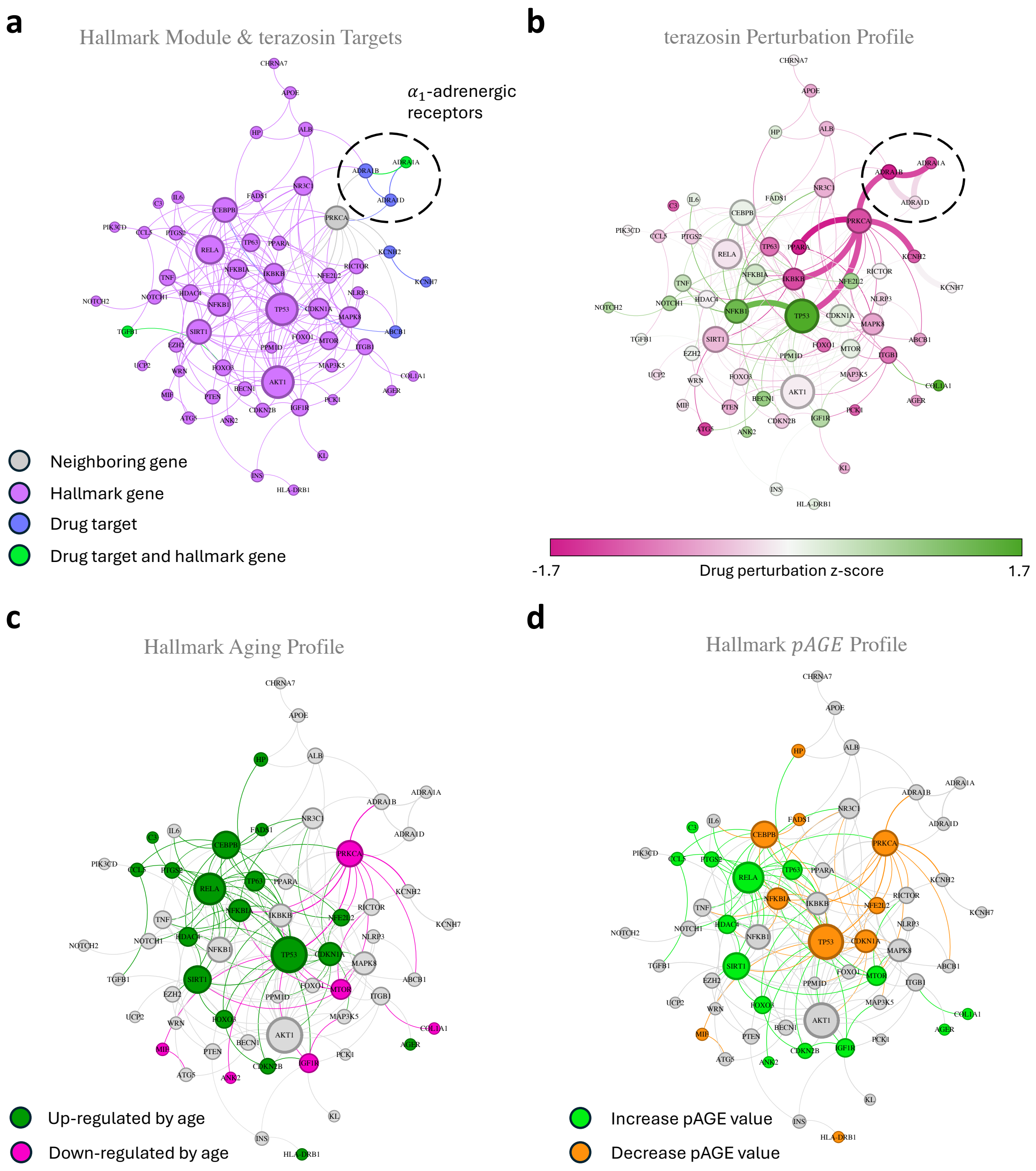

Figure S18: **Proximity and $pAGE$ predicts mechanism of action. a,** terazosin targets and the Altered intercellular communication hallmark module. **b,** Perturbing the MCF7 cell line with terazosin, the drug signature up- (green) and down- (red) regulate genes in the module. The color bar shows the z-score of the perturbation signature for each gene. **c,** The aging signature marks genes that are up-regulated (green) and down-regulated (red) with age. **d,** The $pAGE$ value is measured according to Eq. 3. By comparing the aging signature and the drug signature, genes with opposite signs (green) increase the $pAGE$ value while genes with similar signs (orange) decrease it.

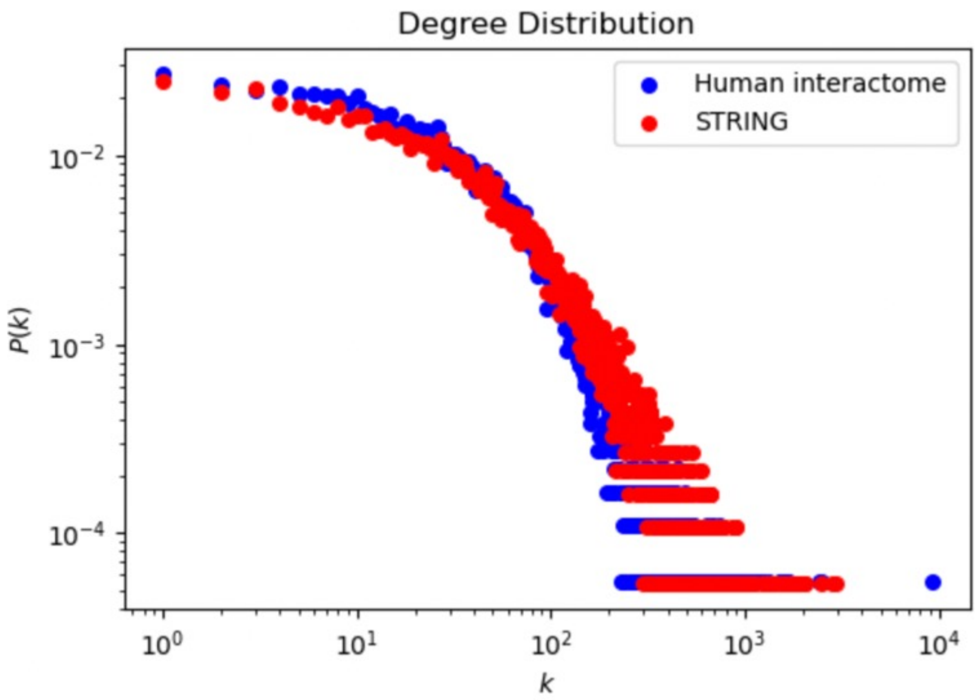

Figure S19: **Comparison of the degree distributions of our human interactome and STRING.**

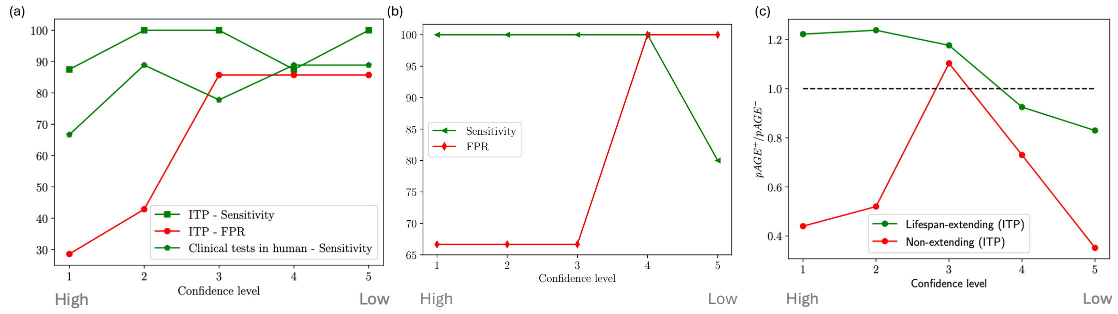

Figure S20: **Effect of Gene Confidence on pAGE Reliability. (a)** Sensitivity and false-positive rate (FPR) for ITP drugs and drugs currently in human clinical trials. **(b)** Sensitivity and false-positive rate (FPR) for the independent experimental study. Low-confidence genes (levels 3–5) introduce substantial noise into the pAGE metric. Focusing on high-confidence genes (levels 1–2) reduces this noise and reveals a clear separation between drugs that extend lifespan and those that do not. **(c)** Ratio of the average number of hallmarks with positive versus negative pAGE values for ITP drugs. Using high-confidence genes (levels 1–2), lifespan-extending drugs show a higher ratio of positive pAGE values across hallmarks, whereas non-extending drugs exhibit more hallmarks with negative pAGE values.

	Drug name	Proximity significance	$pAGE$		Drug name	Proximity significance	$pAGE$
**I**	minocycline			**I**	sulindac		
**I**	resveratrol			**I**	fish oil		N/A
**I**	insulin human		N/A	**I**	enalapril		
**I**	candesartan cilexetil		N/A	**I**	simvastatin		
**I**	alvespimycin			**I**	curcumin		
**I**	dimethyl fumarate		N/A	**I**	ursodeoxycholic acid		N/A
**I**	mycophenolic acid		N/A	**I**	methylene blue		N/A
**I C**	fisetin		N/A	**I**	leucine		N/A
**I**	ursolic acid		N/A				

Table S11: **Drug-repurposing of drugs from the ITP project that failed to extend lifespan.** 17 drugs failed to extend lifespan in mice from the ITP project (**I**) [35]. Among them, 12 showed statistically significant proximity (z-score < -1.96) for at least one hallmark. Additional two shows marginal significance (z-score < -1.645). Four drugs show positive $pAGE$ for at least one hallmark. Statistically significant proximity is shown with full color and marginal significance proximity with transparent color. Non-significant are shown in white. Arrows indicate the $pAGE$ directionality (positive - up or negative - down). Proximity is measured across all confidence levels, and the most significant result is shown. $pAGE$ is measured for level 2 or, if no high-confidence hallmark genes are present, for level 3.

Drug name	Proximity significance	$pAGE$
Vorinostat	□■■□□□■□□■□	▲▽▲▽▽▽▲
LY-294002	■■□■□■■□■■□	▽▽▽▲▽▲▽▽
Selumetinib	□■□■□□□□□□□	▽▽▽▲▽▲▽
Celastrol	□■■□□□□■□□□	N/A
KU-0063794	□■■■■□■■□□■	▽▽▽▲▽▲▽▽
AZD-8055	□■□□■□■■□□■	▽▽▽▲▽▽
Valdecoxib	□■■■□□□□□□□	▽▽▽▽▽▽▽
GDC-0941	■■□■□■■□□■□	▲▽▲▽▲▲▲
Ascorbyl palmitate	□□■■■□□■□□■	▽▽▽▲▽▲▽▽
NVP-BEZ235	■■□■□□■■□■■	N/A

Table S12: **Drug-repurposing of drugs in the experimental study.** Ten drugs were tested, of which six extend healthspan or lifespan (green) and four do not (red). CMap data were available for eight of the ten compounds. Vorinostat, Selumetinib, LY-294002, KU-0063794, and AZD-8055 significantly improved lifespan or healthspan and show both significant network proximity and positive $pAGE$ for at least one hallmark (100 % sensitivity). In contrast, Valdecoxib, GDC-0941, and Ascorbyl-palmitate did not improve lifespan or healthspan. All three show statistically significant proximity to at least one hallmark, but GDC-0941 and Ascorbyl-palmitate display positive $pAGE$ for at least one hallmark, whereas Valdecoxib does not (66.6 % false-positive rate). Arrows indicate the $pAGE$ directionality (positive - up or negative - down). Proximity is measured across all confidence levels, and the most significant result is shown. $pAGE$ is measured for level 2 or, if no high-confidence hallmark genes are present, for level 3.

Hallmark of aging	Predicted drug	Literature support
Deregulated nutrient sensing	linsitinib	linsitinib inhibits IGF1R and INSR.<br>*Macaulay, Valentine M., et al. "Phase I dose-escalation study of linsitinib (OSI-906) and erlotinib in patients with advanced solid tumors." Clinical Cancer Research 22.12 (2016): 2897-2907.*
Deregulated nutrient sensing	bms-754807	bms-754807 inhibits IGF1R and INSR.<br>*Hou, Xiaonan, et al. "Dual IGF-1R/InsR inhibitor BMS-754807 synergizes with hormonal agents in treatment of estrogen-dependent breast cancer." Cancer research 71.24 (2011): 7597-7607.*
Telomere attrition	GRN163l	GRN163l is a telomerase inhibitor<br>*Bär, Christian, and Thomas Thum. "Changing direction: from therapeutic telomerase inhibition to activation?." Circulation Research 120.9 (2017): 1393-1395.*
Telomere attrition	Tertomotide	Tertomotide is a telomerase-derived peptide vaccine<br>*Calcinotto, Arianna, et al. "Cellular senescence: aging, cancer, and injury." Physiological reviews 99.2 (2019): 1047-1078.*
Changes in the extracellular matrix structure	Marimastat	Marimastat is a broad-spectrum matrix metalloproteinase (MMP) inhibitor. marimastat blocks excessive MMP activity in cystic cholangiocytes, thereby preventing pathological ECM remodeling.<br>*Urribarri, Aura D., et al. "Inhibition of metalloprotease hyperactivity in cystic cholangiocytes halts the development of polycystic liver diseases." Gut 63.10 (2014): 1658-1667.*
Changes in the extracellular matrix structure	Captopril	Captopril is an ACE inhibitor. Captopril normalizes collagen turnover and prevents pathological extracellular matrix accumulation.<br>*Laviades, Concepción, et al. "Abnormalities of the extracellular degradation of collagen type I in essential hypertension." Circulation 98.6 (1998): 535-540.*
Mitochondrial dysfunction	Pyrazolanthrone	Pyrazolanthrone is a a JNK inhibitor and can prevent certain forms of mitochondrial dysfunction.<br>*Bennett, Brydon L., et al. "SP600125, an anthrapyrazolone inhibitor of Jun N-terminal kinase." Proceedings of the National Academy of Sciences 98.24 (2001): 13681-13686.*
Exhaustion of stem cells	Benzatropine	Benzatropine is a tropane-based dopamine inhibitor. It was found to promote the differentiation of NPC-derived oligodendroglial progenitors into more mature oligodendrocytes (O4$^+$ and MBP$^+$), helping progenitor / stem-like cells commit and mature.<br>*Ehrlich, Marc, et al. "Rapid and efficient generation of oligodendrocytes from human induced pluripotent stem cells using transcription factors." Proceedings of the National Academy of Sciences 114.11 (2017): E2243-E2252.*

Table S13: **Literature Evidence for the Pipeline's Final Candidates.**

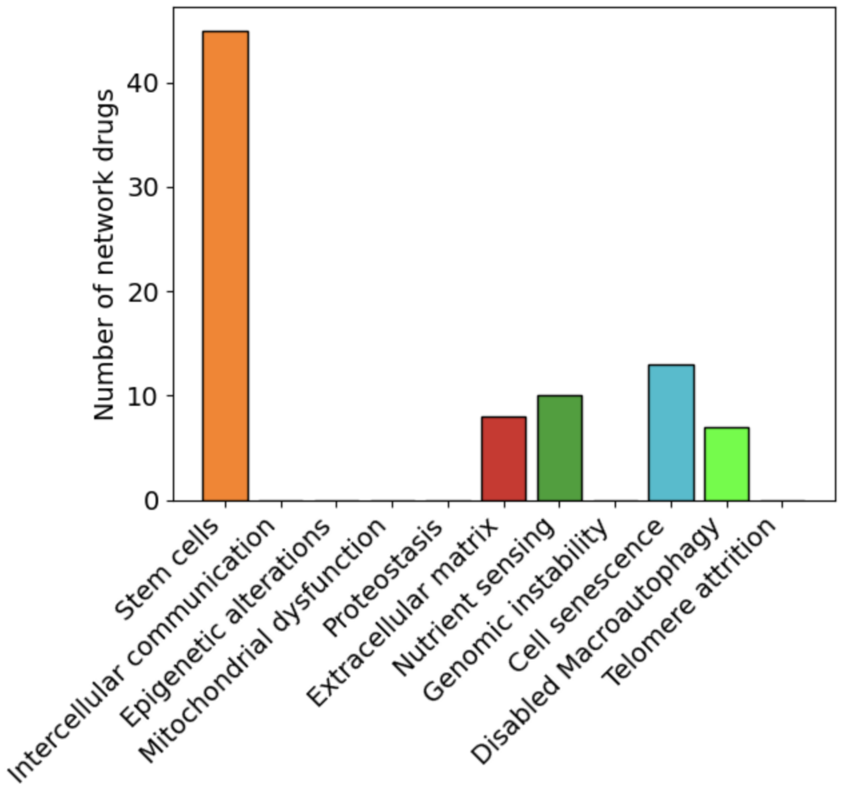

Figure S21: **Number of network drug candidates for each hallmark of aging.** A network drug is defined as a drug whose targets do not include aging-related genes.

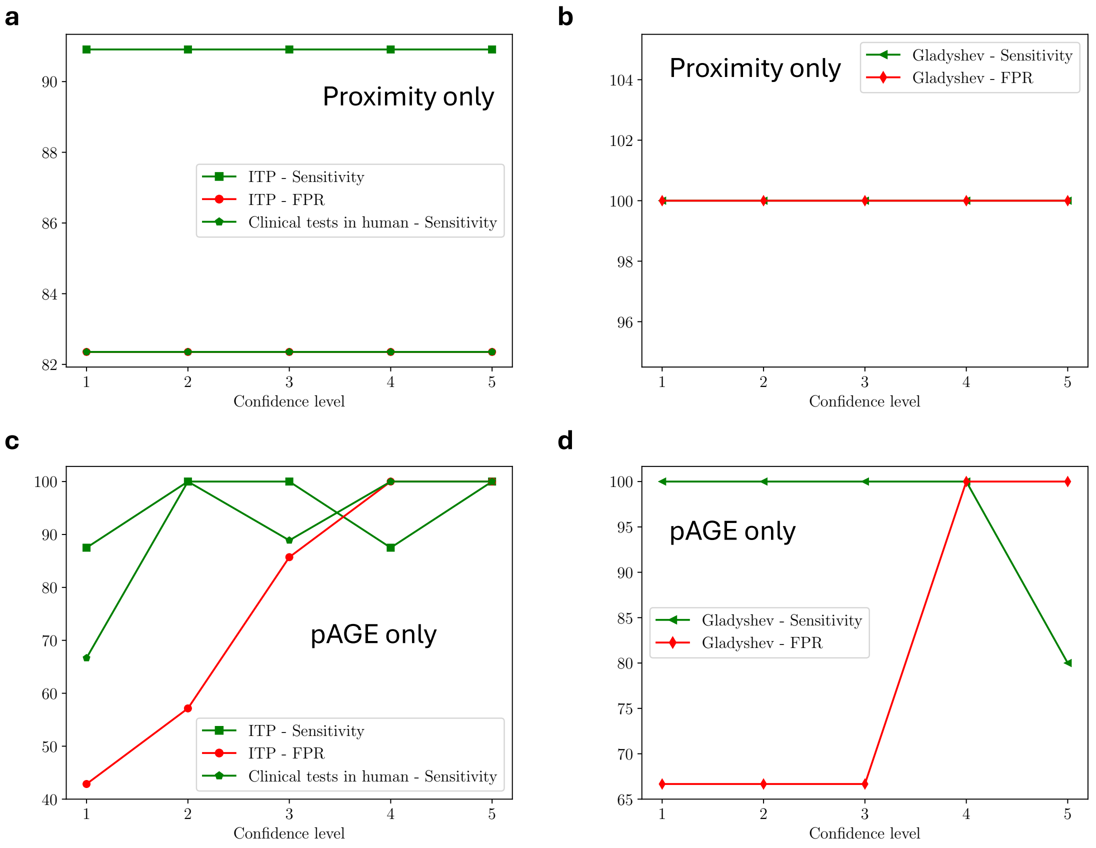

Figure S22: **A baseline ablation analysis. a,** Sensitivity and false-positive rate (FPR) for ITP drugs and drugs currently in human clinical trials using proximity only. **b,** Sensitivity and false-positive rate (FPR) for the independent experimental study using proximity only. **c,** Sensitivity and false-positive rate (FPR) for ITP drugs and drugs currently in human clinical trials using $pAGE$ only. **d,** Sensitivity and false-positive rate (FPR) for the independent experimental study using $pAGE$ only.

	Drug name	Proximity significance	*pAGE*		Drug name	Proximity significance	*pAGE*
C	aspirin			C	semaglutide		N/A
C	dasatinib			I C	acarbose		
I	astaxanthin		N/A	C	niacin		
I	captopril			I	meclizine		
I	phenylbutyric acid		N/A	I C	metformin		
C	fisetin		N/A	C	quercetin		
C	nicotinamide mononucleotide		N/A	I	estradiol		
C	mesalazine			I	glycine		N/A
I C	sirolimus						
C	canakinumab		N/A				
C	spermidine		N/A				
C	everolimus						
C	exenatide		N/A				
C	liraglutide		N/A				
I	canagliflozin						
I	masoprocol						
C	dulaglutide		N/A				

Table S14: **Leave-out analysis.** To assess potential circularity, we removed the 51 genes shared between ITP drug targets and the OpenGenes database and re-ran the SHARP pipeline. This table summarizes the results.

References

[1] Ekaterina Rafikova et al. "Open Genes—a new comprehensive database of human genes associated with aging and longevity". In: *Nucleic Acids Research* 52.D1 (2024), pp. D950–D962.

[2] Paul RHJ Timmers et al. "Genomics of 1 million parent lifespans implicates novel pathways and common diseases and distinguishes survival chances". In: *elife* 8 (2019), e39856.

[3] Paul RHJ Timmers et al. "Multivariate genomic scan implicates novel loci and haem metabolism in human ageing". In: *Nature communications* 11.1 (2020), p. 3570.

[4] Aleksandr Zenin et al. "Identification of 12 genetic loci associated with human healthspan". In: *Communications biology* 2.1 (2019), pp. 1–11.

[5] Kaiwen Jia et al. "An analysis of aging-related genes derived from the genotype-tissue expression project (GTEx)". In: *Cell death discovery* 4.1 (2018), p. 91.

[6] Marjolein J Peters et al. "The transcriptional landscape of age in human peripheral blood". In: *Nature communications* 6.1 (2015), pp. 1–14.

[7] "Aging Atlas: a multi-omics database for aging biology". In: *Nucleic acids research* 49.D1 (2021), pp. D825–D830.

[8] Minoru Kanehisa and Susumu Goto. "KEGG: kyoto encyclopedia of genes and genomes". In: *Nucleic acids research* 28.1 (2000), pp. 27–30.

[9] Alex A Freitas, Olga Vasieva, and João Pedro de Magalhães. "A data mining approach for classifying DNA repair genes into ageing-related or non-ageing-related". In: *BMC genomics* 12 (2011), pp. 1–11.

[10] David Kipling et al. “What can progeroid syndromes tell us about human aging?” In: *Science* 305.5689 (2004), pp. 1426–1431.

[11] Romina Burla et al. “Genomic instability and DNA replication defects in progeroid syndromes”. In: *Nucleus* 9.1 (2018), pp. 368–379.

[12] Mattheus Xing Rong Foo, Peh Fern Ong, and Oliver Dreesen. “Premature aging syndromes: From patients to mechanism”. In: *Journal of Dermatological Science* 96.2 (2019), pp. 58–65.

[13] George M Martin. “Syndromes of accelerated aging”. In: *Natl Cancer Inst Monogr* 60 (1982), pp. 241–247.

[14] Dido Carrero, Clara Soria-Valles, and Carlos López-Otín. “Hallmarks of progeroid syndromes: lessons from mice and reprogrammed cells”. In: *Disease models & mechanisms* 9.7 (2016), pp. 719–735.

[15] Satya N Majumdar, Sanjib Sabhapandit, and Grégory Schehr. “Random walk with random resetting to the maximum position”. In: *Physical Review E* 92.5 (2015), p. 052126.

[16] Lenore Cowen et al. “Network propagation: a universal amplifier of genetic associations”. In: *Nature Reviews Genetics* 18.9 (2017), pp. 551–562.

[17] Hagai Levi, Ran Elkon, and Ron Shamir. “DOMINO: a network-based active module identification algorithm with reduced rate of false calls”. In: *Molecular systems biology* 17.1 (2021), e9593.

[18] Sepideh Sadegh et al. “Exploring the SARS-CoV-2 virus-host-drug interactome for drug repurposing”. In: *Nature communications* 11.1 (2020), p. 3518.

[19] Susan Dina Ghiassian, Jörg Menche, and Albert-László Barabási. “A DIseAse MOdule Detection (DIAMOnD) algorithm derived from a systematic analysis of connectivity patterns of disease proteins in the human interactome”. In: *PLoS computational biology* 11.4 (2015), e1004120.

[20] Judith Bernett et al. “Robust disease module mining via enumeration of diverse prize-collecting Steiner trees”. In: *Bioinformatics* 38.6 (2022), pp. 1600–1606.

[21] Michael Caldera et al. “Interactome-based approaches to human disease”. In: *Current Opinion in Systems Biology* 3 (2017), pp. 88–94.

[22] Paul Jaccard. “Étude comparative de la distribution florale dans une portion des Alpes et des Jura”. In: *Bull Soc Vaudoise Sci Nat* 37 (1901), pp. 547–579.

[23] Carlos López-Otín et al. “The hallmarks of aging”. In: *Cell* 153.6 (2013), pp. 1194–1217.

[24] Andrea G Bodnar et al. “Extension of life-span by introduction of telomerase into normal human cells”. In: *science* 279.5349 (1998), pp. 349–352.

[25] Sara Nicolai et al. “DNA repair and aging: the impact of the p53 family”. In: *Aging (Albany NY)* 7.12 (2015), p. 1050.

[26] Wanbao Yang et al. “Suppression of FOXO1 attenuates inflamm-aging and improves liver function during aging”. In: *Aging Cell* 22.10 (2023), e13968.

[27] Yujia Yuan et al. “Regulation of SIRT1 in aging: roles in mitochondrial function and biogenesis”. In: *Mechanisms of ageing and development* 155 (2016), pp. 10–21.

[28] Cui Chen et al. “SIRT1 and aging related signaling pathways”. In: *Mechanisms of ageing and development* 187 (2020), p. 111215.

[29] Takayoshi Sasako et al. “Deletion of skeletal muscle Akt1/2 causes osteosarcopenia and reduces lifespan in mice”. In: *Nature Communications* 13.1 (2022), p. 5655.

[30] Mei Hua Jin and Do-Youn Oh. "ATM in DNA repair in cancer". In: *Pharmacology & therapeutics* 203 (2019), p. 107391.

[31] CA Cremona and A Behrens. "ATM signalling and cancer". In: *Oncogene* 33.26 (2014), pp. 3351–3360.

[32] Kanmin Mao and Guo Zhang. "The role of PARP1 in neurodegenerative diseases and aging". In: *The FEBS journal* 289.8 (2022), pp. 2013–2024.

[33] Aswin Mangerich, Alexander Bürkle, et al. "Pleiotropic cellular functions of PARP1 in longevity and aging: genome maintenance meets inflammation". In: *Oxidative medicine and cellular longevity* 2012 (2012).

[34] Aravind Subramanian et al. "A next generation connectivity map: L1000 platform and the first 1,000,000 profiles". In: *Cell* 171.6 (2017), pp. 1437–1452.

[35] Richard A Miller et al. "An aging interventions testing program: study design and interim report". In: *Aging cell* 6.4 (2007), pp. 565–575.

[36] Yi-Tsau Huang et al. "Hemodynamic effects of synephrine treatment in portal hypertensive rats". In: *Japanese journal of pharmacology* 85.2 (2001), pp. 183–188.

[37] Luciana Grazziotin Rossato et al. "Synephrine: from trace concentrations to massive consumption in weight-loss". In: *Food and chemical toxicology* 49.1 (2011), pp. 8–16.

[38] Van A Doze et al. "Long-term α1A-adrenergic receptor stimulation improves synaptic plasticity, cognitive function, mood, and longevity". In: *Molecular pharmacology* 80.4 (2011), pp. 747–758.

[39] Francisco Alejandro Lagunas-Rangel. "G protein-coupled receptors that influence lifespan of human and animal models". In: *Biogerontology* 23.1 (2022), pp. 1–19.

[40] Damian Szklarczyk et al. "The STRING database in 2021: customizable protein–protein networks, and functional characterization of user-uploaded gene/measurement sets". In: *Nucleic acids research* 49.D1 (2021), pp. D605–D612.

[41] Anastasia V Shindyapina et al. "Molecular signatures of longevity identify compounds that extend mouse lifespan and healthspan". In: *bioRxiv* (2025), pp. 2025–06.

[42] Valentine M Macaulay et al. "Phase I dose-escalation study of linsitinib (OSI-906) and erlotinib in patients with advanced solid tumors". In: *Clinical Cancer Research* 22.12 (2016), pp. 2897–2907.

[43] Xiaonan Hou et al. "Dual IGF-1R/InsR inhibitor BMS-754807 synergizes with hormonal agents in treatment of estrogen-dependent breast cancer". In: *Cancer research* 71.24 (2011), pp. 7597–7607.

[44] Christian Bär and Thomas Thum. "Changing direction: from therapeutic telomerase inhibition to activation?" In: *Circulation Research* 120.9 (2017), pp. 1393–1395.

[45] Arianna Calcinotto et al. "Cellular senescence: aging, cancer, and injury". In: *Physiological reviews* 99.2 (2019), pp. 1047–1078.

[46] Aura D Urribarri et al. "Inhibition of metalloprotease hyperactivity in cystic cholangiocytes halts the development of polycystic liver diseases". In: *Gut* 63.10 (2014), pp. 1658–1667.

[47] Concepción Laviades et al. "Abnormalities of the extracellular degradation of collagen type I in essential hypertension". In: *Circulation* 98.6 (1998), pp. 535–540.

[48] Brydon L Bennett et al. “SP600125, an anthrapyrazolone inhibitor of Jun N-terminal kinase”. In: *Proceedings of the National Academy of Sciences* 98.24 (2001), pp. 13681–13686.

[49] Marc Ehrlich et al. “Rapid and efficient generation of oligodendrocytes from human induced pluripotent stem cells using transcription factors”. In: *Proceedings of the National Academy of Sciences* 114.11 (2017), E2243–E2252.

[50] Deisy Morselli Gysi et al. “Network medicine framework for identifying drug-repurposing opportunities for COVID-19”. In: *Proceedings of the National Academy of Sciences* 118.19 (2021), e2025581118.

[51] Leonard Guarente, David A Sinclair, and Guido Kroemer. “Human trials exploring anti-aging medicines”. In: *Cell Metabolism* (2023).
